# The 27th Scientific Conference of the Society on NeuroImmune Pharmacology: New Delhi, India, March 15–18, 2023

**DOI:** 10.1515/nipt-2023-0006

**Published:** 2023-04-10

**Authors:** Sulie L. Chang, Pankaj Seth, Jun Zhu, Gurudutt Pendyala, Jean M. Bidlack, Santosh Kumar

**Affiliations:** Department of Biological Sciences, Institute of NeuroImmune Pharmacology, Seton Hall University, South Orange, NJ, USA; Molecular and Cellular Neuroscience, Neurovirology Section, National Brain Research Centre, Gurugram, Haryana 122052, India; Department of Drug Discovery and Biomedical Sciences, College of Pharmacy, University of South Carolina, Columbia, SC, USA; Department of Anesthesiology, College of Medicine, University of Nebraska Medical Center, 984455 Nebraska Medical Center, Omaha, NE 68198, USA; Department of Pharmacology and Physiology, University of Rochester, School of Medicine and Dentistry, Rochester, NY, USA; Department of Pharmaceutical Sciences, College of Pharmacy, University of Tennessee Health Science Center, 881 Madison Ave, Memphis, TN 38163, USA

**Keywords:** alcohol, behavior, drug abuse, HIV, immunology, in-silico study, neuroscience, pharmacology, viral infection

## Abstract

The 27th Scientific Conference of the Society on Neuroimmune Pharmacology (SNIP) in New Delhi, India, on March 15–18, 2023 is a historic summit of experts from around the world. The four day conference provides insights into the latest and most advanced science in the intersecting areas of neuroscience, immunology, pharmacology, and its translational aspects, in particular, HIV and drug abuse. Abstracts are ordered in three major groups: (1) Symposium speakers (S1–S64), (2) Investigator Posters (I1–I18), and (3) Trainee Poster (T1–T28).

## Introduction

The Society on NeuroImmune Pharmacology (SNIP) originally designed and planned the New Delhi meeting as its 26th SNIP annual meeting in 2020. During the COVID-19 pandemic in 2020–2022, the society postponed the New Delhi meeting and organized a virtual workshop on COVID-19 in 2021, in part, to maintain the consistency of SNIP activity. In 2022, SNIP conducted their first in-person annual meeting in Memphis, Tennessee as its 26th SNIP annual meeting, the first since the pandemic began. It covered a wide array of scientific activities including several symposia, sessions for early career investigators (ECI), and other activities. At its Memphis meeting, SNIP decided to host their postponed New Delhi meeting as its 27th annual conference at the LaLit in New Delhi on March 15–18, 2023.

Thanks to the efforts of Dr. Pankaj Seth, the SNIP New Delhi meeting was co-organized with the internationally renowned National Brain Research Centre (NBRC) at Manesar (Gurgaon), India. On March 15th, there was a pre-conference section held at NBRC. Dr. Krishanu Ray, Director, NBRC welcomed the delegates to NBRC and delivered an opening remark prior to the symposium organized by NBRC entitled “Neuroinflammation and Autophagy” that was co-chaired by Dr. Anirban Basu, Professor, NBRC, Manesar and Dr. Luay Rashan, Professor, Dhofar University, Salalah, Oman. This NBRC symposium covered six scientific talks, respectively, by Dr. Manjula Kalia, Regional Centre of Biotechnology, Dr. Sunit Singh, Professor, Banaras Hindu University, Dr. Jayasri Das Sarma, Professor, Indian Institute of Scientific Education and Research-Kolkata, Dr. Ellora Sen, Senior Scientist, NBRC, Dr. Saravana Babu Chidambaram, JSS College of Pharmacy, Mysuru, and Dr. Sourav Banerjee, Senior Scientist, NBRC. Then, NBRC hosted a Centre tour, group photos, and lunch for the SNIP conference delegates on campus. Following these networking events, NBRC welcome a symposium, titled, “The Brain-immune Axis Portfolios” to highlight the neuroimmune pharmacology research portfolios at National Institutes of Health (NIH), USA. This NIH symposium consisted of the talks by Dr. Roger Sorensen, former Branch Chief, Integrative Neuroscience Branch, Division of Neuroscience and Behavior, National Institute on Drug Abuse, NIH, Dr. Mohammed Akbar, Program Director, National Institute on Alcohol Abuse and Alcoholism, NIH, Dr. Vasudev R Rao, Neuropathogenesis/Therapeutics/SBIR-STTR Programs, Division of AIDS Research, National Institute of Mental Health, NIH. The NBRC events concluded with transportation of SNIP delegates from NBRC campus to The LaLit Hotel in New Delhi.

The official registration was opened at The LaLit in the mid-afternoon on March 15th. At the same time, the delegates were attending the Poster Session that was co-chaired by Drs. Gurudutt Pendyala, Jun Zhu, and Pankaj Seth. Following the Poster Session, Drs. Sulie L. Chang, the Acting President of the 2023 SNIP meeting and Santosh Kumar, the President of SNIP together offered their warmest welcoming hospitality to the Society with an Indian Classical Kathak Dance that was performed by the disciples of Pt. Kishan Maharaj, New Delhi, India, followed by musical performance, “Immortal Classics of Bollywood”, by Mrs. Asawari (Anna) Maggirwar, USA. The delegates will cherish the beauty of the classic Indian music and dance for many years to come. The magnificent cultural and artistic highlight at the 27th conference was followed by the Diversity and Inclusion SNIP Committee (DISC) session that was chaired by Dr. Andrea Raymond, Associate Professor, Herbert Wertheim School of Medicine, Florida International University. The DISC session consisted of a special talk by Dr. Thirumala-Devi Kanneganti, Member, St. Jude Faculty, Vice-Chair, Immunology Department, Rose Marie Thomas Endowed Chair, St. Jude Children’s Research Hospital and six podium presentations by the DISC-ECI Travel Awardees.

On March 16, the 27th SNIP conference was formally inaugurated by Dr. Santosh Kumar, the SNIP President and Professor, Pharmaceutical Sciences and Pharmacology, Addiction Science, and Toxicology, Assistant Dean, Scholarly Integration and Collaboration, College of Pharmacy, UTHSC. In addition to the routine logistic highlights, Dr. Kumar pointed out, unlike in the previous 26 SNIP annual conferences, the 27th SNIP meeting had six plenary lectures representing various SNIP research focuses. Thus, SNIP began a new tradition that the SNIP Presidents deliver the Conference Plenary Lecture and President Plenary Lecture, respectively. Dr. Kumar delivered the Conference Plenary Lecture, titled, “The Past, Present, and Future of the Society on NeuroImmune Pharmacology”. Dr. Kumar’s conference lecture was followed by the President Plenary Lecture titled, “Integration of *in silico*, *in vitro*, and *in vivo* studies in biomedical sciences” that was delivered by Dr. Sulie L. Chang, the Acting President of the 27th SNIP and the Past President of SNIP who is Director, Institute of NeuroImmune Pharmacology and Professor, Department of Biological Sciences, Seton Hall University. Dr. Chang highlighted the powerful integration of *in silico* study with *in vivo* or *in vitro* study as one of the potential future directions of the Society on NeuroImmune Pharmacology. This new tradition has charged the SNIP President to engage in more than a business manager for the Society and take the role as a scientific research leader of the Society.

Following their lectures, Drs. Kumar and Chang co-chaired the Presidential Symposium titled, “Neuroimmune Signaling in Health and Alcohol Use Disorders”. The four talks covered a wealth of neuroimmunology and its role in HIV and Alcohol Use Disorders. The first lecture, titled, “Adolescent alcohol increases brain neuroimmune signaling across microglia and neurons altering adult cellular and behavioral phenotypes”, was delivered by Dr. Fulton T. Crews, John Andrews Distinguished Professor, Pharmacology and Psychiatry, Director, Bowles Center for Alcohol Studies, School of Medicine, University of North Carolina at Chapel Hill. The second lecture was, “Complement Systems Participate in Exosomes Mediated Neurotoxicity”, by Dr. Dipak Sarkar, Board of Governors and Distinguished Professor, Department of Animal Sciences, Director, Endocrinology Program, The State University of New Jersey. The third lecture was, “HIV Infection and the Aging Brain: Contributions from Comorbidities, Nutrition, and Peripheral Systems”, by Dr. Adolf Pfefferbaum, Professor Emeritus, Stanford University School of Medicine, Stanford, Distinguished Scientist and Senior Director of the Neuroscience Program, Center for Health Science, SRI International. The Presidential Symposium was concluded with the lecture, “Emerging Epigenetic Mechanisms Underlying Alcohol Use Disorders”, by Dr. Subhash C Pandey, Professor, Department of Psychiatry, Director, Alcohol Research Center, University of Illinois at Chicago.

In addition to the two Presidents’ plenary lectures and Presidential Symposium, the sessions on March 16th consisted of two additional symposia, a Microbiome Workshop, a Meet the Mentors Lunch session, the first lecture in special US-Indo Plenary Lecture session, Addiction Medication Plenary Lecture and a special talk. The Meet the Mentors Lunch is a unique SNIP traditional session to help mentor our ECI, both pre- and post-doctorate fellows, by many career mentors from different backgrounds (academia, government including NIH and industry). The session helped our ECI to learn about themselves and prepare for their future careers. The Addiction Medication Plenary Lecture was delivered by Dr. Barbara Mason, Pearson Family Professor, Department of Molecular Medicine, Director, Pearson Center on Alcoholism and Addiction Research, Scripps Research. She delivered the lecture on “Alcohol Use Disorders and Potential Treatment”. The special talk on March 16th was presented in the memory of Dr. Bill Narayan, a legend in the field. The Bill Narayan lecture was delivered by SNIP President-Elect Dr. Rosemarie Booze, Professor and Bicentennial Endowed Chair of Behavioral Neuroscience, Department of Psychology, University of South Carolina. She delivered the lecture on “HIV-1 Associated Neurocognitive Disorders (HAND) Following Chronic Microglial Infection and Viral Protein Production”. The first lecture in the special US-Indo Plenary Lecture session, “Alcohol and Stress-Immune Axis: Impact on Alcohol Relapse and Pharmacotherapy Outcomes”, was presented by Dr. Rajita Sinha, Foundations Fund Professor in Psychiatry, Neuroscience and Child Study; Chief, Psychology Section in Psychiatry; Yale University School of Medicine.

The second symposium on March 16, “Theranostics”, was organized by Dr. Howard Gendelman, Professor and Chair, Department of Pharmacology and Experimental Neuroscience (PEN), University of Nebraska Medical Center. The symposium covered the following presentations. The first lecture, “Exosome Drug Delivery and NeuroHIV Immunotheranostics”, was delivered by Dr. Santhi Gorantla, Professor, PEN. The second lecture, “Theranostics for HIV/AIDS”, was delivered Dr. Howard Gendelman. The third lecture, “Pathways to HIV-1 Elimination”, was delivered by Dr. Prasanta Kumar Dash, Assistant Professor, PEN, University of Nebraska Medical Center. The symposium highlighted the development, delivery and impact of Ultra-Long-Acting Antivirals as a means to suppress then eliminate signs of viral infections.

The third symposium, “Substance Abuse, HIV Infection, and Associated Disorders”, was organized and co-chaired by Dr. Navneet Dhillon, Professor, Pulmonary and Critical Care Medicine, Director, Pulmonary Research, University of Kansas Medical Center, and Dr. Siddappa Byrareddy, Professor & Vice Chair of Research, Department of Pharmacology and Experimental Neuroscience, University of Nebraska Medical Center. This symposium had three lectures. The first lecture, “Substance Abuse and HIV Associated Pulmonary Vascular Disease”, was delivered by Dr. Navneet Dhillon. The second, “Methamphetamine potentiation of SARS-CoV-2 N-protein-induced neuroinflammation via NLRP3 inflammasome activation in rat microglia”, was delivered by Dr. Debashis Dutta, Instructor, PEN Department, University of Nebraska Medical Center. And the last lecture, “Drugs of Abuse and SIV Reservoirs”, was delivered by Dr. Siddappa Byrareddy, Professor & Vice Chair of Research, PEN Department, University of Nebraska Medical Center.

The Microbiome Workshop, “The Microbiota Modulation of the Neuronal-Immune System”, was organized and chaired by Dr. Sabita Roy, Professor, Department of Surgery, University of Miami Health System. This workshop had two presentations. The first one was a lecture on “Fecal microbiome transplant from alcoholic hepatitis (AH) patients induces inflammatory changes in gut-brain-immune axis in mice” by Dr. Shirish S. Barve, Professor, Department of Medicine and Pharmacology and Toxicology, University of Louisville. The second was a short talk about how “Prescription opioid induces changes in intestinal microbiome and worsens Inflammatory Bowel Disease” by Dr. Umakant Sharma, Assistant Professor, Department of Surgery, University of Miami Health System.

The March 17, Day 3 sessions consisted of four symposia, a Nanotechnology Plenary Lecture, a special talk, a second special US-Indo Plenary Lecture session, a business meeting, and the 2023 SNIP award ceremony. As the first session of the day, the Nanotechnology Plenary Lecture was delivered by Dr. Madhavan Nair, Distinguished Professor and Founding Chair, Department of Immunology and Nano-Medicine, Associate Vice President of Nanomedicine, Associate Dean of Biomedical Research, Director, Institute of NeuroImmune Pharmacology, Herbert Wertheim College of Medicine, Florida International University. He delivered the talk, “Getting into the Brain: Use of Nanotechnology in Drug Abuse Research”. The second lecture was presented in the memory of Dr. Adarsh Kumar, who had been an ambassador of SNIP young investigators. The Adarsh Kumar lecture was delivered by Dr. Michal Toborek, Professor and Vice Chair of Research, Department of Biochemistry & Molecular Biology, Miller School of Medicine, University of Miami. His lecture was on “Cerebrovascular Pathology of HIV Infection and Drug Abuse”. The second lecture in the special US-Indo Plenary Lecture session, “Autoimmune Epilepsies”, was presented by Dr. Manjari Tripathi, Professor, Department of Neurology, All India Institute of Medical Sciences, New Delhi, India. The business meeting was run by Drs. Santosh Kumar and Sulie L. Chang. The meeting was attended by all the SNIP members. This is where we propose new ideas, revisit the SNIP by-laws and guidelines and make necessary changes as appropriate, and approve them through our membership voting mechanism. The final session was the hallmark of the SNIP annual meeting where we transitioned from the current councils to the new elected councils. The session was also highlighted by many thank you and awards to ECI pre-and post-doctoral fellows in the oral presentation and poster categories, as well as SNIP awards to the elected awardees.

The first symposium on March 17, **“**Extracellular Vesicles in Substance Abuse and Neurological Disorders”, was organized and co-chaired by Dr. Andrea D. Raymond, Associate Professor, Herbert Wertheim School of Medicine, Florida International University and Dr. Ilker Sariyer, Associate Professor, Department of Microbiology, Immunology, and Inflammation, Lewis Katz School of Medicine, Temple University. The symposium had four presentations. The first lecture, “Brain Endothelial Cell (EC) Microvesicle Release during HIV Infection”, was delivered by Mr. Jonathan Hale, Assistant Scientist, Pathology and Laboratory Medicine, Lewis Katz School of Medicine, Temple University. The second lecture, “Molecular and Cellular Effects of Morphine and Nef-EVs on Alternative Splicing of OPRM1”, was delivered by Dr. Ilker Sariyer. The third lecture, “Exosomal Extracellular Vesicles are Potential Indicators of HIV-Associated Impairment Status”, was delivered by Dr. Andrea Raymond. The symposium was concluded with the lecture, “Neuropathogenic Role of Astrocyte-Derived Extracellular Vesicles in HIV Associated Neurocognitive Disorders”, delivered by Dr. Susmita Sil, Assistant Professor, Pharmacology & Experimental Neuroscience, University of Nebraska Medical Center.

The second symposium, “Drug Abuse, Alcohol, and Medication”, was organized and co-chaired by Dr. Syed Ali, Senior Research Scientist, Center for Integrative Nanotechnology Sciences, University of Arkansas at Little Rock, President & CEO, NeuroLab International, PLLC and Dr. Mohammed Akbar, Program Director, National Institute on Alcohol Abuse and Alcoholism, NIH. The symposium covered four talks. The first lecture, “Effects of Cannabidiol on Cocaine Seeking: Dose-Response Profiles and Neurobiological Substrates”, was delivered by Dr. Friedbert Weiss, Professor and Principal Investigator, Department of Neuroscience, The Scripps Research Institute. The second lecture titled “Therapeutic Use of Ketamine in Psychiatric Disorders” was delivered by Dr. Hwei-Hsien Chen, Investigator, National Health Research Institute (NHRI). The third lecture, “Marijuana: Potential Treatment of Neuropsychiatric Disorders”, was delivered by Dr. Emmanuel Onaivi, Professor, Department of Biology, Wayne Paterson University. The fourth lecture, “Sexual Dimorphism in the Effect of Commonly used Anesthetics on the Vasoactive Properties of Alcohol and Tetrahydrocannabinol in the Brain”, was delivered by Dr. Anna Bukiya, Professor, Department of Pharmacology, Addiction Science and Toxicology, The University of Tennessee Health Science Center.

The third symposium, “Autophagy and Neuroinflammation: From Mechanism to Therapeutic Opportunities”, was organized and co-chaired by Dr. Shilpa Buch, Professor, Department of Pharmacology and Experimental Neuroscience, University of Nebraska medical Center and Dr. Yuri Persidsky, Professor and Chair, Department of Pathology and Laboratory Medicine, Lewis Katz School of Medicine, Temple University. The symposium covered four talks. The first lecture, “Gut Immune and Microbial Changes in Response to Burn Injury”, was delivered by Dr. Mashkoor Choudhry, Professor, Department of Microbiology and Immunology, Loyola University. The second lecture, “Cocaine-mediated microglial activation involves dysregulated auto/mitophagy”, was delivered by Dr. Shilpa Buch. The third lecture, “Brain and Lung Injury caused by Alcohol and Electronic Cigarettes: Mechanisms of Deleterious Effects on Blood Brain and Alveolar-Endothelial Barriers”, was delivered by Dr. Yuri Persidsky. The symposium concluded with the lecture, “HIV-1 Tat and Morphine-Mediated Astrocytes involves Epigenetic Modifications of the NLRP6 Inflammasome”, delivered by Dr. Palsamy Periyasamy, Assistant Professor, Department of Pharmacology and Experimental Neuroscience, University of Nebraska.

The final symposium was dedicated to the ECI Travel Awardees, which were co-chaired by Dr. Gurudutt Pendyala, Robert Lieberman Professor of Anesthesiology, Department of Anesthesiology, University of Nebraska Medical Center and Dr. Natalie May Zahr, Assistant Professor, Department of Psychiatry and Behavioral Sciences, Stanford University School of Medicine. The symposium began with Dr. Sulie L. Chang’s introduction of Dr. Gurudutt Pendyala who provided a keynote lecture titled, “Career in Academic REsearch: Plan with CARE”, which was followed by 5 pre-doctorate and 5 post-doctorate fellows. The ECIs were: Brita Ostermeier, Graduate Student, The George Washington University; Sandip Godse, Graduate Student, The University of Tennessee Health Science Center; Mason Rodriguez, Graduate Student, University of South Carolina; Sarah Davis, Graduate Student, University of South Carolina; Bibek Naik, Graduate student, Meharry Medical College; Trent Bullock, Graduate Student, Temple University; Michelle Mack, Graduate Student, Seton Hall University; Madhav Sharma, Graduate Student, Indian Institute of Science Education and Research; Bindu, Graduate Student, National Brain Research Centre; Seema Singh, Post-doctoral Fellow, University of Nebraska Medical Center. They presented their respective talks for 5 min each followed by a collective Q/A session. The session was the highlight of our ECI training and their progress towards their graduation/fellowship. The best talk from each category was chosen for the “best talk awards” and was presented during the award ceremony at the conclusion of the meeting.

The March 18 sessions consisted of a Neurology and Therapeutics Plenary Lecture, a general symposium and a Panel discussion for junior and mid-career investigators. The plenary lecture was delivered by Dr. Kalipada Pahan, Professor of Neurological Sciences, Biochemistry and Pharmacology, Floyd A. Davis, MD, Endowed Chair in Neurology, Rush University Medical Center, Research Career Scientist, Department of Veterans Affairs, Jesse Brown VA Medical Center. He delivered his talk on “Novel Hippocampal Drugs for Alzheimer’s Disease”. The general symposium was organized and co-chaired by Dr. Jun Zhu, Professor, Department of Drug Discovery and Biomedical Sciences, College of Pharmacy, University of South Carolina and Dr. Loyda Melendez, Department of Microbiology and Zoology, University of Puerto Rico-School of Medicine. The symposium included a keynote talk followed by nine talks. The keynote talk, “Clonal Hematopoiesis in Monocytes contributes to HIV-Associated Neuroinflammation”, was delivered by Dr. Sanjay Maggirwar, Professor and Chair, Department of Microbiology, Immunology, and Tropical Medicine, School of Medicine and Health Sciences, The George Washington University. The first lecture, “Extracellular Vesicle-Mediated Amyloid Transfer and Intercellular Communication within the Neurovascular Unit”, was delivered by Ms. Olivia Osborne, BS, Graduate student, Biochemistry and Molecular Biology, University of Miami School of Medicine. The second lecture, “Genetic Validation of Drug Targets: HIV-1 Tat Protein-Induced Inhibition of [^3^H] Dopamine Uptake in the Prefrontal Cortex of Inducible Tat Transgenic Mice is attenuated in Dopamine Transporter Y88F Knock-in Mice harbored within the Tat Transgenic Mice”, was delivered by Dr. Jun Zhu. The third lecture, “Effects of Azithromycin on Expression and Function of Drug Efflux Transporters in Cellular reservoirs”, was delivered by Dr. Theodore J Cory, College of Pharmacy, University of Tennessee Health Science Center. The fourth lecture, “Glycogen synthase kinase 3: a promising therapeutic target in neuroHIV and HAND”, was delivered by Dr. Shamsudheen Moidunny, Research Assistant Professor, Department of Surgery – Oncology Molecular Therapeutics, University of Miami School of Medicine. The fifth lecture, “Urinary Bladder MicroRNAs Mediate Pain and Reduce the Severity of Experimental Cystitis”, was delivered by Dr. Udai Singh, Associate Professor, Department of Pharmaceutical Sciences University of Tennessee Health Science Center. The sixth lecture, “Disrupted Interferon Type I Signaling in Plasma, Monocytes and Human Brain Organoids from People Living with HIV with Cognitive Impairment”, was delivered by Dr. Yisel Cantres-Rosario, Assistant Professor, School of Medicine, University of Puerto Rico. The seventh lecture, “Long-acting Nanoformulations prevent Dolutegravir Induced Oxidative Stress in Embryo Brain”, was delivered by Dr. Aditya Bade, Assistant Professor, Department of Pharmacology and Experimental Neuroscience, University of Nebraska Medical Center. The eighth lecture, “CD47 Deletion in Lymphatic Endothelium Augments Arterial Lymphangiogenesis and Attenuates Atherosclerosis”, was delivered by Dr. Bhupesh Singla, Assistant Professor, Department of Pharmaceutical Sciences, The University of Tennessee Health Science Center. The final lecture, “The role of the non-canonical inflammasome in neuroinflammation”, was delivered by Dr. Amal Amer, Professor, Department of Microbial Infection and Immunity and Infectious Disease Institute, Ohio State University.

The panel discussion for junior and mid-career investigators, “Roadway to independence and shielding the mid-career saturation”, was moderated by Dr. Santosh Kumar. The panelists included Drs. Sanjay Maggirwar, Samander Kaushik, and Manjunatha Venkataswamy. The NIH colleagues included Drs. Roger Sorensen, Mohammed Akbar, and Vasudev R Rao.

Overall, the 27th SNIP annual meeting was a full-fledged post-pandemic meeting that began with an important new tradition of the SNIP President delivering the conference plenary lecture in line with multiple plenary lectures in various SNIP research areas. The meeting also highlighted a variety of activities that included a pre-conference session via a collaboration with an internationally renowned research institute, NBRC, many scientific sessions (ten symposia, a workshop and four plenary lectures), two special talks, a poster session, oral talks, a mentoring session for ECI, a DISC session, a panel discussion session, a business meeting, and an award session. Last, not the least, as SNIP’s third international conference, the 2023 SNIP conference successfully presented Indian’s amazing diversity, colorful cultural richness, and incredible hospitality via the beautiful performance of classic music and dancing as well as various networking and social events during and post-conference in New Delhi. The detailed program agenda and abstracts of oral talks and posters are provided below.

## Program

The 27th Scientific Conference of SNIP is hosted by The LaLit, New Delhi, India

**Table:** 

**Tuesday March 14th, 2023**
5:00 pm–6:30 pm	Council Meeting (The Lalit Hotel)
6:30 pm–9:00 pm	Council Dinner (The Lalit Hotel)
**Wednesday March 15, 2023**
9:15 am–9:30 am	**Welcome Address**
	Krishanu Ray, Ph.D., Director National Brain Research Center, Manesar (Gurgaon), India
9:30 am–11:30 am	**Local Symposium: Neuroinflammation and Autophagy**
	National Brain Research Centre (NBRC)
	Introduction
	Pankaj Seth, Ph.D., Professor and Scientist VII, National Brain Research Centre, Gurgaon, Haryana, India
	Co-Chairs
	Anirban Basu, Ph.D, Professor, National Brain Research Centre, Manesar, India
	Luay Rashan, Ph.D., Professor, Dhofar University, Salalah, Oman
	Speakers
	**1. Manjula Kalia**, Ph.D., Regional Centre of Biotechnology, Faridabad, India
	** Title**: Pharmacological modulation of Autophagy as a potential therapeutic for Japanese encephalitis.
	**2. Sunit Singh**, Ph.D., Professor, Banaras Hindu University, Varanasi, India
	** Title**: Viruses: Hijackers of Host microRNAs.
	**3. Jayasri Das Sarma**, Ph.D., Professor, Indian Institute of Scientific Education and Research-Kolkata, India
	**Title**: ERp29 Regulates Murine β-coronavirus Infectivity and Replication by limiting Endoplasmic Reticulum Stress
	and facilitating Connexin 43 trafficking to the cell surface
	**4. Ellora Sen**, Ph.D., Senior Scientist, National Brain Research Centre, Manesar, India
	**Title**: Moonlighting by IDH1 in glioma: Potential Therapeutic targets revealed
	**5. Saravana Babu Chidambaram**, Ph.D., JSS College of Pharmacy, Mysuru & Coordinator, Centre for Experimental
	Pharmacology & Toxicology, JSS Academy of Higher Education & Research, Mysuru, India
	**Title**: Effects of intermittent fasting on gut dysbiosis and post-stroke cognitive impairment
	**6. Sourav Banerjee**, Ph.D., Senior Scientist, National Brain Research Centre, Manesar, India
	**Title**: Trash or Treasure? Long non-coding RNAs in age-regulated memory deficits
11:30 am–12:00 pm	**Technical Talk**
	**Speaker: Mr. Arunkumar Padmanabhan,** Commercial marketing Manager, SLS Merck India
	**Title:** Transforming Neuroscience Research with Ultrasensitive Immunoassays
12:00 pm–12:30 pm	**Tour of Research Facilities at NBRC/Group Photograph**
12:30 pm–1:30 pm	**Lunch at NBRC**
1:30 pm–3:30 pm	**NIH Symposium: The Brain-immune Axis Portfolios at NIH, USA**
	**Chair: Huangui (Hank) Xiong**, MD, Ph.D., Professor, Department of Pharmacology and Experimental Neuroscience,
	College of Medicine, University of Nebraska Medical Center, Omaha, NE, USA
	**Speakers**
	**1. Roger Sorensen**, Ph.D., Former Branch Chief, Integrative Neuroscience Branch, Division of Neuroscience
	and Behavior, National Institute on Drug Abuse, NIH, Bethesda, MD, USA
	**2. Mohammed Akbar**, Ph.D., Program Director, National Institute on Alcohol Abuse and Alcoholism,
	NIH, Bethesda, MD, USA
	**3. Vasudev R Rao**, Ph.D., Neuropathogenesis/Therapeutics/SBIR-STTR Programs, Division of AIDS Research,
	National Institute of Mental Health, NIH, Bethesda, MD, USA
3:30 pm–4:30 pm	**Transfer from NBRC to The Lalit Hotel**
4:30 pm–8:00 pm	**Registration at The Lalit Hotel**
4:30 pm–6:30 pm	**Poster Session**
	Drs. Gurudutt Pendyala, Jun Zhu, and Pankaj Seth
	Both trainee and investigator posters will be presented at this time.
	Posters will remain up until Thursday evening.
6:30 pm–7:30 pm	**Reception**
	Finger snacks and drinks
	**Opening Ceremony**
	Kathak Dance performance
	Disciples of Pt. Kishan Maharaj, New Delhi, India
	Immortal Classics of Bollywood
	Mrs. Asawari Maggirwar, USA
7:30 pm–9:00 pm	Diversity and Inclusion SNIP Counsil (DISC) Session
	**Plenary Lecture**
	**Introduction: Dr. Andrea Raymond**, Ph.D., Associate Professor, Herbert Wertheim School of Medicine, Florida International University, Miami, FL, USA
	**Speaker: Thirumala-Devi Kanneganti**, Ph.D., Member, St. Jude Faculty, Vice-Chair, Immunology Department, Rose Marie Thomas Endowed Chair, St. Jude Children’s Research Hospital, Memphis, TN, USA
	**Title:** Molecular targets for the treatment of inflammatory diseases, infection, and cancer
	**DISC Early Career Investigator (ECI) Travel Award Symposium**
	Speakers:
	**1. Mickensone Andre**, Graduate Student, Florida International University, Miami, FL, USA
	**Title**: Delta-9-tetrahydrocannabinol and HIV: Impact on glia-derived EVs and the Development of Magneto-Exosome Latency Targeting Nanotherapeutic (MELT)
	**2. Danielle Antoine**, Graduate Student, University of Miami, Miami, FL, USA **Title:** Neonatal morphine results in long-lasting alterations to the gut microbiome in adolescence and adulthood in a murine model.
	**3. Oandy Naranjo**, Graduate Student, University of Miami, Miami, FL, USA
	**Title**: Blood brain barrier pericytes and the molecular impact of latent vs active HIV infection
	**4. Florida Owens**, Graduate Student, Florida International University, Miami, FL, USA
	**Title**: The Role of Epigenetics in Mediating Maladaptive Neuronal Changes in HIV and Opioid Drug Abuse
	**5. Nicole Emitt**, Post-doctoral Fellow, University of Minnesota, Minneapolis, MN, USA
	**Title**: Macrophage activation is associated with enhanced rewarding effects to morphine
	**6. Lester Rosario-Rodríguez**, Post-doctoral Fellow, University of Puerto Rico, Puerto Rico
	**Title**: Host factors associated with COVID-19 severity and neurological mechanisms in a Hispanic population
	**7. Silvana Valdebenito**, Post-doctoral Fellow, The University of Texas Medical Branch, Galveston, TX, USA
	**Title**: Role of tunneling nanotubes-like structures during the early events of HIV infection and viral reactivation
	Question and Answer Session
9:00 pm–10:00 pm	**Buffet Dinner**
**Thursday March 16th, 2023**
8:00 am–8:15 am	**Inauguration/welcome by the President**
	Santosh Kumar, Ph.D., Professor, Pharmaceutical Sciences and Pharmacology, Addiction Science, and Toxicology, Assistant Dean, College of Pharmacy, The University of Tennessee Health Science Center, Memphis, TN, USA
8:15 am–8:45 am	**Conference Plenary Lecture**
	**Speaker: Santosh Kumar, Ph.D**. Professor, Pharmaceutical Sciences and Pharmacology, Addiction Science, and Toxicology, Assistant Dean, College of Pharmacy, The University of Tennessee Health Science Center, Memphis, TN, USA
	**Title:** The Past, Present, and Future of the Society on NeuroImmune Pharmacology
8:45 am–9:15 am	**President Plenary Lecture**
	**Speaker: Sulie L. Chang, Ph.D**., Director, Institute of NeuroImmune Pharmacology and Professor, Department of Biological Sciences, Seton Hall University, South Orange, NJ, USA
	**Title:** Integration of in silico, *in vitro*, and *in vivo* studies in biomedical sciences
9:15 am–10:45 am	**Presidential Symposium**
	**Neuroimmune Signaling in Health and Alcohol Use Disorders** **Co-Chairs: Sulie L. Chang, Ph.D**., Director, NeuroImmune Pharmacology, Professor, Department of Biological Sciences, Seton Hall University, South Orange, NJ, USA
	**Santosh Kumar, Ph.D**. Professor, Pharmaceutical Sciences and Pharmacology, Addiction Science, and Toxicology, Assistant Dean, College of Pharmacy, The University of Tennessee Health Science Center, Memphis, TN, USA
	**Speakers**
	**1. Fulton T. Crews, Ph.D.**, John Andrews Distinguished Professor, Pharmacology and Psychiatry, Director, Bowles Center for Alcohol Studies, School of Medicine, University of North Carolina at Chapel Hill, Chapel Hill, NC, USA
	**Title:** Adolescent alcohol increases brain neuroimmune signaling across microglia and neurons altering adult cellular and behavioral phenotypes
	**2. Dipak Sarkar, Ph.D.**, D.Phil., Board of Governors and Distinguished Professor, Department of Animal Sciences, Director, Endocrinology Program, School of Environmental & Biological Sciences, The State University of New Jersey, New Brunswick, NJ, USA
	**Title:** Complement Systems Participate in Exosomes Mediated Neurotoxicity
	**3. Adolf Pfefferbaum, M.D.**, Professor Emeritus, Stanford University School of Medicine, Stanford, Distinguished Scientist and Senior Director of the Neuroscience Program, Center for Health Science, SRI International, Menlo Park, CA, USA
	**Title**: HIV Infection and the Aging Brain: Contributions from Comorbidities, Nutrition, and Peripheral Systems
	**4. Subhash C Pandey, Ph.D.**, Professor, Department of Psychiatry, Director, Alcohol Research Center, Senior Research Career Scientist, University of Illinois at Chicago & Jesse Brown VA Medical Center, Chicago, IL, USA
	**Title:** Emerging Epigenetic Mechanisms Underlying Alcohol Use Disorders
10:45 am–11:15 am	**Coffee/Tea Break/Networking**
11:15 am–12:45 pm	**Symposium 1: Theranostics**
	**Chair: Howard Gendelman, M.D**., Professor and Chair, Department of Pharmacology and Experimental Neuroscience (PEN), University of Nebraska Medical Center, Omaha, NE, USA
	**Speakers**
	**1. Santhi Gorantla, Ph.D.,** Professor, Department of Pharmacology and Experimental Neuroscience, University of Nebraska Medical Center, Omaha, NE, USA
	**Title:** Exosome Drug Delivery and NeuroHIV Immunotheranostics
	**2. Howard Gendelman, M.D.,** HIV/AIDS. Professor and Chair, Department of Pharmacology and Experimental Neuroscience, University of Nebraska Medical Center, Omaha, USA
	**Title:** Theranostics for HIV/AIDS
	**3. Prasanta Kumar Dash, Ph.D.**, Assistant Professor, Department of Pharmacology and Experimental Neuroscience, University of Nebraska Medical Center, Omaha, NE, USA
	**Title:** Pathways to HIV-1 Elimination
12:45 pm–2:00 pm	**Meet the Mentors (MTM) Lunch**
	**Co-Chairs: Gurudutt Pendyala, Ph.D.,** Robert Lieberman Professor of Anesthesiology, Department of Anesthesiology, University of Nebraska Medical Center, Omaha, NE, USA. **Yisel Cantres-Rosario, Ph.D.,** Assistant Professor, School of Medicine, Univ. of Puerto Rico, Medical Sciences Campus, San Juan, PR
2:00 pm–2:30 pm	**Bill Narayan Memorial Lecture**
	**Introducer: Sanjay Maggirwar, Ph.D**., Professor and Chair, GW Department of Microbiology, Immunology, and Tropical Medicine, The George Washington University, Washington, DC, USA
	**Speaker: Rosemarie Booze, Ph.D**., Professor and Bicentennial Endowed Chair of Behavioral
	Neuroscience, Department of Psychology, University of South Carolina, Columbia, SC, USA
	**Title:** HIV-1 Associated Neurocognitive Disorders (HAND) Following Chronic Microglial Infection and Viral Protein Production
2:30 pm–3:00 pm	**Addiction Medication Plenary Lecture**
	**Introducer: Syed Ali, Ph.D**., Senior Research Scientist, Center for Integrative Nanotechnology Sciences, University of Arkansas at Little Rock, President & CEO, NeuroLab International, PLLC, Little Rock, AR, USA
	**Speaker: Barbara Mason, Ph.D.,** Pearson Family Professor, Department of Molecular Medicine, Director, Pearson Center on Alcoholism and Addiction Research, Scripps Research, La Jolla, CA, USA
	**Title:** Alcohol Use Disorders and Potential Treatment
3:00 pm–3:30 pm	**Coffee/Tea Break/Networking**
3:30 pm–4:00 pm	**US-Indo Plenary Lecture 1**
	**Introducer: Michelle L. Mack, MS.,** Institute of NeuroImmune Pharmacology, Seton Hall University, South Orange, NJ, USA
	**Speaker: Rajita Sinha, Ph.D.,** Foundations Fund Professor in Psychiatry, Neuroscience and Child Study; Chief, Psychology Section in Psychiatry; Director, Yale Stress Center; Co-Director of Education, Yale Center of Clinical Investigation (Yale Clinical Translational Science Award), Yale University School of Medicine, USA
	**Title:** Alcohol and Stress-Immune Axis: Impact on Alcohol Relapse and Pharmacotherapy Outcomes
4:00 pm–5:15 pm	**Symposium 2: Substance Abuse, HIV Infection, and Associated Disorders**
	**Co-Chairs: Navneet Dhillon, Ph.D.,** Professor, Pulmonary and Critical Care Medicine, Director, Pulmonary Research, University of Kansas Medical Center, Kansas City, KS, USA. **Siddappa Byrareddy Ph.D.,** Professor & Vice Chair of Research, Department of Pharmacology and Experimental Neuroscience, University of Nebraska Medical Center, Omaha, NE, USA
	**Speakers**
	**1. Navneet Dhillon, Ph.D.**, Professor, Pulmonary and Critical Care Medicine, Director, Pulmonary Research, University of Kansas Medical Center, Kansas City, KS, USA
	**Title:** Substance Abuse and HIV Associated Pulmonary Vascular Disease
	**2. Debashis Dutta, Ph.D.,** Instructor, Department of Pharmacology and experimental Neuroscience, University of Nebraska Medical Center, Omaha, NE
	**Title:** Methamphetamine potentiation of SARS-CoV-2 N-protein-induced neuroinflammation via NLRP3 inflammasome activation in rat microglia
	**3. Siddappa Byrareddy Ph.D.,** Professor & Vice Chair of Research, Department of Pharmacology and Experimental Neuroscience, University of Nebraska Medical Center, Omaha, NE, USA.
	**Title:** Drugs of Abuse and SIV Reservoirs
5:15 pm–5:45 pm	**Coffee/Tea Break/Networking**
5:45 pm–6:45 pm	**Microbiome Workshop:** The microbiota modulation of the neuronal-immune system
	**Chair and Preface: Sabita Roy, Ph.D.,** Professor, Department of Surgery, University of Miami Health System, Miami, FL, USA
	**Speakers**
	**1. Shirish S. Barve, Ph.D.,** Professor, Department of Medicine and Pharmacology and Toxicology, University of Louisville, Louisville, KY, USA
	**Title:** Fecal microbiome transplant from alcoholic hepatitis (AH) patients induces inflammatory changes in gut-brain-immune axis in mice
	**2. Dr. Umakant Sharma, Ph.D.,** Assistant Professor, Department of Surgery, University of Miami Health System, Miami, FL, USA
	**Title:** Prescription opioid induces changes in intestinal microbiome and worsens Inflammatory Bowel Disease
7:00 pm–9:00 pm	**Continental Buffet Dinner**
7:00 pm–9:00 pm	**The NIPT Editorial Board Dinner Meeting (Invitation Only**)
**Friday March 17, 2023**
8:00 am–8:30 am	**Nanotechnology Plenary Lecture**
	**Introduction: Santosh Kumar, Ph.D.,** Professor and Assistant Dean, Department of Pharmaceutical Sciences, College of Pharmacy, University of Tennessee Health Science Center, Memphis, TN, USA
	**Speaker: Madhavan Nair, Ph.D., C.N.S., F.A.C.N., F.A.A.A.I.**, Distinguished Professor and Founding Chair, Department of Immunology and Nano- Medicine, Associate Vice President of Nanomedicine, Associate Dean of Biomedical Research, Director, Institute of NeuroImmune Pharmacology, Herbert Wertheim College of Medicine, Florida International University, Miami, FL, USA
	**Title:** Getting into the Brain: Use of Nanotechnology in Drug Abuse Research
8:30 am–10:00 am	**Symposium 3: Extracellular Vesicles in Substance Abuse and Neurological Disorders**
	**Co-Chairs: Andrea D. Raymond, Ph.D., A**ssociate Professor, Herbert Wertheim School of Medicine, Florida International University, Miami, FL, USA. **Ilker Sariyer, Ph.D.,** Associate Professor, Department of Microbiology, Immunology, and Inflammation, Temple University, Lewis Katz School of Medicine, Temple University, Philadelphia, PA, USA
	**Speakers**
	**1. Jonathan Hale, M.A.**, Assistant Scientist, Pathology and Laboratory Medicine, Lewis Katz School of Medicine, Temple University, Philadelphia, PA, USA
	**Title:** Brain endothelial cell (EC) microvesicle release during HIV infection
	**2. Ilker Sariyer, Ph.D.,** Associate Professor, Department of Microbiology, Immunology, and Inflammation, Temple University, Lewis Katz School of Medicine, Temple University, Philadelphia, PA, USA
	**Title**: Molecular and cellular effects of morphine and Nef-EVs on alternative splicing of OPRM1
	**3. Andrea Raymond, Ph.D.**, Associate Professor, Herbert Wertheim School of Medicine, Florida International University, Miami, FL, USA
	**Title:** Exosomal Extracellular Vesicles are potential indicators of HIV-Associated Neurocognitive Impairment Status
	**4. Susmita Sil, Ph.D.,** Assistant Professor, Pharmacology & Experimental Neuroscience, University of Nebraska Medical Center, Omaha, NE, USA
	**Title:** Neuropathogenic role of astrocyte-derived extracellular vesicles in HIV associated neurocognitive disorders
10:00 am–10:30 am	**Coffee/Tea Break/Networking**
10:30 am–12:00 pm	**Symposium 4: Drug Abuse, Alcohol, and Medication**
	**Co-Chairs: Syed Ali, Ph.D.,** Senior Research Scientist, Center for Integrative Nanotechnology Sciences, University of Arkansas at Little Rock, President & CEO, NeuroLab International, PLLC, Little Rock, AR, USA. **Mohammed Akbar, PhD.,** Program Director, National Institute on Alcohol Abuse and Alcoholism/NIH, Bethesda, MD, USA
	**Speakers**
	**1. Friedbert Weiss, Ph.D.,** Professor and Principal Investigator, Department of Neuroscience, The Scripps Research Institute, La Jolla, CA, USA
	**Title:** Effects of cannabidiol on cocaine seeking: dose-response profiles and neurobiological substrates
	**2. Hwei-Hsien Chen, Ph.D.,** Investigator, National Health Research Institute (NHRI), Taiwan
	**Title:** Therapeutic use of ketamine in psychiatric disorders
	**3. Emmanuel Onaivi, Ph.D.,** Professor, Department of Biology, WP University, Patterson, NJ, USA
	**Title:** Marijuana: Potential treatment of neuropsychiatric disorders
	**4. Anna Bukiya, Ph.D.,** Professor, Department of Pharmacology, Addiction Science and Toxicology, The University of Tennessee Health Science Center, Memphis, TN, USA
	**Title:** Sexual dimorphism in the effect of commonly used anesthetics on the vasoactive properties of alcohol and tetrahydrocannabinol in the brain
12:00 pm–1:30 pm	**Open Business Meeting/Lunch**
1:30 pm–2:00 pm	**Adarsh Kumar Memorial Lecture**
	**Introducer: Oandy Naranjo,** Graduate Student, University of Miami, Miami, FL
	**Speaker: Dr. Michal Toborek, M.D. Ph.D.,** Leonard M. Leonard M. Miller Professor and Vice-Chair for Research, Department of Biochemistry and Molecular Biology, Miler School of Medicine, University of Miami, USA
	**Title:** Cerebrovascular pathology of HIV infection and drug abuse
2:00 pm–2:30 pm	**US-Indo Plenary Lecture 2**
	**Introducer: Pankaj Seth, Ph.D.,** Professor, Molecular and Cellular Neuroscience, National Brain Research Centre, Manesar, Haryana, India
	**Speaker: Dr. Manjari Tripathi,** Professor, Department of Neurology, All India Institute of Medical Sciences, New Delhi, India
	**Title:** Autoimmune Epilepsies
2:30 pm–4:00 pm	**Symposium 5: Autophagy and Neuroinflammation: From Mechanism to Therapeutic Opportunities**
	**Co-Chairs: Shilpa Buch, Ph.D.,** Professor, Department of Pharmacology and Experimental Neuroscience, University of Nebraska medical Center, Omaha, NE, USA
	**Yuri Persidsky, MD, Ph.D.,** Professor and Chair, Department of Pathology and Laboratory Medicine, Lewis Katz School of Medicine, Temple University, Philadelphia, PA, USA
	**Speakers**
	**1. Mashkoor Choudhry, Ph.D.,** Professor, Department of Microbiology and Immunology, Loyola University, Chicago, IL, USA
	**Title:** Gut immune and microbial changes in response to burn injury
	**2. Shilpa Buch, Ph.D.,** Professor, Department of Pharmacology and Experimental Neuroscience, University of Nebraska medical Center, Omaha, NE, USA
	**Title:** Cocaine-mediated microglial activation involves dysregulated auto/mitophagy
	**3. Yuri Persidsky, MD, Ph.D.,** Professor and Chair, Department of Pathology and Laboratory Medicine, Lewis Katz School of Medicine, Temple University, Philadelphia, PA, USA
	**Title:** Brain and lung injury caused by alcohol and electronic cigarettes: mechanisms of deleterious effects on blood brain and alveolar-endothelial barriers
	**4. Palsamy Periyasamy Ph.D.,** Assistant Professor, Department of Pharmacology and Experimental Neuroscience, University of Nebraska, Omaha, Nebraska, USA
	**Title:** HIV Tat and morphine-mediated activation of astrocytes involves epigenetic modification of the NLRP6 inflammasome
4:00 pm–4:30 pm	**Coffee/Tea Break/Networking**
4:30 pm–6:00 pm	**Early Career Investigators Travel Awardees Symposium**
	**Introducer: Sulie L. Chang, Ph.D.,** Director, Institute of NeuroImmune Pharmacology and Professor, Department of Biological Sciences, Seton Hall University, South Orange, NJ, USA
	**Keynote speaker: Gurudutt Pendyala, Ph.D.,** Robert Lieberman Professor of Anesthesiology, Department of Anesthesiology, University of Nebraska Medical Center, Omaha, NE, USA
	**Title: C**areer in **A**cademic **RE**search: Plan with CARE
	**Co-Chairs: Gurudutt Pendyala, Ph.D.,** Robert Lieberman Professor of Anesthesiology, Department of Anesthesiology, University of Nebraska Medical Center, Omaha, NE, USA. **Natalie May Zahr, Ph.D.,** Assistant Professor, Department of Psychiatry and Behavioral Sciences, Stanford University School of Medicine, Stanford, CA, USA
	**Speakers**
	**1. Brita Ostermeier,** Graduate Student, The George Washington University, Washington, DC, USA
	**Title:** ECO-HIV infected CHME5 as a novel *in vitro* model for HIV infection and latency in **microglia**
	**2. Sandip Godse,** Graduate Student, The University of Tennessee Health Science Center, Memphis, TN, USA
	**Title:** Development of drug delivery system with antiretroviral and nutraceutical to suppress HIV in brain reservoirs
	**3. Mason Rodriguez,** Graduate Student, University of Southern Carolina, Columbia, SC, USA
	**Title:** HIV associated dysbiosis influences self-administration of cocaine in HIV-1 transgenic rats
	**4. Sarah Davis,** Graduate Student, University of Southern Carolina, Columbia, SC, USA
	**Title:** *In vivo* HIV-1 Tat protein expression alters synaptic dopamine release in a region-specific manner in inducible Tat transgenic mice using fast scan cyclic voltammetry
	**5. Bibek Naik,** Graduate student, Meharry Medical College, Nashville, TN, USA
	**Title:** HIV-1 Integrase Inhibitor associated pathways involved in neuropsychiatric disorders
	**6. Trent Bullock,** Graduate Student, Temple University, Philadelphia, PA, USA
	**Title:** Characterizing the response of hCMEC/D3 Cells to the synthetic cannabinoid 2 receptor agonist, PM289
	**7. Michelle Mack,** Graduate Student, Seton Hall University, South Orange, NJ, USA
	**Title:** Involvement of TRPM7 in alcohol-induced damage of the blood-brain-barrier in the presence of HIV viral proteins
	**8. Madhav Sharma,** Graduate Student, Indian Institute of Science Education and Research, Kolkata, India
	**Title:** IFIT2 restricts murine beta coronavirus induced spinal cord pathology
	**9. Bindu,** Graduate Student, National Brain Research Centre, Gurgaon, India **Title:** The interplay between zika virus-induced autophagy and neural stem cell fate determination
	**10. Seema Singh,** Post-doctoral Fellow, University of Nebraska Medical Center, Omaha, NE, USA
	**Title:** Involvement of LNCRNA XIST/miRNA-124/CCL2 axis in HIV-1 Tat-mediated microglial activation and neuroinflammation
7:00 pm–10:00 pm	**President’s Transition, Banquet, and Awards Ceremony**
	Sulie Chang, Ph.D.
	Santosh Kumar, Ph.D.
	Rosemary Booze, Ph.D.
	Gurudutt Pendyala, Ph.D.
**Saturday March 18, 2023**
8:00 am–8:30 am	**Neurology and Therapeutics Plenary Lecture**
	**Introducer:** Mohammed Bishir, MS, Institute of NeuroImmune Pharmacology, Seton Hall University, South Orange, NJ, USA
	**Speaker: Kalipada Pahan, Ph.D.**, Professor of Neurological Sciences, Biochemistry and, Pharmacology, Floyd A. Davis, M.D., Endowed Chair in Neurology, Rush University Medical Center, Research Career Scientist, Department of Veterans Affairs, Jesse Brown VA Medical Center, Chicago, IL, USA
	**Title:** Novel Hippocampal Drugs for Alzheimer’s Disease
8:30 am–11:00 am	**Symposium 6: General Symposium**
	**Co-chairs: Jun Zhu, MD., Ph.D.**, Professor, Department of Drug Discovery and Biomedical Sciences, College of Pharmacy, University of South Carolina, Columbia, SC, USA. Loyda Melendez, Ph.D., Department of Microbiology and Zoology, University of Puerto Rico-School of Medicine, San Juan, PR
	**Keynote speaker: Sanjay Maggirwar, Ph.D.**, Professor and Chair, Department of Microbiology, Immunology, and Tropical Medicine, School of Medicine and Health Sciences, The George Washington University, Washington, District of Columbia, USA
	**Title:** Clonal Hematopoiesis in monocytes contributes to HIV-associated neuroinflammation
	**Speakers:**
	**1. Olivia Osborne, B.S.**, Graduate student, Biochemistry and Molecular Biology, University of Miami School of Medicine, Miami, FL, USA
	**Title:** Extracellular vesicle-mediated amyloid transfer and intercellular communication within the neurovascular unit
	**2. Jun Zhu, MD, Ph.D.**, Professor, Department of Drug Discovery and Biomedical Sciences, College of Pharmacy, University of South Carolina, Columbia, SC, USA
	**Title:** Genetic validation of drug targets: HIV-1 tat protein-induced inhibition of [3h] dopamine uptake in the prefrontal cortex of inducible tat transgenic mice is attenuated in dopamine transporter Y88F knock-in mice harbored within the tat transgenic mice
	**3. Theodore J Cory, Pharm.D., Ph.D.**, College of Pharmacy, University of Tennessee Health Science Center, Memphis, TN, USA
	**Title:** Effects of azithromycin on expression and function of drug efflux transporters in cellular reservoirs
	**4. Shamsudheen Moidunny, Ph.D.**, Research Assistant Professor, Department of Surgery – Oncology Molecular Therapeutics, University of Miami School of Medicine, Miami, FL, USA
	**Title:** Glycogen synthase kinase 3: a promising therapeutic target in neuroHIV and HAND
	**5. Udai Singh, Ph.D.**, Associate Professor, Department of Pharmaceutical Sciences University of Tennessee Health Science Center, Memphis, TN, USA
	**Title:** Urinary bladder microRNAs mediate pain and reduce the severity of experimental cystitis
	**6. Yisel Cantres-Rosario, Ph.D.**, Assistant Professor, School of Medicine, Univ. of Puerto Rico, Medical Sciences Campus, San Juan, PR
	**Title:** Disrupted interferon type I signaling in plasma, monocytes and human brain organoids from people living with HIV with cognitive impairment
	**7. Aditya Bade, Ph.D.**, Assistant Professor, Department of Pharmacology and Experimental Neuroscience, University of Nebraska Medical Center, Omaha, NE, USA
	**Title:** Long-acting nanoformulations prevent dolutegravir induced oxidative stress in embryo brain
	**8. Bhupesh Singla, Ph.D.**, Assistant Professor, Department of Pharmaceutical Sciences, The University of Tennessee Health Science Center, Memphis, TN, USA
	**Title:** CD47 deletion in lymphatic endothelium augments arterial lymphangiogenesis and attenuates atherosclerosis
	**9. Amal Amer, MD, Ph.D.**, Professor, Department of Microbial Infection and Immunity and Infectious Disease Institute, Ohio State University, Columbus, OH, USA
	**Title:** The role of the non-canonical inflammasome in neuroinflammation
	**Roadway to independence and shielding the mid-career saturation: Panel discussion for junior and mid-career investigators**
11:00 am–12:00 pm	**Moderator: Santosh Kumar, Ph.D., Panelists: Sanjay Maggirwar, Ph.D., Samander Kaushik, Ph.D., Manjunatha Venkataswamy, Ph.D.**, NIH colleagues, and others
12:00 pm–7:30 pm	**Lunch and City tour: Heritage and culture of Delhi and surroundings**
**Sunday March 19, 2023**
	Trip to India’s most visited destination – The Taj Mahal, Agra: Organized by the hotel for the interested people. The delegates pay for this trip at the time of meeting check-in.

## SYMPOSIUM ABSTRACTS S1–S64

Some abstracts for special presentations, guest speakers, and/or by presenter opt-out are not published in this brief report for SNIP’s 26th Scientific Conference.

### Local Symposium: Neuroinflammation and Autophagy

#### 1. PHARMACOLOGICAL MODULATION OF AUTOPHAGY AS A POTENTIAL THERAPEUTIC FOR JAPANESE ENCEPHALITIS

Kalia, M.K., Ph.D. ^1^

^1^Virology, Regional Centre for Biotechnology, Faridabad, 121001.

Japanese encephalitis (JE) is the leading global cause of viral encephalitis with a significant disease burden in India. One-third of JE infections are fatal, and one-third develop permanent neurological sequelae. There is no specific treatment and clinical management is only supportive. Previous studies from our laboratory have established that a cellular homeostatic process Autophagy, becomes dysfunctional during JE infection and that autophagy enhancement through drugs is likely to be neuroprotective. Recent studies have shown the promise of using autophagy inducers to treat neurodegenerative disease conditions. Several FDA approved drugs have been shown to enhance autophagy, and have the potential to be repurposed. We are currently testing novel compounds and FDA approved drugs for their potential to modulate autophagy. The measurement of autophagic flux (rate of degradation) is important to identify novel autophagy inducers and inhibitors. We have established a stable mammalian cell line expressing the fluorescent probe GFPLC3-RFP to evaluate autophagy flux using a high throughput imaging platform. We have utilized this assay to screen a drug library comprising of 2560 compounds to identify autophagy modulators. In addition to known autophagy effectors we have identified several novel autophagy inducers and inhibitors. These are currently being tested for their potential to prevent neuroinflammation and neuronal cell death, and enhancing immune responses *in vitro* and in mouse model of JE. We aim to provide proof of concept for testing these drugs for JE therapy. Supported by DBT/PR27875/MED/29/1302/2018.

#### 2. VIRUSES: HIJACKERS OF HOST MICRORNAS

Singh, S.K., Ph.D. ^1^

^1^Molecular Biology Unit, Faculty of Medicine, Institute of Medical Sciences, Banaras Hindu University, Varanasi, 221005.

MicroRNAs play a key role in the post-transcriptional gene regulation of almost every gene regulatory pathway. Viruses have evolved to take advantage of the microRNAs to affect both cellular and viral gene expressions. Viruses affect the key proteins of the different cellular pathways by exploiting the cellular microRNAs for their immediate gains such as viral infection and persistence either by reducing immune detection, avoiding apoptosis, or promoting lytic or latent infection. Viruses have also evolved the ability to suppress or induce the specific cellular miRNAs in order to enhance their replication, interfere with the immune cell signaling and their entry into cells. We have reported; how human immunodeficiency virus exploits the cellular microRNA expression pattern to breach the blood-brain barrier in a bystander fashion to enter into brain and the mechanisms utilized by Japanese Encephalitis virus to target the important signal transducing adaptor proteins of the immune regulatory networks in human microglial cells as a part of their immune evasion strategy. Viral infection can exert a profound impact on the cellular miRNA expression pattern, and several viruses have been reported to interact directly with cellular microRNAs to exploit these miRNAs to augment their replication potential, viral persistence, cellular invasion, and immune evasion strategies. MicroRNAs offer new insights into understanding the interaction of viruses with their host. Further studies in this direction might open new doors for the development of diagnostics and therapeutics. Supported by Department of Biotechnology (DBT) and Department of Science and Technology (DST), Govt. of India, New Delhi.

#### 3. ERP29 REGULATES MURINE β-CORONAVIRUS INFECTIVITY AND REPLICATION BY LIMITING ENDOPLASMICRETICULUM STRESS AND FACILITATING CONNEXIN 43 TRAFFICKING TO THE CELL SURFACE

Bose, A. ^1^, Kasle, G., Jana, R.^1^, Maulik, M.^1^, Thomas, D.^1^, Mulchandani, V.^1^, Mukherjee, P.^1^, Koval, M.^1^, Sarma, J.D.^1^

^1^Department of Biological Sciences, Indian Institute of Science Education and Research Kolkata, Mohanpur, West Bengal, India

Background and rationale: Astrocytes are inter-connected by gap junctional intercellular communication (GJIC), which isimportant for metabolic coupling in the panglial system. Infection with a neurotropic murine- β-coronavirus (m-CoV), mouse hepatitis virus (MHV-A59) in primary neonatal mouse astrocytes causes intracellular retention of the predominant astrocytic GJ protein, Connexin43 (Cx43), thus impairing GJIC. The study investigated the role of coronavirus-induced ER stress in causing the intracellular retention of Cx43 and revealed an antiviral role for Cx43 and ERp29.

Methods: Primary astrocytes were isolated from neonatal pups of C57BL/6 mice, and DBT cells were infected with MHV-A59. The ER stress markers and an established Cx43 chaperone were checked in the infected cells. Inhibition of ER stress using a well-studied ER stress inhibitor, 4-sodium phenylbutyrate (4-PBA), in infected cells was adopted to check for the rescue of Cx43 trafficking. ERp29 overexpressing cell line DBT-ERp29 was generated to study the importance of Cx43 trafficking in modulating viral spread.

Results: Our studies highlight that intracellular retention of Cx43 in MHV-A59 infected primary astrocytes is associated with increased ER stress marked and reduced expression of ER-resident chaperone, ERp29. Treatment of MHV-infected primary astrocytes with a well-studied ER stress inhibitor 4-sodium phenylbutyrate (4-PBA) rescued their ability to transport Cx43 to the cell surface and increased GJIC by upregulating ERp29 expression. Furthermore, studies on mouse astrocytoma-derived DBT cells stably transfected with exogenous ERp29 showed increased Cx43 trafficking and assembly into GJ plaques. Interestingly, exogenous ERp29 expression in DBT cells restricted mCoV infectivity. Using a 4-PBA and mimetic peptides known to inhibit Cx43 mediated GJIC by binding to extracellular loops of Cx43 and thus demonstrated that ERp29 significantly reduces mCoV infectivity by reducing ER stress and rescuing the trafficking of Cx43 to the cell surface.

Conclusion and Discussion: The current study thus identifies ERp29 as a potential antiviral host factor that can be therapeutically targeted for curbing CoV infection by targeting Cx43 trafficking.

Ethics statement: All experimental procedures and animal care and use were strictly regulated and reviewed per animal ethics approved by the Institutional Animal Care and Use Committee at the Indian Institute of Science Education and Research Kolkata (AUP no.IISERK/IAEC/AP/2019/28.01). Experiments were performed following the guidelines of the Committee for Control and Supervision of Experiments on Animals (CPCSEA), India.

Acknowledgment: This work is supported by research grants from the Department of Biotechnology (DBT), India (DBT/PR20922/MED/122/37/2016) and the Council for Scientific and Industrial Research (CSIR), India (27(0356)/19-EMR-II) to JDS and research funds from IISER-K. Senior Research Fellowships from CSIR supported AB and GK. VM and DT received Institutional Fellowships from IISER-K. MM was supported by DBT-Wellcome Trust India Alliance Early Career Fellowship (Grant no. IA/E/17/1/503659). The authors thank Dr. Kenneth S. Shindler, University of Pennsylvania Scheie Eye Institute, Philadelphia, Pennsylvania, the US, for critically reviewing the work, and Dr. Julian Leibowitz (Texas A&M University College of Medicine, TX, US) for kindly providing the anti-N antibody, and Dr. Stanley Perlman (University of Iowa, Iowa City, IA, US) for his kind gift of DBT cells. The authors also thank the IISER-K confocal imaging facility.

#### 4. MOONLIGHTING BY IDH1 IN GLIOMA: POTENTIAL THERAPEUTIC TARGETS REVEALED

Lathoria, K.^**1**^, Gowda, P.^**1**^, Umdor, S.B.^**1**^, Patrick, P.^**1**^, Suri, V.^**1**^, Sen, E. ^**1**^

^1^National Brain Research Centre, Manesar-122052, India

Mutations in isocitrate dehydrogenase 1 (IDH1) gene – a crucial enzyme of TCA cycle, are associated with better prognosis in gliomas. We show that IDH1 mutational status determines expression of innate-immune molecule long pentraxin 3 (PTX3), which negatively regulates autophagic flux and genes associated with ferritinophagy in gliomas. A diminished expression of PRMT1 (protein arginine methyltransferase 1) and its target asymmetric dimethyl H4R3 mark in IDH1-MT was concomitant with reduced PTX3 in IDH1-MT glioma cells and in patients. PRMT1 mediated epigenetic modification H4R3me2a not only plays an essential role in PTX3 expression, but PRMT1 potentiated the ability of PTX3 to sensitize glioma cells to ferroptosis inducer. This study highlights the non-canonical function of IDH1 in affecting epigenetic-inflammatory (PRMT1-PTX3) landscape in glioma to subsequently determine responsiveness to chemotherapeutics.

#### 5. EFFECTS OF INTERMITTENT FASTING ON GUT DYSBIOSIS AND POST-STROKE COGNITIVE IMPAIRMENT

Chidambaram, S.B., Ph.D. ^1^

^1^Department of Pharmacology, JSS College of Pharmacy, JSS Academy of Higher Education & Research, Mysuru, 570015

^2^Centre for Experimental Pharmacology and Toxicology, Central Animal Facility, JSS Academy of Higher Education & Research, Mysuru, 570015

Recent studies show that intermittent fasting (IFG) improves cellular metabolism and thus enhances brain functions. Here, we hypothesize that gut microbiota mediates intermittent fasting amelioration of disrupted autophagy, proteinopathies and in turn memory in rats subjected to permanent bilateral common carotid artery occlusion. 16 h of fasting (3.00 pm–7.00 am) for 160 days improved spatial memory which correlates to down-regulation of amyloid-β, p-tau and TDP-43 expression in the hippocampal tissue of ischemic rats. On the other hand, IFG upregulated beclin-1, LC3-I/LC3-II, and p62 in hippocampal region. IFG improved the gut microbiota composition and abundance in the ischemic rats. However, in another set of study, rats treated with antibiotic cocktail (vancomycin and metronidazole; twice a week for 160 days) did not show any effects as mentioned above, which indicates that gut microbial population facilitates IFG induced neuroprotection in ischemic rats. Supported by Department of Biotechnology, Ministry of Science and Technology, Govt of India (BT/PR38038/PFN/20/1528/2020).

#### 6. TRASH OR TREASURE? LONG NON-CODING RNAS IN AGE-REGULATED MEMORY DEFICITS

Banerjee, S., Ph.D.^1^

^1^Cellular and Molecular Neuroscience, National Brain Research Centre, Manesar, 122052.

Long non-coding RNAs, that was thought to be a spurious product of transcription, emerged as a critical regulator of various cognitive functions including learning and memory. We have investigated how this novel class of non-coding transcripts regulates memory formation and what happens when this regulatory control is impaired during aging. Our genome-wide screen identified a subset of long non-coding transcripts that are enriched at the hippocampal synapses. We observed an age-dependent and sex specific differential expression of a select group of long non-coding RNAs. These non-coding transcripts regulates protein synthesis locally at specific synaptic sites that is critical for hippocampus-dependent memory formation. Our study showed that sleep deprivation, a pathological condition of aging, negatively regulates protein synthesis involving synapse-enriched long non-coding RNAs. We aim to provide a causal link between age-regulated memory deficits and long non-coding RNA-mediated altered translation in neuronal synapses. Supported by Department of Biotechnology, Government of India.

### DISC-ECITA Symposium

#### 7. TARGETING INNATE IMMUNITY AND INFLAMMATORY CELL DEATH, PANOPTOSIS, FOR THE TREATMENT OF INFLAMMATORY AND INFECTIOUS DISEASES

Kanneganti, T.D., Ph.D.^1^

^1^Department of Immunology, St. Jude Children’s Research Hospital, Memphis, TN 38105.

Innate immunity is the critical first line of defense against disease. Regulated cell death (RCD) is an important innate immune strategy for host survival from infection or homeostatic perturbations. There are non-lytic RCD pathways, such as apoptosis, and lytic RCD pathways, such as the inflammatory pyroptosis and necroptosis. While historically viewed as independent pathways, molecular crosstalk found among them created a gap in our mechanistic understanding of innate immune pathways that drive RCD. This led to identification of a unique innate immune lytic RCD pathway called PANoptosis that is regulated by PANoptosomes upon sensing pathogens, PAMPs, DAMPs or cytokines. Cytosolic innate immune sensors, such as ZBP1, AIM2 and RIPK1, promote PANoptosome assembly and PANoptosis. Caspases, including caspase-8, also have a critical role here. PANoptosis is activated across several diseases, suggesting that targeting it may have therapeutic benefits. For example, we found that increased TNF and IFN-g in SARS-CoV-2 infection leads to PANoptosis and pathology. Neutralizing them reduces SARS-CoV-2–induced mortality in mice, while activating this pathway in cancer with TNF and IFN-g treatment, or IFN and nuclear export inhibitor treatment to modulate ZBP1-ADAR1 regulation for ZBP1-PANoptosome formation, can regress tumors in murine models. Given the impact of innate immune-mediated RCD, and PANoptosis specifically, across the disease spectrum, work to understand PANoptosome components and regulation paves the way to mechanistically target countless PANoptosis-dependent diseases.

#### 8. HIV LATENCY IN GLIA CELLS: EFFECTS OF DELTA-9-TETRAHYDROCANNABINOL AND A MAGNETIC EXOSOME-BASED LATENCY TARGETING (MELT) NANOTHERAPEUTIC

Andre, M., BS ^1^, Yndart, A., BS^1^, Kolishetti, N., Ph.D.^1^, Nair, M., Ph.D.^1^, Raymond, A., Ph.D.^1^

^1^Department of Immunology and Nanomedicine, Herbert Wertheim College of Medicine at Florida International University, Miami, FL 33199.

Antiretroviral drugs (ARVs) have low CNS penetrance due to the inability to cross the blood-brain barrier (BBB) and enter the brain, where glial cells infected with Human Immunodeficiency Virus (HIV) harboring proviruses. Glia cells serve as HIV reservoirs that can reactivate and release new virions. Aside from not impacting these latent reservoirs, ARVs have many side effects, such as nausea, fatigue, high cholesterol, and trouble sleeping. Consequently, HIV patients are prescribed medical marijuana to mitigate chronic pain associated with HIV pathology. Although the antiemetic and anti-inflammatory properties of marijuana are well-known, it is unclear how delta-9-tetrahydrocannabinol (∆-9-THC), the psychoactive component of marijuana, impacts HIV latency in the brain. Here we assess MELT and ∆-9-THC impact on HIV latency reactivation in HC69 cells (a microglial model for HIV latency with a GFP reporter). MELT consists of magnetoelectric nanoparticles and exosomes loaded with an HIV Tat inhibitor (ZL0580) to target latency. The HC69 cells were treated with increasing concentrations of ∆-9-THC and MELT for 24 h. The viability and HIV reactivation were assessed via flow cytometry. Preliminary results show that MELT and ∆-9-THC reduced HIV transcription by almost 90 %. HC69 cells exhibited higher viability when treated with ∆-9-THC than ZL0580-loaded exosomes than cells treated with the drug alone. These findings demonstrate that MELT and ∆-9-THC can effectively block HIV reactivation, suggesting a novel delivery strategy to block and lock HIV reactivation in the brain.

#### 9. BLOOD BRAIN BARRIER PERICYTES AND THE MOLECULAR IMPACT OF LATENT VERSUS ACTIVE HIV INFECTION

Naranjo, Oandy, BS^1^, Torices, S., Ph.D.^1^, Osborne, O., BS^1^, Toborek, M., MD, Ph.D.^1^

^1^Biochemistry and Molecular Biology, University of Miami, Miami, FL 33136.

Successful antiretroviral therapy (ART) has significantly decreased the mortality and morbidity of individuals infected with HIV. Still, low levels of HIV replication persist in viral reservoirs and lead to immune activation and chronic inflammation. The CNS was thought to be protected from HIV infection. However, experiments on microglia and astrocytes indicated that these cells are all capable of active and latent viral infection. We have pioneered research on HIV-1 infection in brain pericytes and indicated that these cells possess the receptor profile enabling latent and active HIV-1 infection. BBB pericytes have been shown to regulate brain paracellular and transendothelial fluid transport at the BBB, maintain homeostasis of the CNS microenvironment, and maintain BBB integrity. We use a novel HIV reporter, named HIVGKO, that allows for purification of latently infected cells in absence of reactivation. Fluorescence-activated cell sorting was performed to isolate active, latent, and uninfected populations of BBB pericytes. Statistical analysis reveals several distinct molecular signatures when comparing latent, active, and uninfected cell populations. Among the most distinct pathways between latent and active infected pericytes are estrogen receptor signaling, mitochondrial dysfunction, and the sirtuin signaling pathway. These findings provide key insights into the molecular signatures affecting HIV infection of BBB pericytes and may help to develop future targets for the treatment of HIV at the neurovascular unit. Supported by NIH R01-MH128022-01, R01 DA050528-02S1.

#### 10. THE ROLE OF EPIGENETICS IN MEDIATING MALADAPTIVE NEURONAL CHANGES IN HIV AND DRUG ABUSE

Owens, F., MS ^1^, El-Hage, N., Ph.D.^1^, Pahwa, S., Ph.D.^2^, Suresh, P., Ph.D.^2^

^1^Immunology and Nanomedicine, Florida International University, Miami, FL 33199

^2^Microbiology and Immunology, University of Miami, Miami, FL 33101.

Long-term repeated exposure to drugs induces alterations to brain areas involved in reward processing and motivation which leaves individuals susceptible to engage in pathological drug-seeking and drug-taking that can persist for a lifetime. HIV-infected individuals are at a higher risk of substance abuse than the general population. Substance abuse increases risk for the spread of HIV infection and is associated with treatment non-compliance, rapid disease progression, and greater mortality. The incidence of HIV and drug abuse among minority population keeps alarmingly rising in South Florida. HIV-associated neurocognitive impairment remains highly prevalent, and drug abuse is the primary factor for this neuropsychological decline. Studies have demonstrated that epigenetic modulations can regulate HIV infection. We previously showed that HIV infection followed by exposure with morphine and antiretrovirals modulate several epigenetic factors in human glial cells. However, how epigenetic modifications and genetic factors impact viral infection in the brain and influence neurological impairments, particularly in an environmental influenced by substance abuse, is incompetently understood. In the current study, we explored potential interactive effects of HIV combined with drug use and investigated changes in expression levels of chromatin modifying enzymes potentially associated with HIV-pathology using postmortem brain tissue and PBMC samples of HIV-infected patients with a history of opioid/polysubstance abuse, and/or neurological impairment.

#### 11. MACROPHAGE ACTIVATION IS ASSOCIATED WITH ENHANCED REWARDING EFFECTS TO MORPHINE

Emmitt, Nicole, Ph.D. ^1^, Krisha, V.D., Ph.D.^1^, Var, S., Ph.D.^3^, Lind, E., Ph.D.^3^, Chen, D., BS^3^, Grande, A.W., MD^3^, Low, W.C., Ph.D.^3^, Cheeran, M.C., Ph.D.^1^

^1^Veterinary Population Medicine, University of Minnesota, St. Paul, MN 55108

^2^Comparative and Molecular Biosciences Graduate Program, University of Minnesota, St. Paul, MN 55108

^3^Department of Neurosurgery, University of Minnesota, Minneapolis, MN 55455.

Adolescents and soldiers with a history of mild traumatic brain injury (TBI) are at 2.5× greater risk for substance use disorder. However, the risk for adverse outcomes following mild TBI in the general population is hard to determine since 69–90 % of TBI’s are mild and are often unreported. Experiments were designed to evaluate how mild TBI affects the reinforcing properties of morphine, using conditioned placed preference (CPP) and IV self-administration tests. CPP for morphine was assessed at 5- and 10-days post injury (DPI) using a dose range from 0.5–5 mg/kg. Mice with TBI established a strong place preference to 0.5 and 1 mg/kg morphine and escalated their intake of morphine when self-administered. To determine the impact of morphine on TBI-induced neuroinflammation, a fixed dose morphine exposure (5 mg/kg, SC, BID) was utilized. Inflammatory cells in the brain were immune phenotyped at 1-, 3-, 7-, and 15-DPI. A biphasic infiltrating macrophage response was observed with increasing cell numbers in injured mice brains at 3- and 15-DPI. At 15-DPI, CD8 lymphocytes were increased in injured mice exposed to morphine, a cell population not previously associated with the immune response to mild TBI. Closer examination of the blood brain barrier at 15-DPI demonstrated increased leakiness in the midbrain. A concurrent decrease in macrophage phagocytic activity was also observed at 15-DPI. This study revealed a unique inflammatory response to morphine after mild TBI. Supported by Mn SC and TBI Research Grant Program (#128515, #159891, and #214546), NIH T32 DA007097, 2019 CVM Resident and Grad Student Grant.

#### 12. HOST FACTORS ASSOCIATED WITH COVID-19 SEVERITY AND NEUROLOGICAL MECHANISMS IN A HISPANIC POPULATION

Rosario-Rodríguez, L.J., Ph.D.^1^, Cantres-Rosario, Y., MS^2^, Rodríguez-De Jesús, A.E., MS^2^, Carrasquillo-Carrión, K., MS^3^, Méndez, L.B., Ph.D.^4^, Roche-Lima, A., Ph.D.^3^, Cadilla, C., Ph.D.^5^, Cantres-Rosario, Y.M., Ph.D.^1^, Casillas-Pagán, K.M., MS^4^, Rivera-Nieves, V.^6^, Bertrán, J., MD^7^, Wojna, V., MD^1^, Meléndez, L.M., Ph.D.^8^

^1^Department of Internal Medicine, University of Puerto Rico-Medical Sciences Campus, San Juan, PR 00935

^2^Translational Proteomics Center, University of Puerto Rico-Medical Sciences Campus, San Juan, PR 00935

^3^Bioinformatics and Health Informatics, University of Puerto Rico-Medical Sciences Campus, San Juan, PR 00935

^4^Department of Science & Technology, Ana G Mendez University, Carolina, PR 00983

^5^Department of Biochemistry, University of Puerto Rico-Medical Sciences Campus, San Juan, PR 00935

^6^Interdisciplinary Studies, Natural Sciences, University of Puerto Rico-Río Piedras Campus, San Juan, PR 00925

^7^Infectious Diseases, Auxilio Mutuo Hospital, San Juan, PR 00919

^8^Department of Microbiology and Medical Zoology, University of Puerto Rico-Medical Sciences Campus, San Juan, PR 00935.

Coronavirus 2019 disease (COVID-19) ranges from asymptomatic to severe disease. We hypothesize that host factors will determine COVID-19 severity. A total of 121 men and women 21–80 yrs-old were recruited in Puerto Rico. Plasma and PBMCs samples were collected from unvaccinated and vaccinated COVID-19 positive and negative controls. DNA was isolated from PBMCs and Global Diversity Arrays were used. Quantitative proteomics were performed in plasma using Tandem Mass Tag (TMT) labeling, Proteome Discoverer, and Limma Statistics. Omics results were analyzed in IPA. Cytokines were quantified in plasma using a human cytokine array. Neuronal and Astrocyte-derived exosomes (ADE) were quantified in plasma by flow cytometry. Preliminary genomics results show that among the top 10 pathways in enrichment analysis for genes with variants of ≥0.50 allele frequency in patients with severe COVID-19 include: Axonal guidance, CREB, and synaptogenesis signaling. Similarly, proteomics revealed that synaptogenesis signaling was inhibited in severe COVID-19. Levels of IL-1Ra, IP-10, RANTES, TNFa, and MIP-1a were increased in COVID19 patients in a severity-dependent manner, whereas PDGF-BB levels were decreased in COVID-19 patients. ADE were increased in plasma of COVID-19 patients. In vaccinated participants, COVID-19 increased levels of Aminopeptidase N, Hemoglobin subunits beta and delta, AGP-1, Haptoglobin, Carbonic anhydrases 1 and 2, and IP-10 in plasma. This study uncovers potential host predictors of COVID-19 severity and mechanisms associated with neurological consequences in Hispanics. Supported by U54MD007600, P20GM103475, U54GM133807, F99NS113455, K00NS11345, K22NS118975, R01NS099036, UPR COVID19, and the UPR-CCC.

#### 13. ROLE OF TUNNELING NANOTUBES-LIKE STRUCTURES DURING THE EARLY EVENTS OF HIV INFECTION AND VIRAL REACTIVATION

Valdebenito, S., Ph.D.^1^, Eugenin, E.A., Ph.D.^1^, Akira, O., Ph.D.^2^, Rong, L., Ph.D.^3^

^1^Neurobiology, University of Texas Medical Branch, GALVESTON, TX 77550

^2^Department of Microbiology & Immunology, University of Michigan Medical School, Ann Arbor, MI 48109

^3^Department of Mathematics, University of Florida, Gainesville, FL 32611.

The HIV pandemic affects 38.5 million worldwide and is still ongoing despite the successful introduction of antiretroviral therapy (ART). However, ART is not a cure due to the early generation of long-lasting viral reservoirs (VRs) in several tissues. Despite all the efforts to eradicate these latently infected cells, their elimination has not been achieved. A critical question is how few VRs can repopulate the entire body in a few weeks post ART intervention. Most models and data indicate that soluble virus is not sufficient to explain reactivation. The identification of the virological synapsis provided an alternative mechanism of cell-to-cell infection, but still, this area of research is unknown. We identified that an additional mechanism of cell-to-cell transmission can be provided by tunneling nanotubes, TNTs. Our hypothesis is that HIV induces TNTs, the viral spread from HIV latently infected cells to uninfected cells. Our results showed that HIV infection induced the formation of TNTs between HIV-infected and uninfected cells. TNTs allows the transfer of infectious agents from HIV-infected cells to uninfected cells. Induction of TNT formation with H2O2 treatment and subsequent microinjection of a mature virus, and viral proteins, Nef, gp120, and Vif, did not result in TNT mediated transmission, except gag, suggesting that TNT is highly selective to the transported cargo. TNTs are a potential target to prevent, revert or block the transfer of infectious/damaging pathogenic mediators. We propose that TNTs are a therapeutic pathway to eliminate VRs. Supported by National Institute of Mental Health grant, MH096625, The National Institute of Neurological Disorders and Stroke, NS105584.

### President Symposium: Neuroimmune Signaling in Health and Alcohol Use Disorders

#### 14. ADOLESCENT ALCOHOL INCREASES BRAIN NEUROIMMUNE SIGNALING ACROSS MICROGLIA AND NEURONS ALTERING ADULT CELLULAR AND BEHAVIORAL PHENOTYPES

Crews, F.T., Ph.D. ^1^, Vetreno, R.P., Ph.D.^1^, Coleman, L.G., MD, Ph.D.^1^, Zou, J., Ph.D.^1^, Qin, L., Ph.D.^1^

^1^Bowles Center for Alcohol Studies, University of North Carolina at Chapel Hill, Chapel Hill, NC 27599.

Emerging studies find long-lasting changes in gene expression in brain following adolescent intermittent ethanol exposure (AIE). AIE increases adult brain proinflammatory genes including HMGB1 and toll-like receptors as well as reducing cholinergic neurons, hippocampal neurogenesis, and rsfMRI connectivity, while increasing drinking and other behaviors associated with risks for alcohol use disorder (AUD). Increased expression of adult brain HMGB1, a cytokine-like protein released by ethanol that stimulates toll-like receptors (TLR) and RAGE receptors, is a key proinflammatory signal that crosses cell types. AIE caused a persistent increase in microglial proinflammatory phenotypes as well as reduced forebrain cholinergic neurons and hippocampal neurogenesis. Changes in neurons and glia were associated with increases in histone and CpG methylation gene silencing markers, and cholinergic neurons show upregulation of RE-1 silencing transcript (REST). To determine if HMGB1, TLR, or RAGE signaling contributed to these adult AIE pathologies, we allowed rats to exercise or treated them with anti-inflammatory indomethacin and galantamine in prevention or reversal of AIE-induced adult pathology experiments. All anti-inflammatory treatments prevented and reversed AIE-induced cognitive impairments as well as increased HMGB1 neuroimmune signaling as well as the loss of adult forebrain cholinergic hippocampal loss of neurogenesis. Reversal of HMGB1 signaling restores both neurons and cognitive dysfunction. AIE increases risks for AUD that persist to adulthood but are reversible. Supported by NIAAA/NADIA.

#### 15. COMPLEMENT SYSTEMS PARTICIPATE IN EXOSOMES MEDIATED NEUROTOXICITY

Sarkar, D.^1^

^1^Endocrinology Program, Rutgers, The State University of New Jersey, New Brunswick, NJ 08558-2368.

Exosomes are small membrane vesicles (ranging from 30 to 150 nm) comprised of a lipid bilayer, transmembrane proteins, and cytosolic components derived from their host cells. Once formed, exosomes can travel to nearby or distant cells and be taken up through a number of mechanisms, including membrane fusion, receptor-mediated endocytosis, and phagocytosis. Once exosomes enter the target cell, their cargo is released into the cytoplasm and can alter cellular homeostasis. This form of signaling allows cells to communicate with each other via exosomes. We determined whether the microglia use extracellular vesicle exosome to communicate with β-endorphin neuron to induce neuronal apoptosis during ethanol’s toxicity. Extracellular vesicles were prepared from hypothalamic tissues collected from postnatal rats fed daily with 2.5 mg/kg ethanol or control milk formula for 5 days or from hypothalamic microglia cells obtained from postnatal rats, grown in cultures for several days and then challenged with ethanol or vehicle for 24 h. Nanoparticle tracking analysis and transmission electron microscopy indicated that these vesicles had the size range and shape of exosomes. Ethanol treatments increased the number and the β-endorphin neuronal killing activity of microglial exosomes both *in vivo* and *in vitro*. Proteomics analyses of exosomes of cultured microglial cells identified a large number of proteins including various complements, which were elevated following ethanol treatment. Proteomics data involving complements were reconfirmed using quantitative protein assays. Mechanistic study revealed that the complement system mediate ethanol activated exosome induced β-endorphin neuronal death via increasing C5b9/MAC deposition and increasing ROS production β-endorphin neurons. Supported by NIH grants R37AA08757, R01 AA028767.

#### 16. HIV INFECTION AND THE AGING BRAIN: CONTRIBUTIONS FROM COMORBIDITIES, NUTRITION, AND PERIPHERAL SYSTEMS

Pfefferbaum, A. ^1^, Zahr, N.M., Ph.D.^1^, Sullivan, E.V., Ph.D.^2^

^1^Center for Health Science, SRI International, Menlo Park, CA 94025

^2^Department of Psychiatry & Behavioral Sciences, Stanford University School of Medicine, Stanford, CA 94305.

The success of HIV treatment has extended the lifespan of people living with HIV/AIDS (PLWH) to near normal levels; about half are age 50+ and at risk for age-related declines. New opportunities for engaging in risky behaviors, including alcohol use disorder (AUD) and drug misuse, also loom as contributors to decline. To track CNS deficits in aging with HIV, we conducted a 14-year, MRI longitudinal investigation of 68 PLWH without AUD, 60 PLWH + AUD, 222 AUD, and 199 controls. Although all three diagnostic groups showed accelerated frontal cortical decline relative to aging controls, comorbidities of alcohol and drug dependence and co-infection with hepatitis C virus exacerbated decline in PLWH. Parietal cortical volumes also declined in the HIV groups and correlated with the VACS index, possibly reflecting neuroinflammatory processes. Support for an inflammatory basis of action derived from an MR spectroscopy study of brain metabolites indexed by high choline levels in HIV + HCV. Competing with this effect was the observation that low striatal choline levels were present in PLWH who had been treated with efavirenz. An additional factor to consider in knowing who develops functional impairments is nutrition, which can be estimated through itemization of subclinical signs of Wernicke’s encephalopathy. This approach revealed graded severity of functional deficits in PLWH with or without AUD depending on the number of signs detected. This constellation of brain structural, biochemical, nutritional, and comorbid factors may work separately or synergistically to accelerate aging. Supported by National Institute on Alcohol Abuse and Alcoholism AA017347, AA005965, AA017923.

#### 17. EMERGING EPIGENETIC MECHANISMS UNDERLYING ALCOHOL USE DISORDERS

Pandey, S.C., Ph.D. ^1^

^1^Center for Alcohol Research in Epigenetics, Dept. of Psychiatry, University of Illinois at Chicago & Jesse Brown VA Medical Center Chicago, Chicago, IL 60612.

Anxiety that appears during withdrawal is crucial in promoting and maintaining alcohol addiction. The amygdala, particularly the central nucleus of amygdala (CeA), is a critical brain region responsible for anxiety and negative affective behaviors and is heavily implicated in alcohol dependence. We utilized an established rat model of alcohol dependence, where we observe anxiety-like behavior following withdrawal from chronic ethanol exposure. Using an unbiased genome-wide transcriptomic approach (RNA-sequencing) in conjunction with weighted gene co-expression network analysis (WGCNA), we identified distinct differential gene expression modules in the amygdala associated with alcohol withdrawal in rats. Further analysis allowed us to identify histone deacetylase 2 (HDAC2) as a critical epigenetic hub regulator of novel gene network pathways, that is increased in the amygdala of rats following withdrawal. We were able to translate these findings in human post-mortem amygdala of AUD subjects. We further followed up on HDAC2 interacting targets and validated the mRNA changes and their epigenetic regulation mechanisms. Using genetic inhibition of HDAC2 (siRNA infusion in the central nucleus of amygdala) and pharmacological inhibition (HDAC inhibitor, suberoylanilide hydroxamic acid) we were able to prevent epigenetic and gene expression changes in the amygdala and development of anxiety-like behaviors associated with alcohol withdrawal in rats. Together, these studies allowed us to identify a new treatment paradigm for preventing negative withdrawal symptoms in AUD. Supported by NIH-NIAAA P50AA022538, RO1AA-010005, UO1AA-019971 (NADIA project) and by the VA Senior Research Career Scientist award to SCP.

### Symposium 1: Theranostics

#### 18. XOSOME DRUG DELIVERY AND NEUROHIV IMMUNOTHERANOSTICS

Gorantla, S., Ph.D.^1^

^1^Department of Phamracology and Experimental Neuroscince, University of Nebraska Medical Center, Omaha, NE.

Antiretroviral drugs (ARVs) and therapeutic RNAs can be delivered by conventional means or through lipid nanoparticle to suppress or eliminate human immunodeficiency virus type one (HIV-1) infection. Nonetheless, what remains to maximize viral suppression and elimination is the delivery to sites of viral infection. To this end and to improve the pharmacokinetics, blood-brain barrier ARV and CRISPR RNA egress, extracellular vesicles (EVs) were being developed as a novel therapeutic delivery system. The EVs have significant advantages here with known bioavailability and biocompatibility, and this delivery platform will facilitate intercellular communication, and the transmission of antiretroviral therapeutics. The translation of ARV-loaded EVs for therapeutic applications will be discussed together with the characterization for composition, therapeutic loading, and stability, for trafficking, intercellular communication, distribution, and molecular information transfer between cells. EV-based therapeutic delivery approach enhanced the antiretroviral efficacy. Platforms for testing were developing in novel neuroHIV animal model systems and to assess neuropathologic outcomes.

#### 19. THERANOSTICS FOR HIV/AIDS

Gendelman, H.E., M.D.^1^

^1^Professor and Chair, Department of Pharmacology and Experimental Neuroscience, University of Nebraska Medical Center, Omaha, NE

This presentation reviews the achievements made to transform HIV/AIDS regimens from daily up to every six months or longer for prevention and or treatment of viral infection, virus-associated inflammation in the setting of opioid use. Published data sets reviews developmental and delivery approaches for ultra-long-acting (ULA) prodrug formulations and viral excision therapies. We now posit that improvements in clinical outcomes can be achieved by defining ULA profiles. These allow pharmacological site targeting, extended duration, scalability, tissue biodistribution, pharmacokinetic, and pharmacodynamic profiles. Improvements in drug safety and in efficient removal of integrated latent HIV proviral DNA will be discussed. In achieving these goals, we now present rigorous GO/NO GO antiretroviral prodrug screening, process chemistry and nanocrystal formulation development. We outline every six month or longer single drug prevention of viral transmission systems. Improvements in composition of lipid nanoparticles to facilitate ULA formulations to improve regimen adherence and reduce viral transmission. Integration, testing, and manufacturing facilities are shown as part of seamless collaborative cooperation. We posit that our creating long-acting parenterally administered slow effective release of ULA antiretroviral formulations will have a profound impact on HIV prevention and treatment strategies for the most vulnerable populations.

#### 20. PATHWAYS TO HIV-1 ELIMINATION

Dash, P.K., Ph.D.^1^

^1^Department of Pharmacology and Experimental Neuroscience, University of Nebraska Medical Center, Omaha, NE 68198.

The elimination of HIV-1 was reported by our group in a subset (29 %) of humanized mice by using a sequential combination of long-acting [dolutegravir (DTG), lamivudine (3 TC), abacavir (ABC), and rilpivirine (RPV)] ARVs and CRISPR-Cas9 mediated gene-editing targeting HIV- LTR-Gag. To further improve the HIV-1 elimination rate we targeted inactivation of host CCR5, a principal HIV-1 co-receptor, using CRISPR-Cas9 during highly viral suppressed condition followed by excision of HIV-1. The sequential use of two CRISPRs after suppression of active viral replication led to restoration of human CD4+ T cells and elimination of replication-competent virus in 58 % of HIV-1 infected humanized mice. Highly sensitive real time quantitative PCR, droplet digital PCR, RNAscope and viral outgrowth assays affirmed viral elimination. HIV-1 was not detected in blood, spleen, lung, kidney, liver, gut, bone marrow, reproductive organ, and brains of virus-free animals. No evidence of off-target toxicities was recorded in any of the treated animals. Altogether these observations underscore a pivotal role of combinatorial gene editing in achieving the elimination of HIV-1. To determine the molecular signature of viral rebound we employed single-genome (SG) and next-generation sequencing (NGS) on the RNA isolated from plasma samples from replicate dual-treated animals. Sequencing and bioinformatic analysis showed that the treatment (ARV and/or CRISPR) was responsible for the new mutations/indels over the course of study. Using Stanford drug-resistance database and IAS Drug Resistance Mutation List, accessory mutations not belonging to any major drug-resistant mutations were found utilizing SGS, while both major and accessory mutations were detected in samples from ART and dual treatment groups using NGS deep sequencing suggesting drug resistance-mediated escape mutants may have contributed to the viral rebound. Additional mechanisms which may have contributed to the viral rebound remain under investigation.

### Bill Narayan Memorial Lecture

#### 21. HIV-1 ASSOCIATED NEUROCOGNITIVE DISORDERS (HAND) FOLLOWING CHRONIC MICROGLIAL INFECTION AND VIRAL PROTEIN PRODUCTION

Booze, R.M., Ph.D.^1^, Li, H., MD, Ph.D.^1^, McLaurin, K.A., MS^1^, Illenberger, J.M., MS^1^, Mactutus, C.F., Ph.D.^1^

^1^Department of Psychology, University of South Carolina, Columbia, SC 29208.

The persistence of HIV-1 viral reservoirs in the brain, despite treatment with combination antiretroviral therapy (cART), is a critical roadblock for the development of novel therapeutic and/or cure strategies. To enhance our understanding of viral reservoirs, two complementary studies were conducted to 1) evaluate the HIV-1 mRNA neuroanatomical distribution pattern and major cell type expressing HIV-1 mRNA in the HIV-1 transgenic (Tg) rat, and 2) to validate our findings by developing and critically testing a novel model of HIV-1 infection in the rat. First, a restricted HIV-1 mRNA distribution pattern was observed in the HIV-1 Tg rat; with microglia as the most abundant cell type expressing HIV-1 mRNA. Second, we developed and critically tested a novel biological system to model HIV-1 infection by infusing F344/N control rats with chimeric HIV (EcoHIV). *In vitro*, EcoHIV was applied to primary cultured microglia revealing prominent expression within 24 h post-infection. *In vivo*, expression of EcoHIV was observed seven days after stereotaxic injections in the brain. Consistent with results in the HIV-1 Tg rat, microglia were the major cell type expressing HIV-1 mRNA. Within eight weeks of infection, EcoHIV animals exhibited neurocognitive impairments, neuroinflammation, and synaptic dysfunction. Collectively, EcoHIV infection in rats replicates observations in the HIV-1 Tg rat, enhancing our understanding of HIV-1 viral reservoirs in the brain and offering a novel biological system to model HAND and associated comorbidities (i.e., drug abuse). Supported by NIH DA013137, HD043680, MH106392, NS100624.

### Addiction Medication Plenary Lecture

#### 22. ALCOHOL USE DISORDERS AND POTENTIAL NEURO-IMMUNE TREATMENTS

Mason, B.J., Ph.D.^1^

^1^Pearson Center for Alcohol and Addiction Research, Department of Molecular Medicine, Scripps Research, La Jolla, CA, 92037, USA

The prevalence of alcohol use disorder (AUD), and related mortality and morbidity, make effective treatment of global public health significance. Four medications have widespread regulatory approval for treatment of AUD: oral disulfiram, oral and injectable naltrexone, oral nalmefene and oral acamprosate. WHO analyses underscore the importance of the incremental gain that is achieved when medication is used in conjunction with counseling for AUD. However, utilization of AUD treatment is generally low, with a nationwide survey in the United States finding <2 % of afflicted persons treated with a medication for AUD. A new drug target is the reduction in neuroinflammation that accompanies chronic heavy alcohol use and that is associated with heightened drinking relapse risk in early abstinence. For proof of concept, we studied apremilast, a phosphodiasterase type 4 (PDE4) inhibitor that acts on immune system targets and is FDA-approved for the treatment of psiorasis. 50 volunteers with severe AUD were randomized to 2 weeks of double-blind treatment with apremilast (90 mg/d) or matched placebo. Apremilast significantly reduced the number of drinks per day (p=0.025) and the probability of a heavy drinking day (p=0.03) relative to placebo. Apremilast was safe and well-tolerated in this AUD outpatient sample. There were no serious, severe or unexpected adverse events. Effects of apremilast in decreasing drinking provide clinical validation of results obtained in animal models and lend support to neuro-immune modulation as an AUD treatment strategy.

### US-Indo Plenary Lecture 1

#### 23. ALCOHOL AND STRESS-IMMUNE AXIS: IMPACT ON ALCOHOL RELAPSE AND PHARMACOTHERAPY OUTCOMES

Sinha, R., Ph.D.^1^

^1^Psychiatry and Yale Stress Center, Yale University School of Medicine, New Hven, CT 06519.

Alcohol activates the stress axis, and powerfully modulates the stress response. Chronic alcohol use modifies the stress axis, significantly modifying subjective, neuroendocrine as well as peripheral immune responses and increasing alcohol craving and compulsive alcohol seeking. This presentation will demonstrate chronic alcohol-related adaptations in stress physiology, functional and structural brain changes and peripheral immune responses. Tonic and phasic changes in autonomic and hypothalamic-pituitary-adrenal (HPA) axis responses in binge drinkers and patients with alcohol use disorder (AUD) relative to light social drinkers will be shown. In addition, lower activity of cytokines, including IL6, Il10, I Il1-beta and TNF-alpha with specific differential effects of depression versus AUD will be shown. Functional brain responses in striatal-limbic and prefrontal cortical regions related to chronic alcohol intake and peripheral immune changes will also be discussed. Results on immune, neural, endocrine and increased craving effects on compulsive alcohol intake and in AUD relapse will be presented. Finally, data showing that a drug such as Prazosin is effective only in AUD patients who also have alcohol withdrawal by significantly reducing alcohol intake, cravings, anxiety as well as normalize the peripheral stress axis compared to those with withdrawal symptoms on Placebo. Together, the presentation will support the development of therapeutic targets that normalize the stress-immune axis in the treatment of chronic alcohol use disorder.Supported by National Institute of Alcohol Abuse and Alcoholism – USA.

### Symposium 2: Substance Abuse, HIV Infection and Associated Disorders

#### 24. SUBSTANCE ABUSE AND HIV ASSOCIATED PULMONARY VASCULAR DISEASE

Dhillon, N. ^1^

^1^Internal Medicine, University of Kansas Medicla Center, Kansas City, KS 66160.

The increased survival of HIV-infected individuals on anti-retroviral therapy (ART) is accompanied with an increased prevalence of various age-related non-infectious complications such as chronic obstructive pulmonary disease (COPD) and pulmonary hypertension. Lately there is increasing appreciation that the pulmonary vascular injury is not only associated pulmonary arterial hypertension but also with the development of emphysema and COPD related co-morbidities. Findings from our group has consistently suggested augmentation of pulmonary vascular remodeling in HIV infected intravenous drug users (IDUs) compared to HIV-infected non-drug users or un-infected IDUs. We demonstrated enhanced pulmonary arteriopathy in the lung sections from simian immunodeficiency virus – infected macaques and HIV – transgenic rats in the presence or absence of opioid abuse and this was associated with the enhanced apoptosis followed by increased proliferation of pulmonary endothelial cells. Furthermore, oxidative stress mediated autophagy was observed to be involved in the switching of these pulmonary endothelial cells from early apoptotic to late hyper proliferative state. Our recent data suggest NADPH oxidases as one of the main players in this oxidative stress mediated endothelial dysfunction. In conclusion, opioid exposure increases the severity of angio-proliferative remodeling of the pulmonary vasculature and thereby increasing the risk of developing HIV-related pulmonary co-morbidities. Supported by R01HL129875, R01DA034542 and R03DA031589; and 11SDG7500016 (AHA).

#### 25. ETHAMPHETAMINE POTENTIATION OF SARS-COV-2 N-PROTEIN-INDUCED NEUROINFLAMMATION VIA NLRP3 INFLAMMASOME ACTIVATION IN RAT MICROGLIA

Dutta, D., Ph.D.^1^, Liu, J., MD, Ph.D.^1^, Xiong, H., MD, Ph.D.

^1^Department of Pharmacology and experimental Neuroscience, University of Nebraska Medical Center, Omaha, NE 68198.

The SARS-CoV-2 infection causes immune-mediated neurological syndrome which persists long after infection. Mechanisms for SARS-CoV-2-associated neurological complications are multifactorial with increased risk in methamphetamine (Meth) users. Recent studies showed that SARS-CoV-2 proteins play a pivotal role in COVID-19-associated neuroinflammation. We hypothesize that the SARS-CoV-2 nucleocapsid (N), a highly immunogenic viral protein, induces microglial NLRP3 activation and sequelae in neuroinflammation, which is further exacerbated by Meth. To test this hypothesis, we investigated N-protein and Meth on NLRP3 inflammasome activation in primary rat microglial cultures using ELISA, RT-qPCR, WB and IFA. Our results showed that Meth potentiated N-protein-induced microglial activation as detected by Iba-1 expression. Addition of Meth to the microglial cultures treated with N-protein augmented proinflammatory cytokine production. Meth potentiation of N-protein-induced neuroinflammation was also detected by increased iNOS-mediated NO production. The Meth potentiation of N-protein-associated inflammatory responses was significantly attenuated by a specific NLRP3 inhibitor MCC950. Moreover, the Meth-associated NLRP3 activation was blocked by a specific sigma-1 receptor (σ1R) inhibitor BD1047 or by siRNA knockdown of σ1R. Taken together, our results demonstrated that Meth potentiated N-protein-induced neuroinflammation via σ1R and NLRP3 activation in microglial cells, which may underlie the pathogenesis of neurological syndromes in COVID-19 patients with Meth abuse. Supported by NIH grant R01DA050540

#### 26. DRUGS OF ABUSE AND SIV RESERVOIRS

Byrareddy, S., Ph.D.^1^

^1^Department of Pharmacology & Experimental Neuroscience, University of Nebraska Medical Center, Omaha, NE 68132.

A persistent Human Immunodeficiency Virus (HIV) reservoir during combined antiretroviral therapy (cART) is a significant barrier to curing HIV. The effect of opioids on viral reservoirs remains elusive. HIV infection causes rapid depletion of CD4+ T cells, and cART allows significant immune reconstitution. However, critical HIV-associated pathologies, including HIV-associated neurocognitive disorders (HAND), remain prevalent even in the cART era. More recent animal models of HIV pathogenesis suggest that significant gut damage and dysregulation contribute to ongoing peripheral immune activation even during cART.

Further, Opioids modulate the immune system and suppress antiviral gene responses, significantly impacting people living with HIV (PLWH). Therefore, treating opioid use disorder (OUD) and inflammation and maintaining patient well-being could substantially improve the quality of life, adherence to ART therapy, and overall clinical outcome. Further, controlling opioid use and understanding viral reservoir dynamics remain the knowledge gap in current HIV cure strategies. I will discuss some of our new data using rhesus macaques as models to address some of these shortcomings in the field of HIV cure research.

### Microbiome Workshop: The microbiota modulation of the neuronal-immune system

#### 27. FECAL MICROBIOME TRANSPLANT FROM ALCOHOLIC HEPATITIS (AH) PATIENTS INDUCES INFLAMMATORY CHANGES IN GUT-BRAIN-IMMUNE AXIS IN MICE

Barve, Shirish, Ph.D.^1^, Charpentier, B., MS^1^, Myers, S., Ph.D.^1^, Whittemore, S., Ph.D.^1^

^1^School of Medicine, University of Louisville, Louisville, KY 40202.

A substantial body of pre-clinical and clinical data indicates that chronic alcohol abuse causes induction of both peripheral and neuroinflammation leading to neurodegeneration. However, the mechanisms underlying the ethanol-induced development of neuro pathologic changes are not completely deciphered. The present study examined the potential causal role of fecal microbiota transplantation (FMT) from alcoholic hepatitis (AH) patients in mice (FMT-mice) in the induction of inflammatory changes in the Gut-Brain-Immune axis. FMT-mice showed reduction in the tight junction protein Claudin-5 and accumulation of F(ab’) fragments in the brain, indicative of blood brain barrier (BBB) dysfunction and increased permeability. These changes were accompanied by an increase in neutrophil infiltration and astroglial activation, marked by significant changes in GFAP and IBA-1 staining. Notably, along with neuro-inflammatory changes, FMT-mice also showed development of neuronal endoplasmic reticulum (ER) stress as demonstrated by an increase in KDEL peptide sequence and CHOP expression in Purkinje neurons in the cerebellum. In contrast, the FMT-mice showed a significant decrease in IL-10 positive immune cells that were further reduced upon alcohol feeding. These data strongly support a causal role for the alcohol-induced gut microbial dysbiosis, in the development of inflammatory changes in the gut-brain-immune axis in mice. These data also suggest that gut dysbiosis is a potential therapeutic target for the treatment of neuroinflammation in AH patients.

Supported by NIH/NIAAA.

#### 28. PRESCRIPTION OPIOID INDUCES CHANGES IN INTESTINAL MICROBIOME AND WORSEN INFLAMMATORY BOWEL DISEASE

Sharma, U., Ph.D. ^1^, Olson, R., MD^2^, Erhart, F., BS^1^, Sharma, M., MS^1^, Ramakrishnan, S., Ph.D.^1^, Abreu, M., MD^1^, Roy, S., Ph.D.^1^

^1^Miller School of Medicine, University of Miami, Miami, FL 33136

^2^Department of Surgery, University of Minnesota, Minneapolis, MN 55455.

Opioids are the most prescribed analgesics for pain in Inflammatory Bowel Diseases (IBD), however the consequences of opioid use on IBD severity is not well defined. This is the first study investigating consequences of hydromorphone in both dextran sodium sulfate (DSS)-induced colitis and spontaneous colitis (IL-10KO) mouse model of IBD. Wild-type (WT) mice were treated with clinically relevant dose of hydromorphone and colitis was induced via 3 % DSS in drinking water for 5 days. Hydromorphone and DSS independently induced barrier dysfunction, bacterial translocation, disruption of tight junction organization, and increased intestinal and systemic inflammation, which were exacerbated in mice receiving hydromorphone in combination with DSS. Hydromorphone plus DSS treated mice exhibited significant microbial dysbiosis. Predictive metagenomic analysis of the gut microbiota revealed high abundance in the bacterial communities associated with virulence, antibiotic resistance, toxin production and inflammatory properties. Hydromorphone modulates tight junction organization in a myosin light chain kinase (MLCK)-dependent manner. Treatment with ML-7 ameliorates the detrimental effects of hydromorphone on DSS induced colitis, thus decrease severity of IBD. Similarly, we demonstrated that hydromorphone treatment in IL-10KO mice resulted in accelerated clinical manifestations of colitis compared to control mice. Opioid use accelerates IBD progression by dysregulation of the gut microbiota leading to expansion of pathogenic bacteria, immune activation, and sustained inflammation. Supported by R01 DA043252, R01 DA037843, K05 DA033881, R01 DA044582, R01 DA034582 to S.R and GR010993 to U.S.

### Nanotechnology Plenary Lecture

#### 29. GETTING IN TO THE BRAIN: POTENTIAL OF NANOTECHNOLOGY TO CURE DRUG ADDICTIONS IN THE CONTEXT OF AIDS

Nair, M., Ph.D.^1^

^1^Department of Immunology and NanoMedicine, Herbert Wertheim College of Medicine, Florida International University, Miami, FL 33199

^2^Institute of NeuroImmune Pharmacology, Institute of NeuroImmune Pharmacology, Florida International University, Miami, FL 33199.

2018 report suggests that more than 260 million people are affected with various kinds of drug addictions and 37 million people are living with HIV/AIDS in the world today. Reports also show that more than 3–4 million people are co-affected with HIV and illicit drug use. Although highly active anti-retroviral therapy (HAART) has resulted in remarkable decline in the morbidity and mortality in AIDS patients, inadequate delivery of HIV drugs across the blood-brain barrier (BBB) to the brain results in HIV persistence. Drugs of abuse such as opiates act synergistically with HIV-1 to potentiate the HIV-related neurotoxicity that leads to development of Neuro-AIDS. In recent years, use of nanotechnology has shown exciting prospect for development of novel drug delivery systems. We herein report the development of a Magneto-Electric Nanocarrier (MEN) to deliver and release on demand of HIV drugs and opiate antagonist which are otherwise impenetrable to brain and inhibit HIV and reverse opiate mediated adverse neurological effects. The proposed nanocarrier is anticipated to simultaneously reduce Neuro-AIDS and opiate addiction in HIV-1 infected opiate addicts. Further, this invented/patented new technology will have universal applicability for targeting and controlled release of drugs against a variety of other CNS diseases such as Parkinson’s, Alzheimer’s, brain tumors etc. Supported by NIH grants.

### Symposium 3: Extracellular Vesicles in Substance Abuse and Neurological Disorders

#### 30. BRAIN ENDOTHELIAL CELL (EC) MICROVESICLE RELEASE DURING HIV INFECTION

Hale, J.F., MS ^1^, Potula, R., Ph.D.^1^, Ramirez, S.H., Ph.D.^1^, Andrews, A.M., Ph.D.^1^

^1^Pathology and Laboratory Medicine, Lewis Katz School of Medicine, Philadelphia, PA 19140.

Treatment of HIV-infected patients with antiretroviral therapy (ART) has effectively suppressed viral replication; however, the CNS is still a major target and reservoir of the virus leading to the possible development of HIV-1-associated neurocognitive disorders (HAND). Furthermore, a hallmark feature of HAND is the disruption of the blood-brain barrier that leads to loss of tight junction protein (TJP) complexes. Extracellular microvesicles (EVs), released by every cell type in the body, occur in greater quantities in response to cellular activation or injury. We have found that inflammatory insults activate brain endothelial cells (EC) and induce the release of EVs containing TJPs such as Occludin. We thus hypothesized that HIV infection and unresolved neuroinflammation will result in the release of brain-EC derived EVs. Herein, our results show elevated levels of brain-EC EVs in a humanized mouse model of HIV infection. Furthermore, while ART reduced brain-EC EVs, it was unable to completely resolve increased vesicles detectable in the blood. In addition to inflammatory insults, HIV-1 viral proteins (Tat and gp120) increased the release of Occludin + vesicles from human brain microvasculature ECs. This increase in vesicle release could be prevented by knock-down of the small GTPase ARF6. ARF6 has been shown to regulate EV biogenesis in other cell types, and we provide further evidence for the involvement of ARF6 in brain EC derived EVs. Overall, this study offers insight into the process of brain vascular remodeling in the setting of neuroinflammation. Supported by NIH/NIDA.

#### 31. ROLE OF EXTRACELLULAR VESICLES IN HIV/DRUG ABUSE INDUCED INFLAMMATION

Yelamanchili, S.V., Ph.D. ^1^

^1^Department of Anesthesiology, University of Nebraska Medical Center, Omaha, NE 68198.

Methamphetamine (MA) and related amphetamine compounds, which are potent psychostimulants, are among the most commonly used illicit drugs. With greater than 35 million users worldwide, MA abuse poses a significant health and economic threat globally. Chronic use of the drug is known to cause serious health problems leading to intense behavior changes including paranoia, insomnia, agitation, hallucinations and delusions. Neuroimaging studies have revealed that chronic MA abuse can indeed cause neurodegenerative changes in the brains of human MA abusers including prominent microglial activation throughout the brain. It is still unclear how chronic inflammation caused by MA abuse leads to long-term damage to the brain. Moreover, the specific role of non-neuronal cells such as glial cells in directly affecting neuronal heath is not well understood. With this in mind, we are particularly interested in studying the role of extracellular vesicles (EVs) in eliciting chronic inflammation in MA exposed brains. In the present study, we focus on the role of a miRNA, miR-29a-3p (miR-29a) in chronic MA exposure. Here, we present novel data that shows for the first time how chronic MA impacts not only the biogenesis but also the EV associated miRNA cargo thereby affecting the overall health of the neurons and glial cells in the brain. Supported by R01DA042379, R01DA046852.

#### 32. MOLECULAR AND CELLULAR EFFECTS OF MORPHINE AND NEF-EVS ON ALTERNATIVE SPLICING OF OPRM1 PRE-MRNA SPLICING

Sariyer, I.K., Ph.D.^1^, Yarandi, S., MS^1^, Huang, W., BS^2^, Donadoni, M., Ph.D.^1^, Chang, S.L., Ph.D.^2^

^1^Temple University, Lewis Katz School of Medicine, Philadelphia, PA 19140

^2^Seton Hall University, institute of Neuroimmune Pharmacology and Department of Biological Sciences, South Orange, NJ 07079.

Opioids, such as morphine, activate OPRM1, a member of the G protein-coupled receptor (GPCR) family. Studies on opioid receptor genes showed that the OPRM1 gene undergoes extensive alternative splicing events. To date, 21 isoforms of the human OPRM1 have been identified. However, characterization of OPRM1 signaling is usually generalized, and only few isoforms have been extensively studied. Compounding this issue is the increasing significance of intravenous drug abuse in HIV neuropathogenesis. Multiple studies have shown that opioid abuse may potentiate HIV neurocognitive alterations indirectly or directly by stimulating overlapping signaling pathways in neurons. We recently reported that glial cells infected with HIV-1 release Nef protein in extracellular vesicles (Nef-EV). Interestingly, Nef-EVs exert same effects as those with morphine on alternative splicing of OPRM1 pre-mRNA by inducing the alternative splicing of MOR-1X isoform. More interestingly, combined treatment with Nef-EVs and morphine has a synergistic effect on alternative splicing of MOR-1X isoform in both *in vitro* and *in vivo* settings. Furthermore, the impact of morphine on expression of key genes involved in opioid dependence was modulated by Nef-EVs in neuronal cells. Moreover, alternative splicing of MOR-1X isoform was reversed by anti-sense oligonucleotides delivered by exosomes targeting ISE/ISE elements on OPRM1 pre-mRNA and CRISPR/Cas gene editing targeting exon X. Our results suggest that HIV-1 may regulate the rate of opioid dependence in HIV/AIDS by amplifying the rate morphine effects. Supported by P30MH092177, R21 AA025398, DA046258.

#### 33. EXOSOMAL EXTRACELLULAR VESICLES WITH NEF ARE BIOSIGNATURES OF HIV-ASSOCIATED NEUROCOGNITIVE IMPAIRMENT STATUS

Raymond, A., Ph.D.^1^, Caobi, A., Ph.D.^1^, Werne, R., MD^1^, Gomez, M., Ph.D.^2^, Andre, M., BS^1^, Yndart, A., BS^1^, Lima-Hernandez, F., Ph.D.^2^, Kolber, M., MD^3^, Nair, M., Ph.D.^1^

^1^Department of Immunology and Nanomedicine, Herbert Wertheim College of Medicine at Florida International University, Miami, FL 33199

^2^College of Arts, Sciences, and Education, Florida International University, Miami, FL 33199

^3^Miller School of Medicine, University of Miami, Miami, FL 33136.

Aviremic people living with HIV (PLWH) have exosomal extracellular vesicles (xEVs) in the serum and cerebrospinal fluid (CSF) containing the HIV Negative factor (Nef) protein. The role xEV-Nef plays in HIV infection is unknown. Here we have performed a cross-sectional study examining the content of matched serum- and CSF-derived xEVs and Nef-containing-xEVs of PLWHs with the neurocognitive status of PLWHs. The study objective was to define a biosignature of HIV-associated neurocognitive impairment (NCI). The xEV concentration in CSF is higher than in serum, and when stratified by NCI, the CSF of PLWH with NCI has significantly more xEVs than the CSF of participants without NCI. Nef-xEVs were elevated in NCI compared to the non-NCI group. CSF xEV-Nef levels directly correlated with CD4 counts, but serum xEV-Nef did have any correlation with CD4 counts. The ratio of xEV-Nef to CD4 counts in the CSF demonstrates that NCI status can be associated with xEV-Nef concentration and CD4 ratio. Within this small group of PLWHs, we identified a biosignature based on the potential protein expression profile within serum-derived exosomes correlating with NCI status. This finding suggests that peripheral xEVs can indicate neuropathology in PLWHs and that xEVs could be a new paradigm to confirm neurocognitive impairments. Supported by National Institute of Health/NIH R01-DA044498-01; Center for AIDS Research Pilot grant; HWCOM pilot grant.

#### 34. NEUROPATHOGENIC ROLE OF ASTROCYTE-DERIVED EXTRACELLULAR VESICLES IN HIV ASSOCIATED NEUROCOGNITIVE DISORDERS

Sil, S.S., Ph.D. ^1^, Chemparathy, D.T.C., Ph.D.^1^, Ochs, C.O., Ph.D.^1^, Ferguson, N.F., Ph.D.^1^, Callen, S.C., MS^1^, Buch, S.B., Ph.D.^1^

^1^Pharmacology & Experimental Neuroscience, University of Nebraska Medical Center, Omaha, NE 68198.

Although cART usage has increased the lifespan of HIV + individuals, paradoxically, its dependence is also associated with increased risk of Alzheimer’s-like pathology, as a comorbidity of HIV-Associated Neurocognitive Disorders (HAND). Our previous findings showed that astrocytes play a major role in HIV-1 Tat-mediated amyloidosis, & since amyloids can be released in extracellular vesicles, we sought to assess whether HIV-1 Tat stimulated astrocyte derived EVs (ADEVs) containing toxic amyloids could lead to neuronal injury *invitro* & synaptodegeneration & cognitive impairments when administered in the brains of naive mice. Our previous studies have demonstrated the role of HIF-1α as an upstream regulator of Tat mediated astrocytic amyloidosis. We thus hypothesized that blocking HIF-1α could likely mitigate neuronal injury. Rat hippocampal neurons exposed to Tat-ADEVs carrying amyloids resulted in reduction of dendritic arborization, spines, synaptic proteins & post-synaptic currents. Silencing of astrocytic HIF-1α reduced the release of ADEVs & their amyloid cargos, & also ameliorated neuronal injury. Next, Tat-ADEVs carrying amyloids when injected in the hippocampus of naive mice brains, resulted in cognitive impairment(s), as well as amyloid deposition in the neurons & astrocytes resulting in synaptodegeneration. This impairment(s) was not seen when injected with HIF-1α silenced ADEVs. This study underscores the role of amyloid carrying ADEVs in mediating synaptodegeneration leading to cognitive impairments associated with HAND & highlights the protective role of HIF-1α. Supported by R25MH080661-John Hopkins University (S. Sil) and R21AG069541 (S. Sil) from National Institute of Health.

### Symposium 4: Drug Abuse, Alcohol and Medication

#### 35. EFFECTS OF CANNABIDIOL ON COCAINE SEEKING: DOSE-RESPONSE PROFILES AND NEUROBIOLOGICAL SUBSTRATES

Weiss, F., Ph.D.^1^, Nedelescu, H., Ph.D.^1^, Wagner, G.E., BS^1^, Suto, N., Ph.D.^1^

^1^Department of Neuroscience, Scripps Research, La Jolla, CA 92037.

Cannabidiol (CBD) has received attention for potential in drug relapse prevention. In animal models CBD attenuates reinstatement of cocaine, alcohol, and heroin seeking as well as morphine- and cocaine-conditioned place preference (CPP). In our previous studies, CBD reduced cocaine and alcohol seeking across a 7-day treatment period. However, maximal effects of the single CBD dose in these studies occurred well before CBD reached maximal brain levels. Here, we follow up on these findings by establishing the dose-response (D-R) profile and neurobiological substrates for CBD-induced suppression of drug seeking using a cocaine CPP model. Following acquisition of CPP, rats received one of six CBD doses daily and then were tested for expression of CPP. CBD produced linear increases in CBD brain/plasma concentrations across doses but suppressed CPP in a distinct U-shaped manner, with efficacy at intermediate doses and lack of effects at the highest dose. Neural activation in brain of rats treated with the most effective CPP suppressant dose was reduced in the prelimbic but not infralimbic mPFC or nucleus accumbens core and shell. RNAscope^®^ revealed reduced activation of prelimbic glutamatergic neurons by the preferred cocaine context as the predominant effect of CBD and, thus, as a potential mechanism for CBD’s “anti-relapse” actions. Overall, the findings confirm anti-relapse potential for CBD. However, the D-R profile of CBD may have important implications for clinical treatment regimens and understanding of the conflicting literature concerning CBD effects on drug seeking. Supported by U.S. National Institutes of Health (NIH): AA022082, DA039821 (FW); DA043030 (NS).

#### 36. THERAPEUTIC USE OF KETAMINE IN PSYCHIATRIC DISORDERS

Chen, H.H., Ph.D.^1^

^1^Center For Neuropsychiatric Research, National Health Research Institutes, Zhunan, 35053.

Ketamine is a dissociative anesthetic agent that at differing doses can be used as an analgesic, sedative, anesthetic induction and anesthetic maintenance agent. Recently, the remarkable rapid-onset antidepressant effects of ketamine have been shown in patients with treatment-resistant depression. Although esketamine, one of ketamine enantiomer, nasal spray has been approved for treatment-resistant depression by the FDA, the psychiatric, psychotomimetic, and neurocognitive side effects and addictive potential of ketamine are still a concern. Therefore, we focused on the development of adjunctive therapy which can promote therapeutic efficacy and concomitantly avoid the adverse effects of ketamine for treatment-resistant depression. Based on the NMDAR is the critical target for ketamine-induced psychosis, the acute and subchronic combined effects of ketamine and the modulators of NMDAR glycine binding site on depression-like, psychosis-like, cognitive function, chronic pain, anesthesia, and reinforcing efficacy were assessed. Two potential compounds as add-on therapy to ketamine for treatment-resistant depression and chronic pain have been identified. Supported by MOST Taiwan.

#### 37. ARIJUANA: POTENTIAL TREATMENT OF NEUROPSYCHIATRIC DISORDERS

Onaivi, E.S., Ph.D.^1^, Canseco-Alba, A., Ph.D.^1^, Sanabria, B.D, MS^1^, Patel, P., BS^1^, Ishiguro, H., MD, Ph.D.^2^, Horiuchi, Y., Ph.D.^3^, Ali, S.F, Ph.D.^4^, Liu, Q.R., Ph.D.^5^

^1^Department of Biology, William Paterson University, Wayne, NJ 07470

^2^Department of Psychiatry, University of Yamanashi, Yamanashi, 409-3898

^3^Medical Science, Tokyo Metropolitan Institute, Tokyo, 156-8506

^4^National Center for Toxicology, FDA, Jefferson, AR 72079

^5^NIA, NIH, Baltimore, MD 21224.

Neuroinflammation is emerging as a key component in the effects of CB2Rs expressed in neurons and glial cells that are key regulators of immune response. We generated Cx3Cr1-Cnr2 cKO mice with selective deletion of CB2Rs in microglia. We utilized multidisciplinary approaches to determine the neuro-immuno-modulatory effects of CB2Rs in the DAT-Cnr2, Cx3Cr1-Cnr2 cKO and wild type (WT) C57BL/6J mice. Here we report (1). That CB2Rs are involved in the tetrad assay induced by cannabinoids in the WT and the CB2R cKO mice contrary to the long-standing notion that the characteristic tetrad tests were induced mainly by CB1R agonism. (2). In the hippocampus, there was enhanced IBA1 immunoreactivity in both CB2R cKO mice, and microglia activation was detected by CD11b in the dentate gyrus in WT, DAT-Cnr2 and Cx3cr1-Cnr2 mice with clear morphological difference in the Cx3cr1-Cnr2 mice after stress. (3). Neuroinflammation signaling pathways of PI3K/AKT/mTOR, MAP/ERK and NF-KB were differentially affected by the cell-type specific deletion of CB2R in cerebral cortexes of CB2R cKO and WT mice. (4). Alcohol preference ratio was significantly higher in Cx3cr1-Cnr2 cKO and WT, than DAT-Cnr2 cKO mice that consumed less alcohol. WT mice and Cx3cr1-Cnr2, but not DAT-Cnr2 cKO mice showed robust conditioning to alcohol in the CPP paradigm. We concluded that microglia and DA neuron specific deletion of CB2Rs reveals a neuro-immune basis for the behavioral alterations, and modulation of alcohol behavioral effects of type 2 cannabinoid receptors.

#### 38. SEXUAL DIMORPHISM IN THE EFFECT OF COMMONLY USED ANESTHETICS ON THE VASOACTIVE PROPERTIES OF ALCOHOL AND TETRAHYDROCANNABINOL IN THE BRAIN

Bukiya, A.B., Ph.D.^1^, Steven Mysiewicz, S., BS^1^, Dopico, A.M., MD, Ph.D.^1^

^1^Department of Pharmacology, Addiction Science and Toxicology, The University of Tennessee Health Science Center, Memphis, TN 38163.

Alcohol and cannabis are among the most widely used psychoactive substance in the world, each possessing distinct effects on brain function. Due to the importance of the cerebral circulation in brain homeostasis, a growing number of studies address the effects of alcohol and cannabis on the cerebral circulation. We set to address the influence of commonly used anesthetics on the effect of alcohol and delta-9-tetrahydrocannabinol (THC) on cerebral arteriole diameter. Sprague–Dawley rats were subjected to intracarotid catheterization and cranial window surgery for monitoring pial arteriole diameter *in vivo* in response to drug infusion under ketamine versus isoflurane anesthesia. We focused on pial arterioles stemming from the middle cerebral artery since this artery supplies blood to the largest area of the brain. Alcohol and THC were probed at toxicologically relevant concentrations. In males, alcohol constricted pial arterioles under ketamine but not isoflurane anesthesia. In females, alcohol did not modify vessel diameter regardless of anesthetic. In both males and females under ketamine, THC dilated arterioles while constricting arterioles in females under isoflurane. Our data point at sex-dimorphic control of anesthetics over alcohol and THC effect on cerebral arteriole diameter. Such control is expected to modify brain perfusion and thus, should be known to anesthesiologists handling patients under influence of drugs and to medical researchers who perform bench studies on the subject.

### Adarsh Kumar Memorial Lecture

#### 39. CEREBROVASCULAR PATHOLOGY OF HIV INFECTION AND DRUG ABUSE

Toborek, M., MD, Ph.D.^1^

^1^Department of Biochemistry and Molecular Biology, University of Miami Miller School of Medicine, Miami, FL 33136.

The blood-brain barrier (BBB) is a critical structural component that protects the brain from pathogens, maintains cerebral homeostasis, and regulates the exchange of molecules between the blood and the CNS. Infection with HIV is an epidemic affecting nearly 38 million people worldwide in which the virus attacks and infects the body’s immune cells, ultimately compromising the integrity of the BBB and CNS as well. The breakdown of the BBB further potentiates viral replication within the CNS, which can lead to HIV-associated neuropathology. HIV-associated cerebrovascular events remain highly prevalent even in the current era of antiretroviral therapy. It appears that low-level HIV replication and associated inflammation endure despite antiretroviral treatment and affect cerebrovascular health, resulting in worsening ischemic stroke severity and outcomes. These processes are further be altered and potentiated by exposure to drugs of abuse, such as methamphetamine or opioids. Among the components of the BBB, pericytes can be a target of HIV-1 infection as they are able to support productive HIV-1 replication. In addition, BBB pericytes are prone to establish a latent infection. Indeed, recent evidence from animal and human studies indicates that BBB pericytes can be a previously unrecognized HIV-1 target and reservoir in the brain, warring more detailed research in the context drug abuse. Overall, this research provides important insight for treatment of HIV-infected patients who abuse drugs and who are at risk of developing cerebrovascular disease. Supported by the NIH (MH098891, MH072567, HL126559, DA039576, DA044579, DA040537, and DA047157).

### Symposium 5: Autophagy and Neuroinflammation: from Mechanism to Therapeutic Opportunities

#### 40. GUT IMMUNE AND MICROBIAL CHANGES IN RESPONSE TO BURN INJURY

Choudhry, M., Ph.D.^1^

^1^Burn & Shock Trauma Research Institute, Loyola University Chicago Health Sciences Division, Maywood, IL 60153-3328.

Sepsis and multiple organ dysfunction continue to be the leading causes of death in trauma patients as well as in patients admitted to the intensive care units. The management of these patients becomes much more difficult when these injuries are accompanied by prior alcohol consumption, however, the underlying mechanism remains largely unclear. Gut barrier dysfunction has been implicated in the development of sepsis and multiple organ failure in the injured host. Utilizing a mouse model of burn injury, studies in our laboratory have examined the effects of burn on gut barrier integrity, and determined whether the presence of ethanol at the time of injury modulates gut barrier integrity after burn injury. We observed that intoxication at the time of burn injury: (1) exacerbates the suppression of gut associated lymphoid Th17 effector functions as assessed by their cytokine (IL-17 and IL-22) production; (2) disrupts the normal gut microbiota; (3) enhances gut tissue damage and leakiness; and (4) increases gut bacterial translocation, within 24 h of injury. We further demonstrated that treatment of mice with IL-22, a cytokine released by Th17 cells, prevented the increase in gut leakiness following ethanol and burn injury. Taken together, our findings suggest the decrease in Th17 effector cytokines after injury can result in gut barrier disruption. Furthermore, our finding suggesting the role of IL-22 in protecting gut barrier will help in developing effective therapy for the treatment of this critically-ill patient population. Supported by R01AA015731, R01GM128242 and T32AA013527.

#### 41. COCAINE-MEDIATED MICROGLIAL ACTIVATION INVOLVES DYSREGULATED AUTO/MITOPHAGY

Buch, S., Ph.D.^1^, Thangaraj, A., Ph.D.^1^

^1^Department of Pharmacology and Experimental Neuroscience, University of Nebraska Medical Center, Omaha, NE 68198.

While cocaine abuse involving glial activation underlies neuroinflammation, the exact molecular mechanism(s) mediating microglial activation remain unknown. In this study, we sought to determine whether mitophagy played a role in cocaine-induced microglial activation. Our results demonstrated that exposure of mouse primary microglial cells to cocaine resulted in reduced mitochondrial membrane potential with a concomitant increase in microglial activation. Activation, in turn, was accompanied by an increase in the expression of mitophagy signaling proteins such as PINK1, PRKN, and DNM1L, leading to increased fragmentation of depolarized mitochondria, primarily for their clearance via mitophagy. Cocaine exposure also resulted in upregulated expression of the autophagosome markers – Beclin1 and LC3-II, thereby suggesting increased mitophagosome production. These findings were also corroborated by cellular imaging studies demonstrating the co-localization of damaged mitochondria with the LC3 puncta. Interestingly, cocaine exposure also led to increased expression of p62, thereby suggesting possible blockade of mitophagy flux with the resulting accumulation of mitophagosomes. Cocaine exposure to primary microglia resulted in increased expression of proinflammatory cytokines such as TNFα, IL1β, and IL6 – that are hallmark features of microglial activation. Pharmacological approaches to block either the sigma receptor or autophagy/mitophagy signaling further underscored the involvement of mitophagy activation with impaired autophagic clearance in the presence of cocaine. Supported by NIDA.

#### 42. BRAIN AND LUNG INJURY CAUSED BY ALCOHOL AND ELECTRONIC CIGARETTES: MECHANISMS OF DELETERIOUS EFFECTS ON BLOOD BRAIN AND ALVEOLAR - ENDOTHELIAL BARRIERS

Persidsky, Y., MD, Ph.D.^1^, Mekala, N., Ph.D.^1^, Gajghate, S., MS^1^, Rom, S., Ph.D.^1^, Sriram, U., Ph.D.^1^

^1^Department of Pathology Laboratory Medicine, Lewis Katz School of Medicine, Temple University, Philadelphia, PA 19040.

Polydrug abuse (especially alcohol use disorder, AUD, and smoking) are known individually to compromise the lung alveolar-endothelial barrier (AEB) and the blood brain barrier (BBB). Very limited knowledge exists regarding damage in lung and brain due to electronic cigarettes (e-cig) in combination with AUD. While e-cig are known to be addictive, their effects on the brain and cognition are unknown. Chronic e-cig exposure in mice enhanced permeability of the BBB, increased neuroinflammation, diminished expression of a key glucose transporter and tight junction protein on brain endothelium, and impaired cognition. We discovered that the combination of alcohol/e-cig exposure in an animal model caused enhanced AEB permeability and signs of neuroinflammation/BBB compromise. We found that e-cig and alcohol cause mitochondrial dysfunction and oxidative stress, leading to pro-inflammatory phenotype of cellular components of AEB/BBB pointing to potential synergistic effects of e-cig/AUD on end-organ pathology. Using primary human brain endothelial cells, lung epithelial and endothelial cells, we found that alcohol and e-cig share similar mechanisms of injury. E-cig and alcohol caused mitochondrial impairment [spared respiration measured by Seahorse, diminished expression of Complex-II and Complex-IV and signs of endoplasmand signs of endoplasmic reticulum stress leading to intracellular Ca2+ accumulation and extracellular ATP releases. Further, we discovered a role of puronergic receptor P2X7 in these harmful effects of alcohol and e-cig suggesting novel therapeutic intervention. Supported by R37AA015913, U01AA023552.

#### 43. HIV TAT AND MORPHINE-MEDIATED ACTIVATION OF ASTROCYTES INVOLVES EPIGENETIC MODIFICATION OF THE NLRP6 INFLAMMASOME

Periyasamy, P., Ph.D.^1^, Buch, S., Ph.D.^1^, Kannan, M., Ph.D.^1^

^1^Department of Pharmacology and Experimental Neuroscience, University of Nebraska Medical Center, Omaha, NE 68198.

The Centers for Disease Control and Prevention describes HIV infection and drug abuse as intertwined epidemics, leading to compromised combine antiretroviral therapy adherence and exacerbation of HIV-associated neurocognitive disorders (HAND). Chronic low-level inflammation (mediated by viral proteins, antiretrovirals, and abused drugs) has been implicated as a significant underlying factor as well as an essential correlate of HAND pathogenesis. In this study, we hypothesized that exposure of mouse primary astrocytes to HIV Tat and morphine exacerbates astrocyte activation involving downregulation of miR-152, which in turn, targets NLRP6, leading to cleavage and release of IL1β and IL18. This ultimately culminates in neuroinflammation. MiRNA array analysis of HIV Tat and morphine exposed mouse primary astrocytes showed decreased levels of miR-152 with a concomitant upregulation of NLRP6 inflammasome and cellular activation. Gene silencing approaches further validated HIV Tat and morphine-mediated activation of NLRP6, cleavage of caspase1 and IL1β, and IL18 in mouse primary astrocytes. Overall, these findings underpin the epigenetic involvement of NLRP6 inflammasome signaling in astrocyte activation in the context of HIV Tat and morphine. Supported by NIDA/DA052266.

### ECITA Symposium

#### 44. ECOHIV INFECTED CHME5 AS A NOVEL IN VITRO MODEL FOR HIV INFECTION AND LATENCY IN MICROGLIA

Ostermeier, B., BS ^1^, Maggirwar, S.B., Ph.D.^1^

^1^Microbiology, Immunology, and Tropical Medicine, The George Washington University, Washington, DC 20052.

Human immunodeficiency virus (HIV), is a retrovirus that can infect multiple reservoirs in the body, including microglial cells within the central nervous system (CNS). HIV infection in microglia likely assumes a state of latency, in which HIV is transcriptionally silent in the host genome. Studying CNS resident cells *in vitro* can be difficult since patient samples are not readily available. There are multiple models for studying HIV infection and latency in microglia *in vitro*. Human induced pluripotent stem cell-derived microglia and primary microglia are great HIV infection models, but they are expensive and non-dividing, thus limiting sample pool sizes. While immortalized (human and mouse) and monocyte-derived microglia models are less expensive alternatives, they lack initial robust HIV infection due to the increased presence of SAMHD1, which is known to be inhibited by SIV-derived Vpx. In this study, we propose a novel *in vitro* immortalized microglial cell model of HIV infection, EcoHIV-infected CHME5 (rat) cells that are primed with the Vpx-containing virus-like particles (VLPs). We utilized flow cytometry, imaging, and western blots, to develop a model of both infection and latency. Latency was confirmed using a range of latency reversing agents (LRAs). Our novel HIV infection model has shown robust infection rates, latency, and reversal of latency. With this model, researchers can readily investigate new pathways that influence HIV infection and latency in microglia, which will inform future HIV treatment and cure strategies.

#### 45. DEVELOPMENT OF DRUG DELIVERY SYSTEM WITH ANTIRETROVIRAL AND NUTRACEUTICAL TO SUPPRESS HIV IN BRAIN RESERVOIRS

Godse, S., MS ^1^, Zhou, L., MS^1^, Sinha, N., BS^1^, Kumar, S., Ph.D.^1^

^1^Department of Pharmaceutical Sciences, University of Tennessee Health Science Centre, Memphis, TN 38163.

Our previous study has shown that poly (lactic-co-glycolic acid) (PLGA)-encapsulated antiretroviral drug increases the permeability and efficacy of the drug in an *in vitro* blood-brain-barrier (BBB) and *in vivo* animal models. We hypothesize that the use of a nutraceutical (NC01), which inhibits efflux transporters, can increase the concentration of an ART drug (ART01) across the BBB in brain cells and can subsequently suppress HIV in brain reservoirs. Since NC01 also has anti-inflammatory and antioxidant properties, hallmarks of HIV neuropathogenesis, the combination would further suppress HIV neuropathogenesis. Our results showed that the presence of NC01 increases the concentration of ART01 in both *in vitro* and *in vivo* systems. We also observed modulation of cytokine profile with significant decrease in proinflammatory cytokines and increase in anti-inflammatory cytokines after ART01 and NC01 treatment in U1 cells. Importantly, compared to intraperitoneal (IP) route of administration, intranasal (IN) administration of ART01 at two different concentrations (5 mg/kg and 25 mg/kg) further enhances the concentration of ART01 in brain and decreases its concentrations in plasma and peripheral organs. Further, we developed a PLGA nanoformulation with both ART01 and NC01 and characterized these for physicochemical parameters. We are in the process of determining biodistribution of PLGA-ART01/PLGA-NC01 formulations in plasma and different organs, especially brain, and their efficacy in suppressing viral load and amelioration of neuroinflammation in both *in vitro* BBB and animal models. Supported by NIH 1R21MH125670-01A1.

#### 46. HIV ASSOCIATED DYSBIOSIS INFLUENCES SELF-ADMINISTRATION OF COCAINE IN HIV-1 TRANSGENIC RATS

Rodriguez, M.T., MS ^1^, McLaurin, K.A., Ph.D.^1^, Mactutus, C.F., Ph.D.^1^, Booze, R.M., Ph.D.^1^

^1^Experimental Psychology, University of South Carolina, Columbia, SC 29208.

HIV-1 infection has been found to be associated with microbial translocation and dysbiosis. These changes are associated with the progression and severity of HIV associated neurocognitive disorder (HAND) symptoms in humans and are exacerbated in the presence of comorbidities such as substance use disorder and apathy. HIV-1 associated dysbiosis is characterized by an increase in the bacteria Prevotella and a reduction in Akkermansia Muciniphila, a combination that may modulate motivational behaviors through dysregulation of the dopaminergic system. The present study investigated the effect of S-equol (SE) at 0.2 mg on cocaine-maintained responding via modulation of the gut microbiome. The study included 42 female ovariectomized rats, 21 HIV-1 Transgenic rats (Tg) and 21 F344 control animals. A discriminant function analysis (DFA) and an ANCOVA was used to see if Prevotella and Akkermansia Muciniphila strains could be used to differentiate between genotypes and to determine if either strain varried with cocaine-maintained responding. Baseline measures of Prevotella_UCG_001 were found to significantly covary with lever presses for drug (p≤0.001), after treatment the change in Prevotella_UCG_001 was found to covary based on treatment and genotype (p≤0.035), suggesting an initial effect on motivation for cocaine that was altered by SE. The results suggest that SE may be a viable option for treating motivational dysfunction associated with substance use disorder in those living with HIV through targeting the gut-brain-microbiota axis. Supported by NIH 5T32GM081740, MH106292, DA013137, NS100624.

#### 47. IN VIVO HIV-1 TAT PROTEIN EXPRESSION ALTERS SYNAPTIC DOPAMINE RELEASE IN A REGION-SPECIFIC MANNER IN INDUCIBLE TAT TRANSGENIC MICE USING FAST SCAN CYCLIC VOLTAMMETRY

Davis, S.E., MS ^1^, Ferris, M.J., Ph.D.^2^, Ananthan, S., Ph.D.^3^, Augelli-Szafran, C., Ph.D.^3^, Zhu, J., MD, Ph.D.^1^

^1^Department of Drug Discovery and Biomedical Sciences, University of South Carolina, Columbia, SC 29203

^2^Department of Physiology and Pharmacology, School of Medicine, Wakeforest University, Winston-Salen, NC 27157

^3^Department of Chemistry, Southern Research Institute, Birmingham, AL 35205.

Dysregulation of dopaminergic transmission induced by the HIV-1 transactivator of transcription (Tat) has been implicated as a central factor in the development of HIV-1 associated neurocognitive disorders (HAND). Using Fast-Scan Cyclic Voltammetry (FSCV), this study determined whether Tat influences extracellular dopamine (DA) dynamics through regulation of DA transporter (DAT) and vesicular monoamine transporter2 (VMAT2). We found that 14-day doxycycline (Dox)-induced Tat expression in inducible Tat transgenic (iTat-tg) mice resulted in a two-fold increase in phasic but not tonic baseline DA release in the caudate putamen (CPu), whereas the baseline DA release was decreased (∼50 %) in the nucleus accumbens (NAc), compared to G-tg (Tat null) mice. A single systemic injection of a novel DAT allosteric modulator, SRI-32743, reversed the Tat-induced increase in baseline DA release in the CPu, while SRI-32743 alone did not alter baseline DA release. To determine whether Tat influences DA dynamics through VMAT2, we determined the effect of VMAT2 inhibitor, Ro4-1284, on Tat-mediated DA release in the NAc slices. No difference in bath-application of 5 µM Ro4-1284-depleted DA release was found between Dox-treated iTat-tg and G-tg mice; however, bath-application of d-amphetamine in the same slices evoked cytosolic DA release, which was increased in iTat-tg compared to G-tg mice. These findings suggest that Tat dysregulates DA homeostasis via inhibition of both DAT and VMAT2, which provide a new insight into the development of novel therapeutic strategies for the prevention of HAND. Supported by Supported by the SPARC grant from the University of South Carolina and NIH grant 1F31DA057163 to SD and NIH grants DA035714.

#### 48. HIV-1 INTEGRASE INHIBITOR ASSOCIATED PATHWAYS INVOLVED IN NEUROPSYCHIATRIC DISORDERS

Naik, B. ^1^

^1^Center for AIDS Health Disparities Research, KIIT University, Bhubaneswar, 751024.

Approximately 37 million people worldwide are living with HIV-1. In spite of considerable progress in HIV/AIDS research, anti-retroviral therapy (ART) remains the only treatment option for HIV-1 infection. Among the most widely prescribed antiretrovirals (ARVs) are integrase strand transfer inhibitors (INSTIs) which block the critical step of HIV-1 integration into host chromosome. Currently, it is recommended that INSTIs be included in all initial regimens for HIV. Although generally reported to be safe and effective there is growing concern about adverse metabolic and neuropsychiatric effects associated with the newer INSTIs. Therefore, understanding the mechanisms that drive neuropsychiatric effects of INSTIs are critically important for the long-term success of ART. To span this knowledge gap, we have been studying INSTI-associated adverse neuronal effects using both *in vitro* and *in vivo* models. In this study, we investigated the effects of two INSTIs; dolutegravir (DTG) and raltegravir (RAL) on neuronal function. Our results show that DTG exposure resulted in a marked reduction in neurite length, concurrent with a decrease in post-synaptic protein and an increase in glutamate levels. DTG exposure also dysregulated key genes of glutamate signaling, synaptic function, and calcium signaling. These DTG-induced neuronal alterations were not observed with RAL-another INSTI that is not associated with NPAEs. Collectively, these results identify key mechanisms underlying INSTI-mediated dysfunction in neuronal communication. Supported by 1R01DA057204-01, 2U54MD007586-36.

#### 49. CHARACTERIZING THE RESPONSE OF HCMEC/D3 CELLS TO THE SYNTHETIC CANNABINOID 2 RECEPTOR AGONIST, PM289

Bullock, T., MS^1^, Leonard, B.M., MS^1^, Hale, J.F., MS^1^, Morales, P., Ph.D.^4^, Jagerovic, N., Ph.D.^4^, Andrews, A.M., Ph.D.^1^, Ramirez, S.H., Ph.D.^1^

^1^Department of Pathology and Laboratory Medicine, Lewis Katz School of Medicine at Temple University, Philadelphia, PA 19140

^2^Center for Substance Abuse Research, Lewis Katz School of Medicine at Temple University, Philadelphia, PA 19140

^3^The Shriners Hospital Pediatric Research Center, Lewis Katz School of Medicine at Temple University, Philadelphia, PA 19140

^4^Instituto de Química Médica, Consejo Superior de Investigaciones Científicas, Madrid.

Discovered in the early 90s, the Cannabinoid 2 Receptor (CB2) was historically considered the immunological cannabinoid receptor. More recently, CB2 has been detected in the brain and cerebral vasculature leading to the investigation of this GPCR as a novel therapeutic target in numerous disease states. Of note, cerebrovascular CB2 is upregulated by inflammatory stimuli and can promote resolution of inflammation at the level of the blood brain barrier. However, the specific role of CB2 in cerebrovascular endothelial cells remains unclear. Here, the effect of the novel, chromenopyrazole based CB2 agonist, PM289, on hCMEC/D3 cells was investigated. Briefly, cells were treated with TNFa, TNFa + PM289 (10 nM–10 µM), or TNFa + PM289 (10 nM–10 µM) +SR144528 (10 uM). Angiogenic, physical barrier, and immunological barrier responses were considered in addition to the effect of PM289 on cell viability. The most prominent effects of PM289 were on measures of the physical and immunological barrier. Specifically, TNFa induced a 20 percent reduction to Transendothelial Electrical Resistance (physical barrier) and a 5-fold increase of ICAM expression (immunological barrier) compared to untreated cells. Both effects were partially abrogated by concurrent treatment with PM289 at nanomolar and low micromolar concentrations. Treatment with TNFa, PM289, SR144528, or combinations thereof did not affect cell viability. Ongoing investigation seeks to confirm these findings as CB2 mediated drug effects and interrogate endothelial cell signaling pathways which may be activated by CB2 agonists. Supported by R01 DA052970-02.

#### 50. INVOLVEMENT OF TRPM7 IN ALCOHOL-INDUCED DAMAGE OF THE BLOOD-BRAIN BARRIER IN THE PRESENCE OF HIV VIRAL PROTEINS

Mack, M.L., MS ^1^, Huang, W., MS^1^, Chang, S.L., Ph.D.^1^

^1^Institute of NeuroImmune Pharmacology, Seton Hall University, South Orange, NJ 07079.

Ethanol (EtOH) exerts its effects through various protein targets, including transient receptor potential melastatin 7 (TRPM7) channels, which play an essential role in cellular homeostasis. We demonstrated that TRPM7 is expressed in rat brain microvascular endothelial cells (rBMVECs), the major cellular component of the blood-brain barrier (BBB). Heavy alcohol drinking is often associated with HIV infection, however the mechanisms underlying alcoholinduced BBB damage and HIV proteins, are not fully understood. We utilized the HIV-1 transgenic (HIV-1Tg) rat to mimic HIV-1 patients on combination anti-retroviral therapy (cART) and demonstrated TRPM7 expression in rBMVECs is lower in adolescent HIV-1Tg rats compared to control animals, however control and HIV-1Tg rats expressed similar levels at 9 weeks, indicating persistent presence of HIV-1 proteins delayed TRPM7 expression. Binge exposure to EtOH (binge EtOH) decreased TRPM7 expression in control rBMVECs in a concentration dependent manner, and abolished TRPM7 expression in HIV-1Tg rats. In human BMVECs (hBMVECs), TRPM7 expression was downregulated after treatment with EtOH, HIV-1 proteins, and in combination. Next, we constructed *in vitro* BBB models using BMVECs and found TRPM7 antagonists enhanced EtOH-mediated BBB integrity changes. Our study demonstrated alcohol decreased TRPM7 expression, whereby TRPM7 could be involved in the mechanisms underlying BBB alcohol-induced damage in HIV-1 patients on cART. Supported by NIH DA046258, AA026071 and AA025964.

#### 51. IFIT2 RESTRICTS MURINE BETA CORONAVIRUS INDUCED SPINAL CORD PATHOLOGY

Sharma, M., MS ^1^, Chakravarty, D., Ph.D.^1^, Zalavadia, A., Ph.D.^2^, Burrows, A., MS^2^, Rayman, P., MS^2^, Sharma, N., Ph.D.^2^, Kenyon, L.C., MD, Ph.D.^3^, Bergmann, C., Ph.D.^4^, Sen, G.C., Ph.D.^2^, Sarma, J.D., Ph.D.^1^

^1^Department of Biological Sciences, Indian Institute of Science Education and Research Kolkata, Mohanpur, 741246

^2^Department of Inflammation and Immunity, Lerner Research Institute, Cleveland Clinic, Cleveland, OH 44195

^3^Department of Pathology, Anatomy and Cell Biology, Thomas Jefferson University, Philadelphia, PA 19107

^4^Department of Neurosciences, Lerner Research Institute, Cleveland Clinic, Cleveland, OH 44195.

IFIT2 plays a critical role in restricting neurotropic murine β-coronavirus RSA59 infection. RSA59 intracranial injection of Ifit2 deficient(−/−) compared to wild type (WT) mice results in impaired acute microglial activation, associated with reduced CX3CR1 expression, which consecutively limits migration of peripheral lymphocytes into the brain, leading to impaired virus control followed by severe morbidity and mortality. While the protective role of Ifit2 is established for acute viral encephalitis, less is known about its influence on demyelination during the chronic phase of RSA59 infection. Our current study demonstrates that Ifit2 deficiency causes extensive RSA59 viral spread throughout both the spinal cord grey and white matter and is associated with impaired CD4+ T cell infiltration. Cervical lymph nodes of RSA59 infected Ifit2−/− mice showed reduced activation of CD4+ T cells and impaired IFNγ expression during acute encephalomyelitis. Furthermore, blood-brain-barrier integrity was preserved in the absence of Ifit2 as evidenced by integral, tight junction protein ZO-1 expression surrounding the meninges and blood vessels and decreased Texas red dye uptake. In contrast to WT mice exhibiting only sparse myelin loss, the chronic disease phase in Ifit2−/− mice was associated with severe demyelination and persistent viral load, even at low infection doses. Overall, our study highlights that Ifit2 provides antiviral functions by promoting acute neuroinflammation and thereby aiding virus control and limiting severe demyelination. Supported by National Institutes of Health grant: R01-CA068782, SERB-POWER grant: SPG/2020/000454.

#### 52. THE INTERPLAY BETWEEN ZIKA VIRUS-INDUCED AUTOPHAGY AND NEURAL STEM CELL FATE DETERMINATION

Bindu, M.S. ^1^, Seth, P., Ph.D.^1^

^1^Cellular and Molecular Neuroscience, National Brain Research Centre, Manesar, 122052.

Zika virus (ZIKV) is an arbovirus belonging to the genus Flavivirus. Initially found in Uganda and has become a global health concern due to its association with congenital development disorders like microcephaly in new-borns. The mechanism behind ZIKV-induced microcephaly is not clearly understood. Proliferation arrest and premature differentiation of neural stem cells (NSC) are key mechanisms for microcephaly. As autophagy also controls NSC fate determination, we studied the effects of ZIKV on autophagy and its impact on NSC fate. Using human fetal NSCs model system, we found that co-transfection with ZIKV non-structural protein NS4A and NS4B significantly altered the NSC fate by arresting its proliferation and inducing premature neurogenesis as evident from reduced neurosphere size and increased expression of neurogenesis markers. We also found augmented expression of LC3II and reduced p62 expression depicting enhanced autophagy. Since autophagy controls NSC fate by regulating key signaling pathways like Wnt and Notch by degrading their receptors, we observed that the expression of Notch1 as well as their downstream target genes were downregulated following transfection. To confirm that reduced notch1 expression is due to autophagy-mediated degradation, we treated our cells with an autophagy inhibitor (3 methyladenine) along with co-transfection with NS4A and NS4B. We observed rescue in notch1 expression, enhanced proliferation and reduced differentiation of NSCs after drug treatment. These findings are being validated through siRNA-mediated knockdown of autophagy genes. Supported by CSIR, NBRC

#### 53. INVOLVEMENT OF LNCRNA XIST/MIRNA-124/CCL2 AXIS IN HIV-1 TAT-MEDIATED MICROGLIAL ACTIVATION AND NEUROINFLAMMATION

Singh, S., Ph.D.^1^, Buch, S., Ph.D.^1^, Periyasamy, P., Ph.D.^1^

^1^Department of Pharmacology and Experimental Neuroscience, University of Nebraska Medical Center, Omaha, NE 68198.

HIV-1 and its proteins, such as the Transactivator of Transcription (Tat), cause toxic effects in CNS cells by releasing cytokines and chemokines, leading to neuroinflammation in people living with HIV. Long noncoding (lnc) RNAs regulate gene expression by serving as competitive endogenous RNAs involving the binding of miRNAs and affecting the downstream target genes. However, the role of lncRNAs in HIV-1-mediated microglial activation and neuroinflammation remains scant. Herein, we investigate the involvement of lncRNA Xist/miR-124/CCL2 axis in mediating the cellular activation in HIV-1 Tat exposed microglial cells. Our findings demonstrated that HIV-1 Tat increased the expression of lncRNA Xist and downregulated the expression of miR-124 in mouse BV2 cells. Bioinformatics analyses also identified miR-124 as a potential novel binding site for lncRNA Xist and the 3′-UTR of CCL2. Gene silencing of lncRNA Xist and miR-124 overexpression studies further underscored the role of lncRNA Xist/miR124/CCL2 axis in HIV-1 Tat-mediated activation of BV2. The binding of lncRNA Xist with miR-124 was determined using dual luciferase assay, argonaute immunoprecipitation, and RNA immunoprecipitation assays. We also validated the *in vitro* findings using *in vivo* doxycycline-inducible Tat mice. Overall, our results suggest that HIV-1 Tat upregulates the expression of lncRNA Xist, which, in turn, sponges with miR-124, ultimately leading to increased expression of target gene CCL2 and culminating in microglia activation and neuroinflammation. Supported by This work was supported by the NIH grant: P30MH062261 (Pilot Project).

### Neurology and Therapeutics Plenary Lecture

#### 54. NOVEL HIPPOCAMPAL DRUGS FOR ALZHEIMER’S DISEASE

Pahan, K., Ph.D.^1^, Roy, A., Ph.D.^1^, Patel, D., Ph.D ^1^, Rangasamy, S., Ph.D.^1^

^1^Neurological Sciences, Rush University Medical Center, Chicago, IL 60612

^2^Research & Development, Jesse Brown VA Medical Center, Chicago, IL 60612.

Alzheimer’s disease is the most common form of dementia and until now no effective drug is available to halt its progression. During drug discovery, we always look for chemical library, microbial product archive, plant components, etc. and overlook our own body, which is gifted with almost everything needed to run on its own without much problem. Therefore, from hippocampal nuclei of good learning mice, we have isolated a number of drugs that are capable of upregulating plasticity-related molecules, stimulating NMDA- and AMPA-sensitive calcium influx and increasing spine density in cultured hippocampal neurons via peroxisome proliferator-activated receptor alpha (PPARα). Interestingly, the level of these endogenous drugs is less in the hippocampus of 5xFAD mice, a transgenic (Tg) mouse model of AD, as compared to non-Tg mice. Oral administration of these drugs increases the level of these compounds in the hippocampus to promote synaptic functions in the hippocampus and improve memory and learning in 5xFAD mice. These results suggest that hippocampus-based drugs may be explored for therapeutic intervention in AD. This study was supported by a grant from National Institutes of Health (AG050431), the Zenith Fellows Award (ZEN-17-438829) from Alzheimer’s Association and a merit award (1I01BX003033) from US Department of Veterans Affairs.

### Symposium 6: General Symposium

#### 55. CLONAL HEMATOPOIESIS IN MONOCYTES CONTRIBUTES TO HIV-ASSOCIATED NEUROINFLAMMATION

Maggirwar, S.B., MBA, Ph.D.^1^

^1^Department of Microbiology, Immunology & Tropical Medicine, George Washington University School of Medicine and Health Sciences, Washington, DC, USA.

Extravasation of inflammatory monocytes across the blood-brain barrier (BBB) in response to human immunodeficiency virus type-1 (HIV) is a critical event that leads to chronic neuroinflammation, neurologic injury, and subsequent loss of cognitive abilities in a significantly large number of infected individuals. However, given the heterogeneity of monocytes that exists in HIV-infected individuals receiving anti-retroviral therapy (ART), it remains unknown as to whether the neuro-modulatory actions of monocytes are limited to select subset of monocytes. Our studies reveal that the ART-treated HIV-infected individuals harbor higher numbers of inflammatory monocytes (CD14^low^CD16^hi^) in their circulation. These cells also exhibit characteristic features of clonal hematopoiesis (CH), such as loss of DNA methyltransferase 3A (DNMT3A) and Tet methylcytosine dioxygenase 2 (TET2) with concurrent increase in the expression of janus kinase-2 (Jak2; these three gene products, and few others, are often termed as CH drivers). CH of intermediate potential (CHIP) is a common aging-related phenomenon in which hematopoietic stem cells or other early blood cell progenitors contribute to the formation of a genetically distinct subpopulation of blood cells. Aside from age, chronic infection and inflammation have also been implicated in the development of CH. Our studies further demonstrate that the CH + inflammatory monocyte subset (1) translocate to the central nervous system (CNS) in response to ART, in platelet-dependent manner, and (2) is neuro-modulatory in action. Taken together, these results lend strong support to the notion that the priming of monocytes by activated platelets potentiates ART-mediated regulation of CH in monocytes, leading to HIV-associated neuroinflammation.

#### 56. EXTRACELLULAR VESICLE-MEDIATED AMYLOID TRANSFER AND INTERCELLULAR COMMUNICATION WITHIN THE NEUROVASCULAR UNIT

Osborne, O. ^1^, Andras, I.E.^1^, Pierre Louis, K.D.^1^, Daftari, M.T.^1^, Toborek, M.^1^

^1^Biochemistry and Molecular Biology, University of Miami Miller School of Medicine, Miami, FL, USA

It is widely accepted that elevated brain deposits of amyloid beta (Aβ) contribute to neuropathology in Alzheimer’s disease. Additionally, Aβ deposition was demonstrated to be elevated in the brains of HIV-infected patients and associated with neurocognitive decline; however, the mechanisms of these processes are poorly understood. The role of the blood-brain barrier (BBB) as an interface for the transfer of Aβ from the periphery into the brain and the cells of neurovascular unit is poorly characterized. Indeed, substantial population of neural progenitor cells (NPC) reside near brain capillaries that form the BBB. The purpose of this study is to understand the impact of brain endothelium-derived extracellular vesicles containing Aβ (Aβ-EVs) on metabolic functions and differentiation of NPCs. Mechanistically, we focused on the role of mitochondria, the receptor for advanced glycation end products (RAGE), and activation of the inflammasome on these events. We demonstrate that physiological concentrations of Aβ40 can transfer and accumulate in NPCs via endothelial EVs. This transfer results in mitochondrial dysfunction, disrupting cristae morphology, metabolic rates, fusion and fission dynamics of NPCs, as well as their neurite development. Moreover, our results show that Aβ partly colocalized with the inflammasome markers ASC and NLRP3 in the recipient NPCs. This colocalization was affected by HIV and RAGE inhibition by a high-affinity specific inhibitor, FPS-ZM1. Interestingly, both Aβ-EVs and RAGE inhibition altered NPC differentiation. Overall, our data indicates that intercellular transfer of Aβ40 is carried out by brain endothelium derived EVs that can induce mitochondrial dysfunction and alter cellular signaling, leading to aberrant neurogenesis of NPCs. These events may modulate EV-mediated amyloid pathology in the HIV-infected brain and contribute to the development of HIV-associated neurocognitive disorders. Supported by NS125905 MH072567, MH098891, HL126559, DA039576, DA040537, DA044579 and Miami CFAR-P30AI073961.

#### 57. GENETIC VALIDATION OF DRUG TARGETS: HIV-1 TAT PROTEIN-INDUCED INHIBITION OF [3H] DOPAMINE UPTAKE IN THE PREFRONTAL CORTEX OF INDUCIBLE TAT TRANSGENIC MICE IS ATTENUATED IN DOPAMINE TRANSPORTER Y88F KNOCK-IN MICE HARBORED WITHIN THE TAT TRANSGENIC MICE

Zhu, J., MD, Ph.D.^1^, Strauss, M.J., Ph.D.^1^, Jimenez Torres, A.C., Ph.D.^1^, Davis, S.E., MS^1^, Porter, K.D., BS^1^, McLaughlin, J.P., Ph.D.^3^, Zhan, C.G., Ph.D.^2^

^1^College of Pharmacy, University of South Carolina, Columbia, SC 29208

^2^College of Pharmacy, University of Kentucky, Lexington, KY

^3^College of Pharmacy, University of Florida, Gainesville, FL.

Dysregulation of dopamine (DA) neurotransmission has been linked to the development of HIV-associated neurocognitive disorders (HAND). We have reported that the human dopamine (DA) transporter (hDAT) tyrosine88 residue can form a hydrogen bond with the HIV-1 Tat residue lysine19, which plays a key role in the HIV-1 Tat-DAT interaction, leading to inhibition of DAT-mediated DA uptake. Further, we demonstrated that mutation of the tyrosine88 residue (Y88F) retained basal DAT-mediated DA uptake and attenuated *in vitro* Tat-induced inhibition of DA uptake in WT hDAT, whereas mutation of Tat lysine19 to alanine (K19A) also attenuated Tat-inhibited DA uptake. For genetic validation, this study determined whether the *in vitro* attenuation of Tat-induced inhibition of DAT activity by the DAT Y88F mutant can be replicated *in vivo* in DAT Y88F knock-in mice. We then generated a novel hybrid Y88F knock-in harbored with inducible Tat transgenic (iTat-tg) mice. Following 7- or 14-day administration of doxycycline (Dox)-induced Tat expression, the Vmax of [3H]DA uptake in the prefrontal cortex was decreased in iTat-tg mice compared to saline-treated iTat-tg mice, which was significantly attenuated in the Y88F/iTat-tg mice. Further, Dox-treated iTat-tg mice displayed a deficit in novel object recognition, which was alleviated in Dox-treated Y88F+/+/iTat-tg mice. Determining the genetic basis underlying the interaction between Tat and DAT may reveal novel therapeutic possibilities for preventing the development of HIV-associated neurocognitive disorders. Supported by NIDA/DA035714, R21DA041932 and DA041932.

#### 58. EFFECTS OF AZITHOMYCIN ON EXPRESSION AND FUNCTION OF DRUG EFFLUX TRANSPORTERS IN CELLULAR RESERVOIRS

Cory, T.J., Ph.D. ^1^, Mu, Y., Ph.D.^1^

^1^Department of Clinical Pharmacy and Translational Science, University of Tennessee Health Science Center College of Pharmacy, Memphis, TN 38016.

HIV-1 can reside in cellular reservoirs, including CD4+ T cells and monocytes/macrophages. Macrophages can exist across a spectrum of activation, ranging from the pro-inflammatory M1 phenotype to the pro-fibrotic M2 phenotype. Previously, we have shown that drug efflux transporters, including PGP, MRP1, and BCRP, are differentially expressed on M1 and M2 macrophages. The macrolide antibiotic azithromycin, which is commonly used to treat bacterial infections, has been shown to shift macrophages from the M1 to the M2 phenotype. Alterations in transporter expression on macrophages may result in altered concentrations of antiretrovirals intracellularly. We examined the effects of azithromycin on the expression and function of macrophage phenotype-specific drug efflux transporters in U937 macrophages. We used immunoblots and flow cytometry to confirm macrophages, and fluorescent dye assays and LC-MS/MS to assess transporter function. We observed that treating M1 macrophages with azithromycin reduced MRP1 expression in the cells, while also polarizing them to the M2 phenotype. Treating M2 macrophages with azithromycin decreased PGP and BCRP expression and increased the intracellular accumulation of the antiretroviral lopinavir. Supported by NIDA R01DA047178.

#### 59. GLYCOGEN SYNTHASE KINASE 3: A PROMISING THERAPEUTIC TARGET IN NEUROHIV AND HAND

Moidunny, S., Ph.D.^1^, Benneyworth, M.A., Ph.D.^2^, Titus, D.J., Ph.D.^3^, Beurel, E., Ph.D.^4^, Kolli, U., Ph.D.^1^, Meints, J., MS^2^, Jalodia, R., Ph.D.^1^, Ramakrishnan, S., Ph.D.^1^, Atkins, C.M., Ph.D.^3^, Roy, S., Ph.D.^1^

^1^Department of Surgery, University of Miami, Miami, FL 33136

^2^Department of Neuroscience, University of Minnesota, Minneapolis, MN 55455

^3^Department of Neurological Surgery, University of Miami, Miami, FL 33136

^4^Department of Psychiatry and Behavioral Sciences, University of Miami, Miami, FL 33136.

Glycogen synthase kinase 3 (GSK3) is a serine/threonine kinase that phosphorylates over 50 substrates and regulates a variety of cellular functions. Growing evidence indicate that GSK3 regulates HIV-1 replication. In addition, GSK3 inhibition reduces HIV-1 induced neurotoxicity and astroglial activation in mice. Consistently, long-term treatment with lithium, a GSK3 inhibitor, reduced central nervous system injury in HIV-1 patients. We recently investigated the role of GSK3 in development of HIV-associated neurocognitive impairments using the HIV-1-transgenic 26 (Tg26) mouse model. We found that male, but not female, Tg26 mice show deficits in spatial-learning, spatial-memory and contextual-fear memory compared to wild-type mice. Consistently, male Tg26 mice show reduced hippocampal basal synaptic transmission and long-term potentiation (LTP). These deficits in male Tg26 mice were independent of hippocampal neuronal loss but were associated with reduced hippocampal synapsin-1 protein, reduced BDNF mRNA and protein, reduced AMPA glutamate receptor (GluA)1 phosphorylation levels and increased GSK3 activity. Importantly, treatment with a non-ATP competitive GSK3 inhibitor (4-Benzyl-2-methyl-1,2,4-thiadiazolidine-3,5-dione) significantly increased hippocampal synapsin-1, BDNF and GluA1 phosphorylation levels, and restored hippocampal basal synaptic transmission, LTP and contextual-fear memory in male Tg26 mice. In summary, GSK3 is a promising therapeutic target for reducing HIV-induced neurotoxicity and improving cognition in HIV-1 patients. Supported by Miami Center for AIDS Research and National Institute on Drug Abuse.

#### 60. URINARY BLADDER MICRORNAS MEDIATE PAIN AND REDUCE THE SEVERITY OF EXPERIMENTAL CYSTITIS

Singh, Udai, Ph.D.^1^, Kiran, Sonia, Ph.D.^1^, Ahmed, Rakib, BS^1^, Mandal, Mousumi^1^

^1^University of Tennessee Health Science Center, Pharmaceutical Sciences, Memphis, TN 38103.

Interstitial cystitis (IC), also called painful bladder syndrome (PBS), affects 3 to 8 million women and 1 to 4 million men in the U.S., yet its cause and pathogenesis remain unclear. The symptoms include suprapubic pain related to bladder filling, accompanied by urgency, frequency, and discomfort in the bladder. The differential expression of microRNAs (miRs) in naïve, activated and effector immune cells suggest that miRs also serve as critical immunoregulators of IC. Here, we investigated the role of an altered set of the urinary bladder (UBs), and miRs in the pathogenesis of IC. We used cyclophosphamide (CYP) induced experimental IC, which develops a phenotype comparable to clinical IC with functional and histological alterations confined to the urinary UBs. Microarray analysis showed that 107 miRNAs from the UB had a 1.5-fold greater difference in expression of the IC than the control. Among them, we observed upregulation of a set of miRs that includes miRs-34a/b/c and miR-21a from the bladder were determined by reverse-transcription polymerase chain reaction (RT-PCR) analysis. Inhibiting these miRs alters detrusor smooth muscle (DSM) function in IC. We also noticed significant infiltration of macrophages, neutrophils, and mast cells in IC as compared to the control. The histological UB inflammatory scores are also elevated in IC as compared to the control. The mechanisms associated with IC induction might be due to the alterations of immune cells by CYP induction, which modulate specific miRs that increase the local inflammatory response in the bladder. Supported by Supported in part by NIH grant R01 AI140405.

#### 61. DISRUPTED INTERFERON TYPE I SIGNALING IN PLASMA, MONOCYTES AND HUMAN BRAIN ORGANOIDS FROM PEOPLE LIVING WITH HIV WITH COGNITIVE IMPAIRMENT

Cantres-Rosario, Y.M., Ph.D.^1^, Medina Colón, E., BS^1^, Collazo, B., BS^1^, Rodriguez, E., BS^1^, Díaz, B., Ph.D.^2^, Rodriguez, R.J., Ph.D.^2^, Matos, M., MD^1^, Sepúlveda, V., MD^1^, Gerena, Y., Ph.D.^1^, Wojna, V., MD^1^

^1^School of Medicine, Univ. of Puerto Rico, Medical Sciences Campus, San Juan, PR 00935

^2^Psychology Department, Univ. of Puerto Rico, Rio Piedras Campus, San Juan, PR 00925.

People living with HIV (PWH) develop neurocognitive disorders, driven by monocytes infiltrating into the brain and neuronal dysfunction. We hypothesize that disrupted Interferon type I (IFN-I) signaling promotes monocyte infiltration and cognitive decline. We measured Interferon alpha (IFNa1/2) and beta (IFN-b) in the plasma of PWH, Alzheimer’s disease (AD) patients and HIV-negative controls by ELISA and cytokine array. Then, we measured interferon alpha receptor 1 (IFNAR1) in monocytes from patient-derived peripheral blood mononuclear cells (PBMCs), by flow cytometry. IFNa1 levels were slightly higher in the plasma of cognitive impaired PWH and significantly higher in AD patients (p=0.035), compared to controls. IFN-b decreased in normal cognitive PWH compared to HIV-negative controls, in both male and female participants. IFNa2 was higher in the plasma of cognitive impaired patients, compared to normal cognitive PWH (p=0.02). The percentage of IFNAR1 + CD14 + monocytes was decreased in PBMCS from PWH (p=0.02), and AD patients (p=0.011) compared to controls, more evident in monocytes from male participants (p=0.03). However, western blots revealed that Interferon Regulatory Factor (IRF3) phosphorylation in Ser386 is higher in PBMCs from PWH compared to controls (p=0.036), thus active. Upon co-culture with these monocytes, human brain organoids also exhibited decreased IFNAR1 and elevated pIRF3, which decreased with cognitive impairment. Thus, disrupted IFN-I signaling in monocytes may contribute to their infiltration to the brain, neuropathology, and cognitive decline in PWH. Supported by K22NS118975, R01NS099036, U54MD007600, U54GM133807.

#### 62. LONG-ACTING NANOFORMULATIONS PREVENT DOLUTEGRAVIR INDUCED OXIDATIVE STRESS IN EMBRYO BRAIN

Bade, A.N., Ph.D. ^1^, Foster, E.G., BS^1^, Liu, Y., Ph.D.^2^, Edagwa, B.J., Ph.D.^1^, Gendelman, H.E., MD^1^

^1^Department of Pharmacology and Experimental Neuroscience, University of Nebraska Medical Center, Omaha, NE 68198

^2^Department of Radiology, University of Nebraska Medical Center, Omaha, NE 68198.

Dolutegravir (DTG) is currently recommended as part of first-line treatment regimens for HIV-1 patients. Nonetheless, concerns emerged for its usage in pregnant women. Notably, DTG-based regimens have been linked to neural tube defects and postnatal neurodevelopmental deficits. To this end, the need for intervention strategies that would maximize DTG’s efficacy benefits while limiting adverse events cannot be overstated. Thus, we created and evaluated long-acting nanoformulated DTG (NDTG) injectable. Herein, pregnant female C3H/HeJ mice were either treated orally every day with DTG at 5 mg/kg starting at gestation day (GD) 0.5, with a single intramuscular (IM) injection of NDTG (45 mg/kg) at GD 0.5 or with two NDTG (25 mg/kg) intramuscular injections, first at GD 0.5 and second at GD 9.5. We determined that both NDTG and daily oral DTG groups achieved plasma DTG levels in dams comparable to therapeutic DTG concentrations from daily oral dosing in humans (4000–6500 ng/mL). However, five-fold lower DTG levels were observed in embryo brain following NDTG injections in comparison to daily oral administration. Further, magnetic resonance imaging scanning of live dams at GD 17.5 to acquire T1 maps of fetal brain identified improved T1 relaxation time in both NDTG-treated group compared to oral DTG group reflecting prevention of oxidative stress mechanism through reduced drug exposure during neurodevelopment. Overall, we show that novel long-acting drug delivery approach can minimize embryo brain DTG exposure and thus, potentially limit drug related neurodevelopmental toxicities. Supported by NICHD – Eunice Kennedy Shriver National Institute of Child Health and Human Development.

#### 63. CD47 DELETION IN LYMPHATIC ENDOTHELIUM AUGMENTS ARTERIAL LYMPHANGIOGENESIS AND ATTENUATES ATHEROSCLEROSIS.

Singla, B., Ph.D.^1^, Aithabathula, R.V., Ph.D.^1^, Pervaiz, N., Ph.D.^1^, Kathuria, I., Ph.D.^1^

^1^Department of Pharmaceutical Sciences, The University of Tennessee Health Science Center, Memphis, TN 38163.

Thrombospondin-1 (TSP1) mediates its physiological effects via interacting with CD36 and CD47 receptors. TSP1 is a well-known angiogenesis inhibitor, however, its role in regulating lymphangiogenesis is not clear. We recently reported that global CD47-deficient mice are protected from atherosclerosis, however, myeloid cell-specific CD47 loss augments lesion formation suggesting the importance of cell-specific CD47 blockade in atherosclerosis. Our experiments revealed CD47 as a dominant TSP1’s receptor in lymphatic endothelial cells (LECs). As the lymphatic vasculature is functionally linked to atherosclerosis, we herein investigated the effects of LEC TSP1-CD47 signaling inhibition on lymphangiogenesis and atherosclerosis. We observed elevated TSP1 expression in human and mouse atherosclerotic arteries compared to non-atherosclerotic tissue. TSP1 at pathological concentrations inhibited VEGF-C-stimulated *in vitro* lymphangiogenesis. Mechanistically, TSP1 inhibited VEGF-C-induced Akt and eNOS activation in LECs, leading to attenuated nitric oxide production. Further, TSP1-treated Cd47-silenced LECs proliferate faster and have higher Akt and eNOS activation. AAV8-PCSK9-injected LEC-specific Cd47 knockout mice had reduced atherosclerosis compared with control mice. Additionally, LEC-specific Cd47 deletion improved lymphatic vessel density in mice. These results demonstrate that TSP1-mediated LEC CD47 activation inhibits lymphangiogenesis and contributes to atherosclerosis. Altogether, these findings identify LEC CD47 as a potential therapeutic target in atherosclerosis. Supported by NIH-NHLBI.

#### 64. THE ROLE OF THE NON-CANONICAL INFLAMMASOME IN NEUROINFLAMMMATION

Amer, A., MD, Ph.D.^1^, Daily, K., Ph.D.^1^, Eltobgy, M., MD^1^, Badr, A., MD, Ph.D.^1^

^1^Microbial Infection and Immunity, The Ohio State University, Columbus, OH 43210.

Alzheimer’s disease (AD) is a progressive neurodegenerative and neuroinflammatory disorder and a major leading cause of death in the world. Currently, there are no reliable preventative methods or a cure for AD. Brain pathology in AD is characterized by extracellular senile plaques of amyloid-beta (AB) and intracellular neurofibrillary tangles of tau protein. Neuroinflammation in AD is coordinated by progressive changes in brain inflammatory cells, such as microglia and brain-associated macrophages. To determine the contribution of the inflammasome to AD pathobiology and neuroinflammation, we generated 5XFAD mouse lacking the expression of essential inflammasome members. We found that the lack of a major member of the inflammasome prevents the accumulation of AB, reduced microglia activation and improved mouse behavior. Therefore, members of the inflammasome can be viable targets to prevent AD. Supported by NIH.

## POSTER ABSTRACTS

### INVESTIGATOR POSTERS (I1–I18)

#### 1. DOLUTEGRAVIR REDUCES MIGRATION ABILITIES OF TROPHOBLASTS

Bade, A.N., Ph.D.^1^, Speakar, B.^2^, Foster, E.G., BS^1^, Shukla, S.K., Ph.D.^3^, Gendelman, H.E., MD^1^

^1^Department of Pharmacology and Experimental Neuroscience, University of Nebraska Medical Center, Omaha, NE 68198

^2^Department of Biology, University of Nebraska, Lincoln, NE 68588

^3^Department of Oncology Science, University of Oklahoma Health Sciences Center, Oklahoma City, OK 73104.

Dolutegravir (DTG) is a first-line antiretroviral drug used in combination therapy for the treatment of human immunodeficiency virus type-1 (HIV-1) infection. However, concerns were raised that DTG may affect fetal development. As DTG is broadly prescribed to women of child-bearing age drug-associations with birth defects has continued to generate interest amongst HIV/AIDS researchers. Thus, uncovering mechanisms for DTG-linked affects on fetal developmental remains a priority. To this end, there is a knowledge gap of effects of DTG on placental development. Thus, we employed cell cultures of HTR-8 trophoblastic cells to interrogate the effects of clinically relevant DTG concentrations under normoxic and hypoxic conditions. DTG was found to inhibit activities of MMP-2 under both, normoxic and hypoxic, conditions. Moreover, DTG treatment decreased expression of HIF-1α under the same conditions. Interestingly, decrease in expression of beclin-1 protein was observed, suggesting a potential role of autophagy in HIF-1α reduction. Further assessments assessing the effects of DTG exposure on trophoblasts functions showed that DTG reduces migration and invasion abilities of HTR-8 cells. Finally, studies performed in pregnant mice validated that *in utero* DTG exposure decreases HIF-1α expression in placenta. Altogether, we conclude that DTG can potentially impair placental development through affected HIF-1α-MMPs pathway, which could lead to drug related neurodevelopmental toxicities. Supported by NICHD – Eunice Kennedy Shriver National Institute of Child Health and Human Development.

#### 2. AMPHIPHILIC CARBON DOTS AS POTENTIAL NANOCARRIERS TO TARGET HUMAN BETA-AMYLOID PLAQUES IN AN IN-VITRO MODEL

Castro, R., BS ^1^, Devadoss, D., Ph.D.^1^, Zhou, Y., Ph.D.^2^, Lakshmana, M.K., Ph.D.^1^, Leblanc, R., Ph.D.^2^, Chand, H.S., Ph.D.^1^

^1^Department of Immunology and Nanomedicine, Florida International University, Miami, FL 33199

^2^Department of Chemistry, University of Miami, Coral Gables, FL 33146.

Accumulation of β-amyloid (Aβ) plaques is one of the key pathologic features observed in Alzheimer’s disease (AD). Nanoparticles are currently being investigated because they can cross the blood-brain barrier and improve the bioavailability of therapeutics. Carbon-dots (CDs) are promising nanocarriers that has low cytotoxicity and high biocompatibility for clinical research. Following ultrasonication of citric acid and o-phenylenediamine, we synthesized amphiphilic CDs with a 3 nm hydrodynamic diameter and emit an excitation-independent photoluminescence. The primary amine and carboxyl group content were 0.06 and 8.0 nmol/mg of CDs, respectively, indicating the potential for bioconjugating small-molecules. Because carbon nanomaterials have previously shown to inhibit Aβ aggregation, we tested the efficacy CDs on Aβ plaque formation using a mammalian cell line that overexpress amyloid precursor protein (APP-751). The APP production was assessed by immunofluorescence and the secretory Aβ40 levels in the conditioned media (CM) were measured using ELISA. The structured-illumination microscopy revealed that CDs entered the cells and inhibited APP-751 expression in a dose-dependent manner. Compared to the untreated cells CM, there was ∼20 % reduction in the secreted Aβ40 in the CM of cells treated with 10 µM CDs. Further studies are currently being performed to help understand the mechanisms by which CDs affect the Aβ expression and aggregation. Nonetheless, these data suggest that amphiphilic CDs have a great potential as nanocarriers for CNS-targeting against AD pathologies.

#### 3. ASTROCYTE-MEDIATED MODULATION OF MICROGLIAL INFLAMMATION IN JAPANESE ENCEPHALITIS VIRUS (JEV) INFECTION

Chakraborty, T., BS^1^

^1^Molecular Neuroscience, National Brain Research Centre, Gurgaon, 122051.

Astrocytes and microglia are the major glial cells present in mammalian brain. Functionally different, how these two cell types interact among themselves under pathological conditions is yet to be resolved. In our present study, we aim to investigate the molecular crosstalk between astrocytes and microglia under conditions of flaviviral infection. Upon infecting the astrocytes (C8D1A cell line) with Japanese Encephalitis Virus (JEV), astrocytes secrete certain cytokines which is a part of the innate immune response. The supernatant of the astrocyte thus contain viral proteins, viral particles as well as cytokines. The microglial cells (N9 cell line) upon incubation with the supernatant of astrocyte show depletion of JEV replication at the protein level. The microglial cells also show a decrease in the expression of various antioxidant proteins along with an increase in the production of reactive oxygen species (ROS). There is also an increment in the level of pro-inflammatory cytokines in microglial cells which is indicative of increased inflammatory condition. Thus our findings elucidate that astrocytes can modulate the physiological dynamics of the microglial cells under conditions of JEV infection, by increasing overall inflammation and ROS generation in them. However, further experiments are required to find out the mechanism behind the factors involved in astrocyte supernatant-mediated modulation of microglial inflammation. Supported by J.C.Bose Fellowship (SERB) (JCB/2020/00037).

#### 4. AGING- AND HIV-1 TRANSGENE-MEDIATED SHIFTS OF GENE EXPRESSION PROFILE IN THE LIVER

He, J.H., Ph.D.^1^, Xu, C.X.^1^, Huang, W.H.^2^, Chang, S.L., Ph.D.^2^

^1^Third Institute of Oceanography, Ministry of Natural Resources, Xiamen, 361005

^2^Institute of NeuroImmune Pharmacology, Seton Hall University, South Orange, NJ 07079

^3^Department of Biological Sciences, Seton Hall University, South Orange, NJ 07079.

Little research is to study how HIV and aging affect liver. We studied mRNA expression profiles retrieved from Gene Expression Omnibus Profiles. VWA5A was downregulated in HIV patients given HAART while BUB1B, CDKN1C, LSM5 and LMNB1 were downregulated with the aging F344 rats. The HIV-1Tg rats were used to investigate the combined effects of HIV and aging on gene expression in liver. Young (1-mo.) F344, young HIV-1Tg, old (13-mo.) F344 and old HIV-1Tg were studied. Expression of 84 genes related to aging liver were determined. Minor difference was found between young F344 and HIV-1Tg rats. There were profound differences between two strains of aged animals. VWA5A was down-regulated in both young and old HIV-1Tg rats. BUB1B and COL1A1 were both downregulatedin the old HIV-1Tg and F344 rats. CDKN1C, LSM5 and LMNB1 were downregulated and C4A and TOLLIP were up-regulated in old HIV-1Tg rats, but not significantly in old F344 rats. The biological process in which the altered genes were involved and their connection to HIV and aging were identified by Coremine Medical online, Ingenuity Pathway Analysis and Connectivity Map. Two hypolipidemic statin agents, Mevastatin and Lovastatin, were among the top candidate inhibitors. PCR Array confirmed aging-induced regulation of genes related to statin pathway in presence of HIV. Our results suggested that statin pathway may be involved in aging liver with HIV-1 infection and statin has therapeutic potential to liver pathology related to aging in HIV patients. Supported by NIH grants AA023172 and DA036175.

#### 5. ROLE OF MIRNAS IN EPHRINA3 SIGNALLING

Jaiswal, P., MS ^1^, Singal, C.M.S., MS^1^, Seth, P., Ph.D.^1^

^1^Molecular and Cellular Neuroscience, National Brain Research Centre, Gurugram, 122052.

Neurological involvement of HIV-1 impairs cognition which leads to Neuro-AIDS. HIV-1 infected astrocytes harbour the virus that disintegrates the neuron-glia cross-talk by releasing various proteins like HIV-1 encoded Tat (transactivator of transcription). The proteins Ephrin A3 and Ephrin A4 expressed in astrocytes and neurons respectively play juxtacrine signalling. In our other study neuron-astrocyte co-culture system was established, by isolating the human progenitor cells from fetal brain. It was found that HIV 1-Tat contributes towards glutamate excitotoxicity by upregulating the expression of EphrinA3 on astrocytes that further regulates the glutamate transporters. To unravel the effect of miRNAs on HIV 1-Tat transfected astrocytes expressing Ephrin A3, Next Generation Sequencing was performed. From the list of downregulated miRNAs, those targeting the EphrinA3 were screened using TargetScan software. The selected miRNAs were validated using qPCR. The qPCR analysis showed that hsa-miR-181a-5p and hsa-miR-4792 miRNAs were significantly downregulated. These two miRNAs will be validated using their mimics and inhibitors and target validation for EphrinA3 will be done by Luciferase assay.

#### 6. COMPARATIVE ANALYSIS OF MOLECULAR METHODS (RT-LAMP, RT-PCR & REAL-TIME RT-PCR) FOR THE DETECTION OF INFLUENZA VIRUSES

Kaushik, Samander, Ph.D. ^1^, Sharma, Vikrant, Ph.D.^1^

^1^Centre for Biotechnology, Maharshi Dayanand University, Rohtak, HI 124001.

Aims: Influenza is a serious threat to the human population worldwide, therefore, continuous surveillance is required to update influenza seasonal vaccines. A rapid, sensitive, specific and cost-effective diagnostic method will be much helpful for patient management in the present scenario. The present study is conceptualized for the detection of influenza viruses by molecular methods and compare with virus isolation. Study Design: Standard strains of the Influenza virus were used to standardize the molecular diagnostic assays and results were then compared with virus isolation. Place and Duration of Study: Centre for Biotechnology, Maharshi Dayanand University, Rohtak, Haryana, India. Methodology: Standard strains of Influenza A and B virus were used for influenza virus isolation using virus culturing in MDCK (Madin-Darby Canine Kidney) cell line by following standard tissue culture procedure. Isolated viruses were detected by the Hemagglutination assay (HA) and typed by the Hemagglutination inhibition assay (HI). Conventional one-step RT-PCR, Taqman real-time RT-PCR and RT-LAMP (Reverse transcription loop-mediated isothermal amplification) were standardized on RNA extracted from standard strains. The sensitivity and specificity of these molecular methods were compared with each other as well as with virus culture (gold standard). Results: Both influenza A and B virus strains were cultured in MDCK cells and produced cytopathic effect during virus culture. Conventional RT-PCR and real-time RT-PCR detected both types of Influenza viruses. RT-LAMP also successfully deleted. Supported by Nil.

#### 7. METHAMPHETAMINE AUGMENTATION OF HIV-1 GP120 NEUROTOXICITY VIA GLUN2B NMDA RECEPTOR SIGNALING

Liu, J.N., MD, Ph.D.^1^, Xiong, W.B., MD^1^, Dutta, D., Ph.D.^1^, Xiong, H., MD, Ph.D.^1^

^1^Department of Pharmacology and Experimental Neuroscience, University of Nebraska Medical Center, Omaha, NE 68198.

HIV-1-associate neurocognitive disorders (HAND) remain prevalent despite combined antiretroviral therapy (cART). Methamphetamine (Meth) abuse exacerbates HAND. Although the mechanisms for HAND pathogenesis remain obscure ample evidence indicate that HIV-1 glycoprotein 120 (gp120) plays an important disease-inciting role that is fueled by Meth abuse. To understand how Meth exacerbates HAND we investigated comorbid effects of Meth and gp120 on primary cortical neuronal cultures and hippocampal slices prepared from SD rats of either sex. Treatment of neuronal cultures with Meth and gp120 in combination (M + G) significantly reduced neuronal viability detected by MTT essay and increased neuronal apoptosis revealed by TUNEL staining, in contrast to no apparent effect when applied each alone. Application of selective GluN2B receptor antagonists ifenprodil/memantine significantly blocked M + G induced apoptosis, but not the viability, suggesting an involvement of GluN2B receptors in M + G-induced neuronal injury. M + G also reduced Mito Tracker dye in the mitochondria, indicting an impairment of mitochondria function. Further studies uncovered M + G increased expression levels of CaMKII and STEP and decreased the expression levels of p-ERK and BDNF. Moreover, M + G enhanced the levels of GluN2B expression which was attenuated by a Sigma-1 receptor blocker PD1047, further support the involvement of GluN2B in M + G-mediated neuronal injury. Taken together, these results demonstrated that Meth augmented gp120-induced neurotoxicity via GluN2B signaling. Supported by NIH R01DA050540.

#### 8. PUBERTY INVOLVEMENT IN BINGE EXPOSURE TO ETHANOL-INDUCED DIFFERENTIAL SPLEEN ATROPHY IN ADOLESCENT F344 RATS

Liu, X., Ph.D. ^1^, Huang, W.^2^, Chang, S.L., Ph.D.^2^

^1^Department of Histology and Embryology, Huazhong University of Science and Technology, Tongji Medical College, Wuhan, 430030

^2^Institute of NeuroImmune Pharmacology, Seton Hall University, South Orange, NJ 07079

^3^Department of Biological Sciences, Seton Hall University, South Orange, NJ 07079.

We previously reported that binge ethanol (EtOH) differentially induces spleen atrophy in male adolescent F344 rats. In a follow-up study, the differential response of spleen to binge EtOH [4.8 g/kg/d; 52 % w/v; i.g.; PND36-38] between the male and female rats was examined at PND39. The binge EtOH differential induction of spleen atrophy was observed only in the male rats. Using qRT-PCR Array analysis to determine expression of 158 genes related to alcohol metabolism, alcoholism and immune regulation, significant change in expression of 30 genes in the male rats versus 3 genes in the female was observed. Sex hormones are involved in the sex-bias in immunity. Rats reach sexual maturity at approximately PND32-34 and PND45-48 in females and males, respectively. To examine if the observed sex-dependent responses at PND39 is related to the animal’s puberty stages, gene expression was examined in the naïve rats at PND25 of which neither gender has entered puberty. Among the158 genes examined, expression of 8 genes was significantly different between male and female rats at PND25 while expression of 29 genes was significantly different between two genders at PND39. We also found that i.g. injection induced significant expression regulation of 69 genes in female rats and 59 genes in male rats. These data suggest that male F344 adolescent rats were more responsive than female rats to binge EtOH. Differential regulation of gene expression contributing to the sex-dependent responses to binge exposure to EtOH appears to be closely related to the puberty stages of these adolescent rats. Supported by AA025964.

#### 9. THE ROLE OF INTERFERON REGULATOR FACTOR 1 IN DSDNA- OR DSRNA-MEDIATED INNATE IMMUNE ACTIVATION OF HUMAN ASTROCYTES

Liu, Y., Ph.D. ^1^, Xu, X., Ph.D.^2^, Zhang, B., Ph.D.^3^, Liu, H., Ph.D.^2^, Shao, D., MS^2^, Meng, F., Ph.D.^1^, Liu, J., Ph.D.^1^, Wang, X., Ph.D.^1^, Ho, W., MD, M.Ph.^1^

^1^Lewis Katz School of Medicine, Temple University, Philadelphia, PA 19140

^2^School of Basic Medical Sciences, Wuhan University, Wuhan, 430071

^3^Institute of Hematology & Blood Diseases Hospital, Chinese Academy of Medical Sciences & Peking Union Medical College, Tianjin, 300000.

Human astrocytes are the primary components for the human blood-brain barrier (BBB), which possesses multiple pattern recognition receptors (PRRs) to recognize exogenous dangerous signals, such as viral genomes, dsDNA and dsRNA. Universally, Interferon regulator factors 3 (IRF3) and 7 (IRF7) are two principal transcription factors in modulating IFNs expression, which are responsible for activating IFNs signaling pathway and regulating the production of antiviral IFN-stimulated genes (ISGs). In this study, we observed that human primary astrocytes and an astroglioma cell line (U373) expressed IRF1 remarkably when astrocytes was stimulated with dsDNA or dsRNA. Using CRSIPR/Cas9 technology, we found that IRF1 played a pivotal role in dsDNA- or dsRNA-mediated IFNs and ISGs expression in human astrocytes. In addition, human recombinant IFNs (hrIFNs) upregulated IRF1 expression in astrocytes, and IRF1 knockout suppressed hrIFNs-mediated ISGs productive. These findings indicated that IRF1 may be a significant and unidentified modulator regulating the innate immune activation of human astrocytes. Supported by the National Institutes of Health grants DA041302, DA042373, and DA045568.

#### 10. CRISPR-CAS9 MEDIATED EXCISION OF HIV-1 TRANSGENE FOR POST-HIV ERADICATION STUDIES IN THE HIV-1 TG RAT MODEL

Kaminski, R., Ph.D.^1^, Mack, M.^2^, Booze, R.M., Ph.D.^3^, Chang, S.L., Ph.D.^2^

^1^Center for Neurovirology and Gene Editing/Dept. of Microbiology, Immunology and Inflammation, Lewis Katz School of Medicine, Philadelphia, PA 19140

^2^Institute of Neuroimmune Pharmacology/Department of Biological Sciences, Seton Hall University, South Orange, NJ 07079

^3^Program in Behavioral Neuroscience/Department of Psychology, University of South Carolina, Columbia, SC 29208.

HIV-1 Tg rat is a useful tool to model comorbidities experienced by people living with HIV on antiretroviral therapy. Persistent HIV-1 transgene expression leads to progressive HIV-1 proteinotoxicity, which can be further intensified by the use of addictive substances. We hypothesize that by using the CRISPR-Cas9 gene-editing platform to excise HIV-1 transgene sequences, we can generate a post-HIV eradication model in an HIV-1 Tg rat. Two independently performed whole-genome sequencing analyses, using genomic DNA from liver tissues or embryo fibroblasts of Tg animals, led to the identification of two possible HIV-1 transgene integration sites in chromosomes 10 and 13. Next, the Pinpoint FISH assay confirmed the presence of a single integration site HIV-1 transgene sequences in chromosome 10. The integration site in chromosome 10 was further verified by Sanger sequencing of PCR amplicons spanning rat and HIV-1 sequences. Based on these data, we were able to develop a new, simplified CRISPR-Cas9-mediated transgene excision strategy. A pair of gRNAs targeting 5′ and 3′ flanking regions of the integration site in chromosome 10 was designed and cloned into AAV and lentiviral delivery vectors. Presented results set the stage for further validation *in vivo* in HIV-1 Tg rats. Successful removal of transgene sequences from different tissues and organs of transgenic animals should lead to partial/complete reversion of HIV-1 expression-mediated pathologies. Supported by AA026071 (RK &SLC); DA046258 (SLC) and HD043680, MH106392, DA013137, NS100624 (RMB).

#### 11. CHARACTERIZING THE NEUROINFLAMMATION ASSOCIATED WITH SEQUENTIAL TRAUMATIC BRAIN INJURY IN A MOUSE MODEL

Krishna, V.D. ^1^, Crane, A.T.^2^, Emmitt, N.L.^1^, Chrostek, M.R.^2^, Grande, A.W.^2^, Low, W.C.^2^, Cheeran, M.C.-J.^1^

^1^Veterinary Population Medicine Department, University of Minnesota, Saint Paul, MN 55108

^2^Department of Neurosurgery, University of Minnesota, Minneapolis, MN 55455.

Neuroinflammation is recognized as an important secondary injury mechanism that initiates and potentiate neurological pathologies following traumatic brain injury (TBI). Repeated injuries that are common among soldiers and athletes have been shown to predispose for development of cognitive impairment and neurodegenerative disease. We hypothesized that after a mild TBI, a repeat injury results in enhanced neuroinflammatory response to the second injury. To test this hypothesis, we used controlled cortical impact (CCI) mouse model of TBI and characterized the immune cells phenotypes at various times following a single moderate injury. The number of neutrophils and CD45(hi) CD11b(+) infiltrating macrophages increased significantly in the ipsilateral hemisphere compared to sham injury at 24 h and 3 d post injury (dpi), respectively. Notably, a biphasic macrophage activation state in the brain was observed, where proinflammatory CD86(+) macrophage numbers increased at 3 dpi then reduced to control level by 7 dpi and increased at 30 dpi. Although there was no change in immune cell numbers on contralateral hemispheres after single moderate injury 7 dpi, increase in infiltrating macrophages and MHC II(+) activated microglia were seen in the contralateral hemisphere in brains that had a previous mild injury in the same hemisphere 7 d prior to a second moderate injury. Studies are underway to deplete macrophages as well as modulate macrophage activation profile after the initial injury to determine if prior macrophage activation predisposes for an exaggerated second injury response. Supported by Minnesota Spinal Cord and Traumatic Brain Injury Research Grant Program, MN Office of Higher Education, State of MN.

#### 12. ANTI-INFLAMMATORY AND BLOOD BRAIN BARRIER PROTECTIVE EFFECTS OF NOVEL ORALLY BIOAVAILABLE CANNABINOID TYPE 2 RECEPTOR AGONISTS

Persidsky, Y., MD, Ph.D.^1^, Rom, S., Ph.D.^1^, Zuluaga-Ramirez, V., MS^1^, Gaghate, S., MS^1^, Grether, U., Ph.D.^3^, Guba, W., Ph.D.^3^, Reichenbach, N.L., BS^1^, Van der Stelt, M., Ph.D.^2^, Ullmer, C., Ph.D.^3^, Pacher, P., Ph.D.^4^

^1^Department of Pathology Laboratory Medicine, Lewis Katz School of Medicine, Temple University, Philadelphia, PA 19140

^2^Department of Bio-organic Synthesis, Leiden University, Leiden

^3^Roche Innovation Center, F. Hoffmann-La Roche Ltd., Basel

^4^Laboratory of Cardiovascular Physiology and Tissue Injury, NIAAA, Bethesda, PA 20852.

We studied new orally bioavailable CB2 receptor agonists using both *in vitro* and *in vivo* models of BBB. Utilizing non-forceful feeding technique and aseptic localized encephalitis we demonstrated high efficacy of new agonists to prevent leukocyte-endothelial interaction in cerebral microvasculature and migration across BBB. New agonists were able to offset BBB permeability in vivo model of systemic inflammatory response (LPS-induced), as well to improve barrier tightness shown by increased transendothelial resistance (TEER) measurement in an *in vitro* BBB model, utilizing primary human brain microvascular endothelial cells (BMVEC). CB2 agonists diminished cytokine release and expression of the adhesion molecules, VCAM-1 and ICAM-1, required for leukocyte adhesion, as well as attenuated de-regulation of variety genes involved in inflammation and endothelial injury responses in microvessels isolated from LPS-treated mice. Pre-treatment of either BMVECs or primary human monocytes prevented monocyte attachment to and migration across the endothelial monolayer in an *in vitro* BBB models. Our results reveal anti-inflammatory effects of novel orally bioavailable selective CB2 agonists in monocytes and endothelial cells, providing treatment opportunities for insidious chronic inflammatory disorders inside and outside of CNS.

#### 13. CELL TARGET SPECIFICITY OF POLYETHYLENIMINE (PEI)-MANNOSE (MAN)-SIBECLIN1 NANOFORMULATION FOR RECEPTOR-EXPRESSING GLIA WITH THE POTENTIAL APPLICATION FOR HIV THERAPEUTICS

Rodriguez, M., Ph.D.^1^, Muthu Karuppan, M.K., Ph.D.^1^, El-Hage, N., Ph.D.^1^

^1^Department of Immunology and Nano-Medicine, Herbert Wertheim College of Medicine, Florida International University, Miami, FL 33199.

We have previously shown that silencing the host autophagic protein, Beclin1, in HIV-infected human microglia and astrocytes restricts HIV replication and viral-induced inflammatory molecules. Using human glial cell cultures, we have validated the efficacy of siBeclin1 as an adjunctive therapy in attenuating HIV when co-exposed with individual (emtricitabine, abacavir, lopinavir and atazanavir) or combined (tenofovir and emtricitabine) antiretrovirals (ARVs). Intranasal delivery of siBeclin1-encapsulated with polyethylenimine (PEI) polymer showed a safe and effective delivery of the polyplex in brain of adult mice. Morphological assessments by immunofluorescence labeling and Nissl staining, revealed no noticeable differences between glia and neurons in post-mortem brain tissues of mice treated with polyplex when compared to PBS-treated animals. To avoid non-specific targeting in the brain, we coupled the siBeclin1 to a PEI-polyplex conjugated with mannose (Man) particles that target mannose-expressing glial cells, and not neurons. *In vitro* studies confirmed that cells transfected with PEI-Man bound to Fluorescein isothiocyanate (FITC) showed green labeling in GFAP-expressing astrocytes and Iba1-expressing microglia but not in Map2-expressing neurons. Viability assay showed minimal toxicity in glia and neurons transfected with PEI-Man polyplex. Current studies are validating the bio-distribution and the efficacy of the siBeclin1-PEI-Man polyplex as an adjunctive therapy in HIV-infected humanized mouse model with and without ARVs. Supported by NIH MH118985 awarded to NEH.

#### 14. PRENATAL OPIOID EXPOSURE ALTERS THERMAL PAIN SENSITIVITY IN FEMALE OFFSPRING.

Singh, S., BS ^1^; Gomez, D., BS^1^; Abu, Y., BS^1^; Roy, S., PhD^1^

^1^Department of Surgery, University of Miami, Miami, FL 33136.

Opioid use disorder has increased in women of reproductive age, correlating with a rapid increase in neonates born with a history of prenatal opioid exposure. How prenatal opioid exposure affects pain sensitivity in female offspring is an area of great interest, as this may perpetuate the opioid epidemic. However, to date, this has largely been unexplored. In this study, we investigated how prenatal opioid exposure impacts thermal pain sensitivity in female offspring, using the tail-flick assay. Female C57 BL/6 mice were administered an escalating dose ramp of hydromorphone to create dependency, after which they were mated with drug naïve males and transitioned to methadone during pregnancy. Control mice received subcutaneous injections of saline throughout pregnancy and weaning. 12-week-old female prenatally exposed offspring were subjected to the tail flick assay. Our preliminary results show increased thermal withdrawal threshold in female opioid-exposed offspring compared to controls, suggesting hyposensitivity to thermal pain. Together, our studies provide evidence that opioid exposure during critical developmental windows during pregnancy have lasting effects on offspring. Supported by F31DA053795, R01 DA050542, R01 DA047089, R01 DA043252 and R01 DA044582

#### 15. EFFECT OF BENZO(A)PYRENE ON OXIDATIVE STRESS AND INFLAMMATORY MEDIATORS IN ASTROCYTES AND HIV-INFECTED MACROPHAGES

Sinha, N., BS ^1^, Kumar, A., Ph.D.^1^, Kodidela, S., Ph.D.^1^, Zhou, L., MS^1^, Singh, U.P., Ph.D.^1^, Kumar, S., Ph.D.^1^

^1^Department of Pharmaceutical Sciences, University of Tennessee Health Science Centre, MEMPHIS, TN 38163.

Benzo(a)pyrene (BaP), an important polycyclic aromatic hydrocarbons (PAH) component of cigarette/tobacco smoking, is known to cause adverse health effects and is responsible for various life-threatening conditions including cancer. In this study, we examined the acute (up to 72 h) effects of BaP on the expression of antioxidant enzymes (AOEs), cytokines/chemokines, and cytochromes P450 (CYP) enzymes in astrocytic cell lines, SVGA, and chronically HIV-infected U1 macrophage. The treated cells were examined for mRNA, protein levels of CYPs, AOEs superoxide dismutase-1 (SOD1) and catalase (CAT), cytokines/chemokines, using Western blot, multiplex ELISA, and reactive oxygen species (ROS) by flow cytometry analysis. Upon acute exposure, BaP (1 μM) showed a significant increase in the mRNA levels of CYPs (CYP1A1 and CYP1B1), and pro-inflammatory cytokine IL-1β in SVGA cells following BaP for 24, 48, and 72 h. In addition, we also observed a significant increase in the mRNA levels of SOD1 and CAT at 24 h of BaP treatment. In contrast, BaP did not exert any change in the protein expression of AOEs and CYP enzymes. In U1 cells, however, we noticed an interesting increase in the levels of MCP-1 as well as a modest increase in TNFα, IL-8 and IL-1β levels observed at 72 h of BaP treatment but could not reach to statistically significant level. Overall, these results suggest that BaP contributes in part to macrophage and astrocyte-mediated neuroinflammation by mainly inducing IL-1β and MCP-1 production, which is likely to occur with the involvement of CYP and/or oxidative stress pathways. Supported by NIH grants DA047178 and MH125670.

#### 16. NATURAL KILLER T (NKT) CELL ACTIVATING GLYCOLIPID ADJUVANTS FOR ANTI-RABIES VACCINES

Venkataswamy, M.M., Ph.D.^1^, Nagaraju, R., MS^1^

^1^Neurovirology, National Institute of Mental Health and Neurosciences, Bengaluru, 560078.

Rabies is a major public health problem in India and the current vaccination against rabies requires multiple doses for accomplishing protective titres of neutralizing antibodies. The need for several doses makes the immunization process challenging as well as expensive. The present study was designed to evaluate a novel NKT cell based glycolipid adjuvant with rabies vaccine for its ability to induce protective immune responses with a single dose. C57BL6 mice were vaccinated with either the vaccine alone or with the glycolipid adjuvanted vaccine and the efficacy was determined by analyzing the serum anti-rabies neutralizing antibodies using the Rapid Fluorescence Focus Inhibition Test (RFFIT) and antigen-specific T cell responses were assessed by the ELISPOT assay. Survival of the vaccinated mice was assessed against a challenge with the CVS strain of rabies. The glycolipid adjuvanted vaccine was observed to enhance the titres of neutralizing antibodies as well as the rabies specific T cell responses, in comparison to the vaccine alone. The adjuvant was also observed to potentiate better B cell responses in the regional lymph nodes following vaccination. Vaccine challenge experiments are in progress and the results will be presented. Supported by Department of Health Research – ICMR.

#### 17. CANNABIDIOL PRODUCES DISTINCT U-SHAPED DOSE-RESPONSE EFFECTS ON COCAINE-MOTIVATED BEHAVIOR AND ASSOCIATED RECRUITMENT OF PRELIMBIC NEURONS

Weiss, F., Ph.D.^1^, Nedelescu, H., Ph.D.^1^, Suto, N., Ph.D.^1^

^1^Department of Neuroscience, The Scripps Research Institute, La Jolla, CA 92130.

Cannabidiol (CBD) has received attention for the treatment of substance use disorders. CBD was shown to attenuate the motivating effects of several drugs of abuse, including cocaine. However, these effects have not been consistently effective. This inconsistency may be related to insufficient information on the spectrum of CBD dose effects. We addressed this issue by establishing a full dose-response profile of CBD’s actions using expression of cocaine-induced conditioned place preference as a model for drug-motivated behavior and by concurrently identifying dose-dependent effects of CBD on underlying neuronal activation and distinct neuronal phenotypes. Additionally, we established CBD levels in plasma and brain. CBD produced linear increases in CBD brain/plasma concentrations but suppressed conditioned place preference in a distinct U-shaped manner. In parallel with its behavioral effects, CBD produced U-shaped suppressant effects on neuronal activation in the prelimbic but not infralimbic cortex or nucleus accumbens. RNAscope *in situ* hybridization identified suppression of glutamatergic and GABAergic signaling in the prelimbic cortex as a possible cellular mechanism for the attenuation of cocaine-induced conditioned place preference by CBD. The findings extend previous evidence on the potential of CBD in preventing drug-motivated behavior. However, CBD’s dose-response profile may have important dosing implications for future clinical applications and may contribute to the understanding of discrepant CBD effects on drug seeking reported in the literature. Supported by National Institutes of Health: NIAAA (AA023648, T32AA007456) and NIDA (DA039821).

#### 18. SERUM BIOMARKERS OF LIVER FIBROSIS IDENTIFY GLOBUS PALLIDUS VULNERABILITY

Zahr, N., Ph.D.^1^, Pfefferbaum, A., MD^2^

^1^Psychiatry and Behavioral Sciences, Stanford University School of Medicine, Stanford, CA 94305

^2^Center for Health Science, SRI International, Menlo Park, CA 94025.

The CNS manifestation of chronic liver disease can include magnetic resonance (MR) signal hyperintensities in basal ganglia structures. Here, relations between liver (serum-derived fibrosis scores) and brain (regional T1-weighted signal intensities and volumes) integrity were evaluated in a sample of 457 individuals including those with alcohol use disorders (AUD), people living with human immunodeficiency virus (HIV), those comorbid for AUD and HIV, and healthy controls. Liver fibrosis was identified from cutoff scores as follows: aspartate aminotransferase to platelet ratio index (APRI)>0.7 in 9.4 % (n=43) of the cohort; fibrosis score (FIB4)>1.5 in 28.0 % (n=128) of the cohort; and non-alcoholic fatty liver disease fibrosis score (NFS)>−1.4 in 30.2 % (n=138) of the cohort. Presence of serum-derived liver fibrosis was associated with high signal intensities selective to basal ganglia (i.e., caudate, putamen, and pallidum) structures. Pallidal hyperintensities, however, explained a significant portion of the variance in APRI (25.0 %) and FIB4 (23.6 %) cutoff scores. Further, among the regions evaluated, only the globus pallidus showed a correlation between greater signal intensity and smaller volume (r=−0.44, p<0.0001). Finally, higher pallidal signal intensity correlated worse ataxia (eyes open *ρ*=−0.23, p = 0.0002; eyes closed *ρ*=−0.21, p=0.0005). This study suggests that clinically relevant, serum biomarkers of liver fibrosis such as the APRI may identify individuals vulnerable to globus pallidus pathology that may contribute to problems with postural balance. Supported by National Institute on Alcohol Abuse and Alcoholism (AA005965 and AA017347).

### TRAINEE POSTERS (T1–T28)

#### 1. ROLE OF PRMT1-PTX3 AXIS IN REGULATING FERRITINOPHAGY IN GLIOMA

Kirti ^1^, Gowda, P.^1^, Umdor, S.^1^, Patrick, S.^1^, Suri, V.^2^, Sen, E.^1^

^1^Division of Cellular and Molecular Neuroscience, National Brain Research Centre, Manesar, Gurugram, 122051

^2^Neurosciences Centre, All India Institute of Medical Sciences, New Delhi, 110029.

IDH1 (Isocitrate dehydrogenase 1) mutations are somatic mutations in gliomas that are associated with a better prognosis. The common point mutation (IDH1-R132H) affects the epigenetic landscape and elicits anti-proliferative signatures. However, the mechanism of cell death pathways (e.g., altered autophagic flux) in IDH1 mutant gliomas is not well elucidated. While altered histone methylation in IDH1-R132H gliomas is reported, there is a limited understanding of PRMT1, an arginine methyltransferase that regulates transcriptional activation of genes involved in gliomagenesis and cell death pathways by modulating its asymmetric dimethyl H4R3 epigenetic mark (H4R3me2a). Here we show that diminished PRMT1/H4R3me2a in IDH1-R132H epigenetically regulates the expression and release of inflammatory molecule PTX3 (Pentraxin 3) by affecting YY1 binding on its promoter. PTX3 is required for maintaining the autophagic flux in gliomas. PRMT1-PTX3 deficiency triggers ferritinophagy in IDH1-R132H cells as exhibited by altered levels of ferritin genes and defective iron storage. PRMT1 inhibition augments the ability of PTX3 to affect ferritinophagy-associated cell death negatively. This correlation of PRMT1 and PTX3 with ferritinophagic markers is conserved in gliomas of diverse genetic landscape and shows prognostic value in IDH1 mutant glioma patients. We demonstrate for the first time that PRMT1/H4R3me2a differentially regulates PTX3-driven ferritinophagic flux in IDH1 wild-type and mutant gliomas. Targeting this PRMT1-PTX3-ferritinophagy axis could be exploited for therapeutic gains. Supported by 1. Department of Biotechnology (DBT), Government of India. 2. Core funds from National Brain Research Centre, India.

#### 2. OPIOID-INDUCED MICROBIAL DYSBIOSIS DISRUPTS CPT-11 METABOLISM AND INCREASES GASTROINTESTINAL TOXICITY IN A MURINE MODEL

Abu, Y., BS ^1^, Meng, J., Ph.D.^1^, Zhang, Y., Ph.D.^1^, Yan, Y., Ph.D.^1^, Tao, J., Ph.D.^1^, Ramakrishnan, S., Ph.D.^1^, Chen, C., Ph.D.^2^, Zhou, Y., Ph.D.^2^, Xie, Y., BS^2^, Roy, S., Ph.D.^1^

^1^Department of Surgery, University of Miami, Miami, FL 33136

^2^Department of Food Science and Nutrition, University of Minnesota, Minneapolis, MN 55108.

Opioids are commonly used for the management of cancer-associated pain and chemotherapy-induced diarrhea. The chemotherapeutic agent CPT-11 causes severe GI toxicity as a result of deconjugation of its inactive metabolite SN-38G by intestinal bacterial β-glucuronidases to the pharmacologically active SN-38. As opioids are known to cause gut microbial dysbiosis, this study evaluated whether CPT-11’s anti-tumor efficacy and GI toxicity are exacerbated by opioid co-administration in a murine model. Gut microbiome analysis showed that morphine treatment induced enrichment of β-glucuronidase-producing bacteria in the intestines of CPT-11-treated mice, resulting in SN-38 accumulation and exacerbation of GI toxicity in the small intestine. Oral administration of both antibiotics and a glucuronidase inhibitor protected mice against GI toxicity induced with CPT-11 and morphine co-administration, implicating a microbiome-dependent mechanism. Additionally, morphine and loperamide decreased the plasma concentration of SN-38 and compromised CPT-11’s anti-tumor efficacy, although this seemed to be microbiome-independent. Together, our studies show that gut microbiota play a significant role in opioid and chemotherapeutic agent drug-drug interactions. Our studies further support that inhibition of gut microbial glucuronidase may also prevent adverse GI effects of CPT-11 in patients on opioids. Supported by F31DA053795, R01 DA050542, R01 DA047089, R01 DA043252, R01 DA044582, Sylvester Comprehensive Cancer Center.

#### 3. NEONATAL MORPHINE RESULTS IN LONG-LASTING ALTERATIONS TO THE GUT MICROBIOME IN ADOLESCENCE AND ADULTHOOD IN A MURINE MODEL

Antoine, D.^1^

^1^Department of Surgery, University of Miami, Miami, FL 33136.

Despite the many advancements in the field of pain management, the use of intravenous opioids, such as morphine, in neonates is still a challenge for clinicians and researchers, as the available evidence concerning the long-term consequences of such an early exposure is limited. In particular, little is known concerning the long-term consequences of neonatal morphine exposure on the gut microbiome, which has been identified as a key modulator of health and diseases. Consequently, the purpose of this study was to investigate those long-term consequences of neonatal morphine on the gut microbiome. Newborn mice were exposed to either morphine (5 mg/kg/day) or saline for a duration of 7 ± 2 days. Fecal samples were collected during adolescence and adulthood to longitudinally assess the gut microbiome. DNA extracted from the stool samples were sent out for 16s rRNA sequencing. During adolescence, neonatal morphine resulted in a significant increase of α -diversity and an overall decrease in the abundance of several commensal genera. During adulthood, β-diversity revealed a significantly different microbial composition of the neonatally morphine-exposed mice than that of the controls. The results demonstrate that morphine exposure during this critical developmental period resulted in long-lasting changes, particularly a reduction in several commensal bacteria. Supported by NIDA.

#### 4. LONG NON-CODING RNA MEDIATED CELLULAR REPROGRAMMING CAUSED BY ZIKV E PROTEIN IN HUMAN NEURAL PROGENITOR CELLS

Arora, H., MS ^1^, Prajapati, B., Ph.D.^2^, Seth, P., Ph.D.^1^

^1^Cellular and Molecular Neuroscience, National Brain Research Centre, Manesar, 122052

^2^Department of Medical Biochemistry and Cell Biology, Institute of Biomedicine, Sahlgrenska Academy, University of Gothenburg, Gothenburg, 40530.

Zika virus (ZIKV) infection during the first trimester of the pregnancy leads to Congenital zika syndrome in the neonates. Viral infection hampers fetal developmental and causes microcephaly. Human neural progenitor cells(hNPCs) play an important role in brain development. Innumerous evidences suggest high susceptibility of hNPCs towards ZIKV. In this study we aim to elucidate the molecular mechanisms that lead to cellular alterations upon ZIKV E protein transfection of hNPCs. We investigate various properties of hNPCs: proliferation, differentiation, migration and inflammation, alterations in which could impair neurogenesis and lead to microcephaly. In our study we found that ZIKV E protein transfection leads to cell cycle arrest, decrease in proliferation and increase in mitotic length of the dividing cells. We found CyclinD1 and its upstream molecules of the pathway to be dysregulated. Intracellular calcium at basal level as well as upon ATP stimulation has been studied and found to be distorted. ZIKV E protein transfected cells also showed pre-mature differentiation as pro neural genes were found to be up-regulated. ZIKV E protein is found to disrupts migrational properties of hNPCS and cause inflammation. We also explored the possible involvement of long non coding RNAs in ZIKV caused neuropathogenesis. We found few lncRNAs to be differentially expressed upon E protein transfection of hNPCs. Employing bioinformatic tools it can be said that these lncRNAs play an important role in regulation of viral life cycle, host’s defense response and cell proliferation. Supported by NBRC core funds.

#### 5. SYNERGISTIC EFFECTS OF ALCOHOL USE AND COVID-19 IN THE DEVELOPMENT OF CHRONIC PAIN

Bishir, M., MS ^1^, Chan, M.-H., Ph.D.^2^, Chidambaram, S.B., Ph.D.^3^, Chang, S.L., Ph.D.^1^

^1^Institute of NeuroImmune Pharmacology, Seton Hall University, South Orange, NJ 07079

^2^Institute of Neuroscience, National Chengchi University, Taipei, 11605

^3^Department of Pharmacology, JSS College of Pharmacy, JSSAHER, Mysuru, 570015.

Case reports showed that significant population of COVID-19 patients develop severe neuropathic pain upon SARS-CoV-2 infection. The exact molecular mechanisms underlying onset and progression of neuropathic pain during COVID-19 require further investigation. In a preliminary study, we found 163 commonly shared genes between COVID-19 and pain, suggesting that molecules associated with COVID-19 may be involved in the modulation of pain sensation. Alcohol misuse has been shown to exacerbate the infections by SARS-CoV-2 infection leading to increased COVID-19 mortality. Reports have suggested that acute alcohol consumption produce analgesic effects, however chronic alcohol consumption results in chronic pain and hyperalgesia. In addition, we and others have reported that systemic inflammation induced by alcohol can trigger neuroinflammation in the CNS, which can also be a potential contributor to alcohol induced chronic pain. With these premises, we have hypothesized that alcohol consumption and COVID-19 synergistically trigger chronic pain including inflammatory and neuropathic pain. We will present the molecular mechanisms and pathways underlying how alcohol use and COVID-19 exacerbate chronic pain by employing QIAGEN Knowledge Base repository data, Ingenuity Pathway Analysis bioinformatics tools and Genomic Workbench CLC22. Supported by National Institutes of Health grant AA025964 and AA029925.

#### 6. COVID-19 IS ASSOCIATED WITH BYSTANDER POLYCLONAL AUTOREACTIVE B CELL ACTIVATION, BUT NOT LINKED TO DISEASE SEVERITY

Johnson, D., MS ^1^, Gisslén, M., MD^3^, Jiang, W., MD^1^

^1^Department of Microbiology and Immunology, Medical University of South Carolina, Charleston, SC 29425

^2^Ralph H. Johnson VA Medical Center, Charleston, SC 29425

^3^Department of Infectious Diseases, Institute of Biomedicine, Sahlgrenska Academy, University of Gothenburg, Gothenburg, 41645

^4^Region Västra Götaland-Department of Infectious Diseases, Sahlgrenska University Hospital, Gothenburg

^5^Division of Infectious Diseases, Department of Medicine, Medical University of South Carolina, Charleston, SC 29425.

Coronavirus disease 2019 (COVID-19) is associated with autoimmune features and autoantibody production in a small subset of the population. Pre-existing neutralizing antitype I interferon (IFN) autoantibodies are related to the severity of COVID-19. Plasma levels of IgG and IgM against 12 viral antigens and 103 self-antigens were evaluated using an antibody protein array in patients with severe/critical (n=8) or mild/moderate (n=7) COVID-19 disease and uninfected controls (n=7). Patients exhibited increased IgGs against Severe acute respiratory syndrome coronavirus-2 (SARS-CoV-2) proteins compared to controls (p<0.05, Kruskal- Wallis test), but no difference was observed between the two patient groups (p>0.05). 78 % autoreactive IgGs and 93 % autoreactive IgMs were increased in patients versus controls. There was no difference in the plasma levels of anti-type I IFN autoantibodies or neutralizing anti-type I IFN activity of plasma samples from the two patient groups (p>0.05). Increased anti-type I IFN IgGs were correlated with higher lymphocyte accounts, suggesting a role of nonpathogenic autoantibodies (p<0.05, Spearman correlation tests). Notably, among the 115 antibodies tested, only plasma levels of IgGs against human coronavirus (HCOV)-229E and HCOV-NL63 spike proteins were associated with mild disease (p<0.05). COVID-19 was associated with a bystander polyclonal autoreactive B cell activation, but none of the autoantibody levels were linked to disease severity. Understanding the mechanism of life-threatening COVID-19 is critical to reducing mortality and morbidity. Supported by Ralph H. Johnson VA Medical Center Merit Review Award Number CX002422.

#### 7. OPIOIDS MODULATE MICROGLIA ACTIVATION AND CNS SIV VIRAL DYNAMICS

Johnson, S.D., Ph.D.^1^, Olwenyi, O.A., Ph.D.^2^, Bidokhti, M., Ph.D.^1^, Pandey, K., MS^2^, Uppada, J., Ph.D.^1^, Buch, S., Ph.D.^1^, Byrareddy, S.N., Ph.D.^1^

^1^Department of Pharmacology and Experimental Neuroscience, University of Nebraska Medical Center, Omaha, NE 68106

^2^Department of Pathology and Microbiology, University of Nebraska Medical Center, Omaha, NE 68106.

Substance abuse is a persistent hurdle in HIV eradication, due to increased transmission and significant neurocognitive and behavioral changes associated with dependence. Further, little is known about viral reservoir establishment in the central nervous system (CNS) during opioid use. We hypothesized that opioid exposure exacerbates neuroimmune dysfunction and increases CNS viral reservoir seeding thereby potentiating negative impacts of infection. To test this, rhesus macaques (RMs) were administered morphine (6 mg/kg IM 2×/day) or saline and infected with SIV for 6–8 months. RMs were sacrificed, CNS dissected, myeloid cells isolated for flow cytometry, and viral loads determined. Consistent with our hypothesis, trends were found across brain regions in increased surface Fcg receptor CD32 and CD64 on microglia (CD45int CD11B+) of morphine-treated RMs compared with controls, and decreased FcgR CD16. Further, increased CD14 expression was found in all regions of morphine-exposed RMs with differences most pronounced in the hippocampus. Such changes are consistent with elevated microglia reactivity and therefore increased HIV-associated neurocognitive disorder risk. However, minimal differences were found in CNS viral loads between groups. Recently, we validated these findings in an SHIV model finding higher CD14 expression and differential dysregulation of FcgRs. Together, these data highlight the reinforcement of neuroimmune dysregulation by opioid abuse during HIV infection and suggest novel therapeutic targets for aberrant microglia activation and reservoir persistence. Supported by MH113455/DAA041751/DA043164.

#### 8. HIV-1 TAT-MEDIATED MICROGLIAL FERROPTOSIS INVOLVES THE ACSL4/MIR204-5P SIGNALING AXIS.

Kannan, M., Ph.D.^1^, Periyasamy, P., Ph.D.^1^, Buch, S.^1^

^1^Department of Pharmacology and Experimental Neuroscience, University of Nebraska Medical Center, Omaha, NE 68198.

Globally, ∼38.4 million people are living with HIV. The advent of combination antiretroviral therapy has increased the life expectancy of people living with HIV-1. However, about 50 % of them develop neurological impairments, termed HIV-associated neurocognitive disorders, but the mechanisms underlying its pathogenesis remain unclear. In this study, we demonstrated the dysregulation of cellular iron metabolism increases the labile iron pool that triggers ferroptosis. Our results showed that exposure of mouse primary microglial cells to HIV-1 Tat resulted in an induction of ferroptosis, which was characterized by increased levels of Acyl-CoA Synthetase Long-Chain Family Member 4 (ACSL4), lipid peroxidation, the labile pool of iron, ferritin heavy chain-1, mitochondrial dysfunction, elevated pro-inflammatory cytokines with a concomitant decrease in glutathione peroxidase-4 levels. Both pharmacological inhibitors (ferrostatin-1 and deferoxamine) and gene silencing approach using ACSL4 siRNA further validated the critical role of ferroptosis in HIV-1 Tat-mediated neuroinflammation. Further, we identified miR-204 as an upstream modulator of ACSL4, which was further confirmed by argonaute immunoprecipitation, dual luciferase assay, *in situ* hybridization, and miR-204-5p overexpression. These *in vitro* findings were also validated in the prefrontal cortices, striatum, and hippocampus of HIV-1 transgenic rats, *in vivo*. Overall, this study underscores the novel role of miR-204/ACSL4-mediated ferroptosis in microglial activation and neuroinflammation in the context of HIV-1 Tat.

#### 9. ZIKA VIRUS ENVELOPE PROTEIN MODULATES THE PROPERTIES AND INTEGRITY OF IN-VITRO HUMAN BLOOD BRAIN BARRIER

Kaur, G., MS ^1^, Pant, P., MS^1^, Bhagat, R., Ph.D.^2^, Seth, P., Ph.D.^1^

^1^Cellular and Molecular Neuroscience, National Brain Research Centre, Manesar, 122052

^2^Department of Psychiatry, Washington University in Saint Louis, Saint Louis, MO 63130.

The blood-brain barrier (BBB) is a complex and well-regulated entity between the peripheral circulation and central nervous system (CNS) and helps in maintaining the homeostasis of the CNS. The presence of BBB protects the CNS from viral infections but many neurotropic viruses can access the brain and establish CNS infection. Zika virus (ZIKV) is a mosquito-borne Flavivirus, known for worldwide outbreaks (2015–2016) causing neonatal microcephaly, and encephalitis. The surface envelope (E) protein of ZIKV is involved in host cell binding and membrane fusion, thereby facilitating viral entry. In our present study, we explored the effects of ZIKV E protein on the properties of BBB cells-human brain microvascular endothelial cells (hBMECs) and human progenitor-derived astrocytes, using an *in vitro* BBB model. We used transwell apparatus to recapitulate the *in vivo* microenvironment, making our model physiologically relevant. We found exposure to E protein resulted in the activation of hBMECs and astrocytes. E protein resulted in reduced expression of characteristic endothelial tight junction proteins resulting in a modulated state of BBB integrity and permeability. Increased levels of inflammatory cytokines and chemokines in hBMECs also influenced BBB integrity. ZIKV E protein further induced astrogliosis (reactive astrocytes) as shown by increased levels of GFAP and Vimentin and dysregulated extracellular glutamate levels. Our findings suggest that BBB cells are highly vulnerable to ZIKV infection through exposure to ZIKV E protein, resulting in altered BBB integrity. Supported by DBT grant and NBRC Core.

#### 10. STAT1 INHIBITORS AS POSSIBLE DRUG TARGETS FOR REDUCING THE ONCOGENIC EFFECTS OF FAT1

Khan, M.T., MS ^1^, Jalota, A., Ph.D.^1^, Chosdol, K., MD, Ph.D.^2^, Sinha, S., MD, Ph.D.^2^

^1^Molecular Neuroscience, National Brain Research Centre, Gurgaon, 122052

^2^Department of Biochemistry, All India Institute of Medical Science, New Delhi, 110029.

Gliomas are the most common, heterogeneous primary central nervous system tumours with very poor prognosis. FAT1 has shown oncogenic effect in glioma by down-regulating the tumour suppressor gene PDCD4, which leads to activation of AP1 Transcriptional activity thereby increasing the EMT and inflammatory microenvironment of tumour Cells. We have earlier shown that knock down of FAT1 led to increased PDCD4 level and repression of AP1 activity in glioma cells. In this study we show that the transcription factor STAT1 binds to the PDCD4 promoter region using in silico analysis. Knock down of FAT1 *in vitro* has also resulted in the reduced expression of STAT1 protein in glioblastoma cell line signifying positive correlation between FAT1 and STAT1. Reduction of STAT1 in glioblastoma cell lines by knock down experiment significantly increased PDCD4 expression and decreased PDCD4 targets in Glioblastoma cell lines. Targeting STAT1 through FDA approved drugs could possibly rescue the oncogenic effect of FAT1 in glioma mediated by FAT1 -PDCD4 axis. Supported by DBT NBRC core grant, J C Bose fellowship.

#### 11. NANOMEDICINE TECHNOLOGIES TO TACKLE NEUROHIV AND ASSOCIATED NEUROINFLAMMATION

Kolishetti, Nagesh^1^

^1^Department of Immunology and Nanomedicine, Florida International University, Miami, FL 33199.

Tackling reservoirs of Human Immunodeficiency Virus (HIV) remains a challenging task even after the significant success of combination antiretroviral therapy (cART). This is mainly due to the difficulty to clear the virus from its reservoirs in difficult-to-reach locations in body such as the brain. Both the HIV and the cART are known to increase the inflammation in the affected region, which often results in HIV-associated neurodegenerative diseases in the brain, and methods to tackle these are urgently needed. This work aims to develop nanotechnology approaches to deliver antiretroviral and anti-inflammatory therapies at one of these difficult-to-reach location, namely, brain by using technologies that will cross the blood-brain barrier. We will discuss two different nanomedicine approaches in this work: One approach uses magneto-electric nanoparticle technology, which utilities external magnetic field to direct the formulation to the brain and the electric field to deliver the drug at this target location and in the second approach we will use targeted polymeric nanoparticle-based approach to deliver the drugs to the brain. Our results indicate both the technologies successfully deliver the therapeutics across the blood-brain barrier and tackle the HIV and associated neuroinflammation. Supported by FIU pilot grant.

#### 12. MULTI-OMICS ANALYSIS REVEALING THE INTERPLAY BETWEEN GUT MICROBIOME AND THE HOST FOLLOWING OPIOID USE

Kolli, U.K., Ph.D. ^1^, Jalodia, R.J., Ph.D.^1^, Moidunny, S.M., Ph.D.^1^, Singh, P.S., Ph.D.^1^, Ban, Y.B., Ph.D.^1^, Tao, J.T., Ph.D.^1^, Cantu, G.C., MD^1^, Valdes, E.V., MS^1^, Ramakrishnan, S.R., Ph.D.^1^, Roy, S.R., Ph.D.^1^

^1^Department of Surgery, University of Miami, Miami, FL 33136.

Opioid use associated microbial dysbiosis is a key regulator of intestinal homeostasis and behavioral responses to opioid. For a mechanistic understanding of opioid-induced gut dysbiosis on intestinal homeostasis, we comprehensively studied and identified key changes in microbiome using whole genome sequencing, metabolites using untargeted metabolomics, and host gene expression changes using RNA sequencing. We identified the role of gut microbiome in mediating signature gene changes in innate and adaptive immune response as well gut barrier dysfunction by using opioid-use and antibiotics treated opioid-use models. Integrative analysis of multi-omics data revealed the bi-directional host-microbiome interactions. Importantly, we highlighted the relationship between microbiota, and changes in riboflavin, flavonoids and fatty acids including phosphocholines, carnitines, bile acids, and ethanolamines with the gene expression changes involved in inflammation and barrier integrity of intestine. This work identified potential therapeutic interventions to limit microbial dysbiosis and presents a unique resource to the opioid research community. Supported by National Institutes of Health Grants (R01 DA050542, R01 DA047089, R01 DA044582, R01 DA043252, R01 DA037843 and R01 DA034582).

#### 13. POTENTIAL MECHANISMS THAT UNDERLIE THE SYNERGISTIC EFFECTS OF METHAMPHETAMINE (METH) AND SARS-COV-2 INDUCED MITOCHONDRIAL DYSFUNCTION IN HUMAN MICROGLIA

Machhar, J.S., MS ^1^, Abbasi, S., Ph.D.^2^, Khoo, T.C., MS^2^, Aalinkeel, R., Ph.D.^1^, Khmaladze, A., Ph.D.^2^, Mahajan, S.D., Ph.D.^1^

^1^Department of Medicine, Division of Allergy, Immunology, and Rheumatology, Jacobs School of Medicine & Biomedical Sciences, University at Buffalo, Buffalo, NY 14203.

^2^Physics Department, University at Albany SUNY, Albany, NY 12222.

SARS-COV-2 is a neurotropic virus that causes multiple neuropathologies known as Neuro-COVID. According to our previous studies, SAR-COV-2 induces mitochondrial dysfunction and activates mitochondrial-dependent intrinsic apoptotic pathways, resulting in neuronal apoptosis and neuropathologies. Furthermore, SARS-COV-2 alters mitochondrial lipidome, resulting in altered membrane structure and higher levels of reactive oxygen species (ROS). Microglia are the immune cells of the CNS and mediate homeostasis, during viral neurotropic infections. METH is an addictive stimulant with neurotoxic effects that aggravate SARS-COV-2 related neuropathogenesis symptoms. Thus, our goal is examining the synergistic effect of SARS-COV-2 and METH on mitochondrial function. We speculate that exposure of microglia to METH +/− SARS-COV2 treatment resulting in mitochondrial dysfunction leads to accumulation of mtDNA, altered mitochondrial membrane potential, increased oxidative stress, inflammation and lipid peroxidation. Through METH +/− SARS-COV-2, we will investigate changes in mtDNA biomarkers of mitochondrial dysfunction, mitochondrial membrane potential, ROS levels, pro-inflammatory cytokines, and lipid-peroxide species using validated in-house assays as well as commercial kits. The infection of METH abusing subjects with SARS-CoV-2 may result in extended neurological deficits and our studies will enable the identification of novel therapeutic targets able to limit neuroinflammatory responses by altering lipid synthesis, modulating lipid transport, and reducing mitochondrial oxidative stress. Supported by NIH- National Institute of Drug Abuse (Grant # 5R01DA047410-02) to SM & the Office of Vice President for Research and Economic.

#### 14. CHARACTERIZATION OF CONFORMALLY COATED SPINAL PROGENITOR CELLS

Marin, B.M., BS ^1^, Gonzalez, G.G., BS^1^, Al-Hanoosh, F.A., BS^1^, Tomei, A.T., Ph.D.^1^, Dumont, C.D., Ph.D.^1^

^1^Biomedical Engineering, University of Miami, Miami, FL 33136.

Traumatic spinal cord injury (SCI) is a devastating condition that disrupts autonomic, sensory, and motor function. Spinal progenitor cells (SPCs) can modulate the site into a more pro-regenerative milieu, but poor SPC survival due to inflammation can hamper transplant potential. Thin layered biomaterial encapsulation, termed conformal coating, is a technique that shields transplants from immune attack while allowing diffusion of nutrients and waste. Conformal coating reduces diffusion distances and minimizes transplant volume size compared to traditional encapsulations. Conformal coating has not been applied to SPCs as an SCI therapy, however, the objective of this study is to engineer a conformal coating capable of maintaining SPC survival and potency in inflammatory models. SPCs were expanded as neurospheres and encapsulated in a polyethylene glycol (PEG) capsule using a microfluidic platform. SPC viability, proliferation, SPC phenotype, and capsule integrity were evaluated over 14 days *in vitro*. Immunostaining and qRT-PCR were used to evaluate SPC outcomes. RNA was isolated from cell cultures to identify and quantify expression of specific marker proteins. Secreted markers NGF, PGE2, VEGF, GDNF, and FGF were assessed by western blot (WB) analysis. Conformally coated neurospheres can be encapsulated without negatively impacting cell viability and sustained over a 2-week period. Future experiments will interrogate SPC secretory anti-inflammatory potential.

#### 15. EFFICACIOUS EXCISION OF HIV-1 MRNA FROM MIXED GLIA IN VITRO

McLaurin, K.A., Ph.D. ^1^, Li, H., MD, Ph.D.^1^, Mactutus, C.F., Ph.D.^1^, Booze, R.M., Ph.D.^1^

^1^Cognitive and Neural Science Program, University of South Carolina, Columbia, SC 29208.

Mixed glia are infiltrated with HIV-1 virus early in the course of infection leading to the development of a persistent viral reservoir in the central nervous system. Modification of the HIV-1 genome using gene editing techniques, including CRISPR/Cas9, has shown great promise towards eliminating HIV-1 viral reservoirs; whether these techniques are capable of removing HIV-1 viral proteins from mixed glia, however, has not been systematically evaluated. Herein, the efficacy of adeno-associated virus 9 (AAV9)-CRISPR/Cas9 gene editing for eliminating HIV-1 mRNA from cortical mixed glia was evaluated. Cell culture techniques were utilized to isolate and grow sex-specified mixed glia from neonatal (Postnatal Day (PD) 1 to PD 3; n=8; Male: n=4, Female: n=4) HIV-1 Tg rats. A within-subjects experimental design was utilized to treat HIV-1 Tg mixed glia with varying doses (0, 0.9, 1.8, 2.7, 3.6, 4.5, or 5.4 μL) of CRISPR/Cas9 for 72 h. Expression of HIV-1 mRNA was evaluated using *in situ* hybridization. HIV-1 mRNA, independent of biological sex, were abundantly expressed in the absence of CRISPR/Cas9 treatment (i.e., 0 μL). Excision efficiency of at least 40 % was observed in cells grown from five neonatal HIV-1 Tg pups, whereby two pups exhibited significant excision at low doses (i.e., 1.8 μL) and three pups displayed pronounced excision at high doses (i.e., 5.4 μL). Collectively, these proof-of-concept observations support the susceptibility of mixed glia to efficacious gene editing via AAV9-CRISPR/Cas9. Supported by NIH Grants: DA013137, DA056288, MH106392, NS100624.

#### 16. IMPLICATIONS OF SARS-COV-2 ON HUMAN NEURONS

Mehta, A., Ph.D. ^1^, Chaudhuri, R., Ph.D.^1^, Seth, P., Ph.D.^1^

^1^Department of cellular and molecular neuroscience, National Brain Research Centre, Gurugram, 122051.

SARS-CoV-2 presents a diverse disease profile including mild, moderate and severe neurological complications in up to 60 % of Long COVID-19 patients. The virus can give rise to these neurological complications either by direct infection causing the death of neurons or by the neuroinflammatory response of the immune system. There are multiple lines of evidence for direct infection and death of neurons in 2D neuronal cultures, 3D brain organoid models and brain autopsy studies. Nevertheless, there is no study that has explored the exact molecular mechanisms involved in SARS-CoV-2-mediated neuronal death. We have screened individual viral proteins for cell death and have identified Orf6 as the protein causing maximum cell death. In continuity, we identified that necroptosis is the predominant cell death pathway that is induced in the fetal neural progenitor-derived neurons expressing Orf6. These findings were later validated in post-mortem brain tissue sections of COVID-19 patients. We found that this viral protein interacts with the host’s mitochondrial protein, leading to mitochondrial dysfunction by altering mitochondrial membrane permeability, fission/fusion machinery and levels of mitochondrial reactive oxygen species. Our work sheds light on previously unknown mechanisms by which neuronal death occurs in COVID-19 patients. We are currently exploring the pharmacological modulations of these cells using molecules for therapeutic intervention. Supported by DST and NBRC.

#### 17. REGULATION OF MICROGLIA-MEDIATED NEUROINFLAMMATION BY LNCRNA GM20559 UPON FLAVIVIRUS INFECTION

Mohapatra, S., MS ^1^

^1^Molecular Neuroscience, National Brain Research Centre, Gurugram, 122051.

Long non-coding RNA (lncRNA) are a group of RNA molecules which are longer than 200 nucleotides in size. Research has shown that the expression of many lncRNAs is misregulated in case of various diseases. We wanted to learn about the lncRNA profile in Japanese Encephalitis infection of microglia, to achieve that RNA sequencing of JEV-infected N9 microglial cell sample and uninfected cells was done. Sequencing revealed dysregulated expression of many lncRNAs due to the infection. Gm20559, one such lncRNA showed a considerable amount of fold change in infected cells. Increase in expression of Gm20559 in JEV-infected N9 was also associated with an upregulation of many proinflammatory cytokines (IFN-α, IFN-β, IFN-γ, IL-6, IL-1β, IP-10), chemokines (MCP-1), interferon stimulated genes (ISG) (RIG-1, OAS-2) as well as viral replication as checked by qPCR. Similar results were also obtained in case of West Nile Virus (WNV) infected-N9 cells. Upon inhibiting Gm20559 there was a sharp reduction in the expression of proinflammatory cytokines, chemokines and ISGs whereas viral replication remains unchanged in both JEV and WNV-infected N9 microglia cells. Expression of RIG-I was also downregulated in Gm20559 inhibition samples compared to control in protein level in microglial cells. More experiments are required to decipher the role of Gm20559 in modulating inflammation in case of both JEV and WNV infection. Supported by J.C. Bose Fellowship to AB (SERB) (JCB/2020/00037).

#### 18. DOWNREGULATION OF PTEN SUPPRESSES PATHOGENESIS OF HUMAN NEURONAL POLY(Q) IN DROSOPHILA BY REGULATING MITOCHONDRIAL DYSFUNCTION

Nisha, Ph.D.^1^

^1^National Brain Research Centre, Manesar, Gurugram, HI 122052.

Huntington’s disease (HD) is a dominant late-onset genetic disorder that is primarily characterized by an abnormal expansion of CAG repeats within the N terminus of the huntingtin (Htt) protein. These CAG expanded repeats lead to the formation of aggregated proteins (inclusion bodies) which results in the progressive loss of neurons in the cerebellum, basal ganglia, and cortex in the adult human brain. Similarly, overexpression of the human form of mutant HTT protein with 138 poly(Q) repeats leads to the formation of inclusion bodies and increases cellular toxicity in the Drosophila eye. With the aim to find a novel genetic modifier of poly(Q) disorders that could also be utilized as a potential drug target, the Drosophila PTEN (Phosphatase and tensin homolog) was identified as a potential suppressor of poly(Q)-mediated neurotoxicity and degeneration. We demonstrate for the first time that targeted downregulation of PTEN could effectively mitigate poly(Q)-mediated neurodegeneration in fly models. We demonstrated that targeted knockdown of PTEN causes a substantial reduction in poly(Q) aggregate formation. Strikingly, RNAi-mediated downregulation of PTEN also restricts the cellular level of apoptosis as well as axonal branching defects. There also appeared a possibility of improved mitochondrial biogenesis mediated rescue of poly(Q) phenotype due to downregulation of PTEN. Therefore, our study strongly suggests that modulation of the PTEN (PI3K/AKT) pathway could be an effective therapeutic intervention against poly(Q) disorders.

#### 19. ISCHEMIC STROKE RECOVERY IS AFFECTED BY CEREBRAL AMYLOID ANGIOPATHY

Osborne, O., MS ^1^, Daftari, M.^1^, Naranjo, O., BS^1^, Drexler, G.^1^, Lewis, E., BS^1^, Dykxhoorn, D., Ph.D.^2^, Toborek, M., MD, Ph.D.^1^

^1^Department of Biochemistry and Molecular Biology, University of Miami Miller School of Medicine, Miami, FL 33136.

^2^Department of Human Genetics, University of Miami Miller School of Medicine, Miami, FL 33136.

Recovery after ischemic stroke is one of the most critical rehabilitative medical problems in medicine, with as many as two-thirds of stroke survivors requiring neurorehabilitation. Furthermore, post-stroke outcomes have been poorly studied in patients with cerebral amyloid angiopathy (CAA). CAA is a form of cerebrovascular disease and is characterized by substantial beta-amyloid (Aβ) accumulation within cerebral vasculature, including the blood-brain barrier (BBB). The integrity of vessel wall architecture is crucial for post-stroke tissue recovery since many proliferative neural progenitor cells (NPC) are close to and communicating with cerebral vasculature (BBB). This pool of proliferative NPCs that can become depleted by stroke can be mutually affected by Aβ accumulation, leading to delayed recovery of both motor and cognitive functions. We aim to understand the mechanism behind delayed post-stroke recovery because of both conditions so that targeted therapies can be proposed in the future. To study this, we employed a transgenic 5xFAD mouse model to analyze CAA and induced cerebrovascular ischemia. After induction of MCAO, mice were analyzed for cognitive behavior, neuroscore, and infarct sizes. Additionally, IHC and protein quantification displayed increased inflammatory responses and dysfunctional BBB signaling in mice with stroke and Aβ burden. Our results indicate that Aβ accumulation in cerebral vasculature exacerbates ischemic stroke outcomes and delays post-stroke recovery by inducing BBB dysfunction and aberrant neurogenesis of NPCs. Supported by NIH R01MH072567 F31NS125905.

#### 20. ECOHIV INFECTED CHME5 AS A NOVEL IN VITRO MODEL FOR HIV INFECTION AND LATENCY IN MICROGLIA

Ostermeier, B., BS ^1^, Maggirwar, S.B., Ph.D.^1^

^1^Microbiology, Immunology, and Tropical Medicine, The George Washington University, Washington, DC 20052.

Human immunodeficiency virus (HIV), is a retrovirus that can infect multiple reservoirs in the body, including microglial cells within the central nervous system (CNS). HIV infection in microglia likely assumes a state of latency, in which HIV is transcriptionally silent in the host genome. Studying CNS resident cells *in vitro* can be difficult since patient samples are not readily available. There are multiple models for studying HIV infection and latency in microglia *in vitro*. Human induced pluripotent stem cell-derived microglia and primary microglia are great HIV infection models, but they are expensive and non-dividing, thus limiting sample pool sizes. While immortalized (human and mouse) and monocyte-derived microglia models are less expensive alternatives, they lack initial robust HIV infection due to the increased presence of SAMHD1, which is known to be inhibited by SIV-derived Vpx. In this study, we propose a novel *in vitro* immortalized microglial cell model of HIV infection, EcoHIV-infected CHME5 (rat) cells that are primed with the Vpx-containing virus-like particles (VLPs). We utilized flow cytometry, imaging, and western blots, to develop a model of both infection and latency. Latency was confirmed using a range of latency reversing agents (LRAs). Our novel HIV infection model has shown robust infection rates, latency, and reversal of latency. With this model, researchers can readily investigate new pathways that influence HIV infection and latency in microglia, which will inform future HIV treatment and cure strategies.

#### 21. NOVEL ACE-2-INTERACTING DOMAIN OF SARS-COV-2 (AIDS) PEPTIDE INHIBITS SPIKE S1 INDUCED BEHAVIORAL AND NEURONAL INFLAMMATION IN VITRO AND IN VIVO MODELS.

Paidi, R.K., Ph.D.^1^, Pahan, K. Ph.D.^1^

^1^Neurological Sciences, Rush University Medical Center, Chicago IL 60612.

Covid-19 patients are suffered from various brain complications, but the underlying mechanism is not clear. Fatigue and cognitive impairment are amongst the most common and debilitating symptoms of post-COVID-19 syndrome. So far, there is no proper therapeutic strategy against Covid-19 and its implications on the brain. Since SARS-CoV-2 binds to angiotensin-converting enzyme 2 (ACE2) for entering into host cells, to target COVID-19 from a therapeutic angle, we designed a hexapeptide corresponding to the ACE2-interacting domain of SARS-CoV-2 (AIDS) that inhibits the association between receptor-binding domain-containing spike S1 and ACE-2. Initially, we validated the effect of AIDS peptide and its role in Spike S1-associated neuroinflammatory alteration in mouse primary glia cultures. For *in vivo*, recombinant spike S1 was administered intranasally for 14 days. Wild type and mutant AIDS peptide were administered post 5 days of treatment of spike S1. Post two weeks of treatment, we performed several behavioral assessments and biochemical evaluations. Elevated INOS levels and microglia activation were observed by Spike S1 treatment in mice primary microglia. AIDS peptide prominently attenuates Spike S1-associated inflammatory markers in glia cultures. Intranasal intoxication of C57/Bl6 mice with recombinant SARS-CoV-2 spike S1 led to fever, an increase in IL-6 in the lungs as well as in the brain, and impairment in spatial learning memory functions and motor disabilities, mimicking some of the important symptoms of Covid-19. Instranasal treatment with wtAIDS, but not mAIDS, pept. Supported by AG050432, AT010980, and NS108025 from NIH.

#### 22. DIFFERENTIAL IMMUNOPATHOGENESIS OF DELTA AND OMICRON VARIANTS OF SARS-COV-2 IN GOLDEN SYRIAN HAMSTERS

Pandey, Kabita, MS ^1^, Rajaiah, R.R., Ph.D.^1^, Kumar, N.K., Ph.D.^1^, Ambikan, A.A.^3^, Guda, R.S.^1^, Cohen, S.C.^2^, Neogi, U.N.^3^, Byrareddy, S.N.B., Ph.D.^1^

^1^Pharmacology and Exp Neuroscience, University of Nebraska Medical Center, OMAHA, NE 68105

^2^Department of Pathology and Microbiology, University of Nebraska Medical Center, OMAHA, NE 68105

^3^Department of Laboratory Medicine, Karolinska Institute, Salona

^4^Department of Genetics, Cell Biology and Anatomy, University of Nebraska Medical Center, OMAHA, NE 68105

^5^Department of Biochemistry and Molecular Biology, University of Nebraska Medical Center, OMAHA, NE 68105

COVID-19 pandemic is caused by SARS-CoV-2, resulting in millions of deaths worldwide. Due to the high mutation rate of SARS-CoV-2, highly transmissible and pathogenic variants have created infection waves globally. Therefore, understanding pathogenic mechanisms and host immune response underlying infection with SARS-CoV-2 and its variants of concern is of great interest. In this study, we evaluated the immunopathogenesis of two dominant SARS-CoV-2 variants *in vivo* using Golden Syrian Hamsters (GSH). Male GSHs (6–8 weeks age) were inoculated intranasally with B.1.617.2 (Delta) or B.1.1.529 (Omicron) lineages and virus shedding, viral load and metabolic changes in the target organs were assessed and compared. Our results demonstrated that Delta virus is more pathogenic compared to Omicron in GSH with relatively higher virus titers in throat swabs, the brain, and respiratory organs. Interestingly, GSH infected with the Omicron variant exhibited less lung damage than Delta-infected GSH lungs. The transcription levels of cytokines, chemokines, and interferon-stimulating genes (ISGs) in lungs and the brain of Delta-infected hamsters were significantly higher as compared to the Omicron-infected tissues. Further, lungs from GSH infected with Delta variant exhibited increased expression of metabolic enzymes compared to Omicron-infected lungs. Collectively, our data provide an insight into a comparative immunopathogenesis in biologically relevant animal model and offer new therapeutic targets to develop potent therapeutics against SASR-CoV-2 and its variants of concern. Supported by SNB acknowledges independent research and development (IRAD) funding from the NSRI and NRI.

#### 23. CANNABIDIOL (CBD) M PEG-PLGA NANOPARTICLE FOR TREATING NEUROPATHIC PAIN IN CHRONIC CONSTRICTION INJURY RAT MODEL

Qayum, S., MS ^1^, Schmitt, R., Ph.D.^2^, Garg, S., Ph.D.^2^, Mojdeganlou, H., MD^3^, Aalinkeel, R., Ph.D.^1^, Schwartz, S.A., MD, Ph.D.^1^, Prasad, P.N., Ph.D.^2^, Ignatowski, T., MD^3^, Mahajan, S.D., Ph.D.^1^

^1^Department of Medicine, Division of Allergy, Immunology, and Rheumatology, Jacobs School of Medicine and Biomedical Sciences/University at Buffalo, Buffalo, NY 14211.

^2^Department of Chemistry, Institute for Lasers, Photonics and Biophotonics/University at Buffalo, Buffalo, NY 14211

^3^Department of Pathology & Anatomical Sciences, Jacobs School of Medicine & Biomedical Sciences/University at Buffalo, Buffalo, NY 14211

Neuropathic pain is defined as ‘pain caused by a lesion or disease of the somatosensory nervous system’ and is challenging to treat. Cannabidiol (CBD) significantly suppresses chronic pain without causing apparent analgesic tolerance in rodents. CBD is emerging as a potential neurotherapeutic drug; however, its therapeutic utility is curtailed due to its reduced bioavailability and quick clearance which limits its effectiveness in the brain. Our goal is to develop a novel mPEG-PLGA nanoparticle-based CBD nanoformulation that will enhance its bioavailability and efficacy. To test the efficacy of the CBD nanoformulation, we are using the Chronic Constriction Injury (CCI) model where 4 ligatures are loosely placed around the sciatic nerve to induce neuropathic pain in 7–8-week-old male Sprague-Dawley rats. Thermal hyperalgesia and mechanical allodynia are assessed prior to surgery and every other day following surgery, including on the day of euthanasia-day 10. Animals are administered an i.p. injection of a CBD/CBD formulation in doses 1,3,10 mg/kg on day 4 post-CCI. Pain measurements and neuroinflammatory markers in the brain were subsequently assessed. Our findings suggest that thermal hyperalgesia is transiently relieved with CBD. Peripheral hypersensitivity due to CCI was transiently alleviated at 60- and 120-min post-injection but returned on days 6 and 8 post-CCI. Thus, the CCI model combined with pain hypersensitivity testing provides an ideal model system to investigate the effectiveness of a CBD-based nanotherapeutic to modify chronic neuropathic pain. Supported by 5R01DA047410-02.

#### 24. SARS-COV-2 VIRAL COMPONENTS PERSIST IN THE CIRCULATION OF INDIVIDUALS WITH POST-ACUTE SEQUELAE OF COVID-19

Ram, A.K., Ph.D.^1^, Craddock, V., MD^1^, Mahajan, A., Ph.D.^1^, Krishnamachary, B., Ph.D.^1^, Spikes, L., MD^1^, Chalise, P., Ph.D.^1^, Dhillon, N., Ph.D.^1^

^1^Division of Pulmonary and Critical Care Medicine, Department of Internal Medicine, University of Kansas Medical Center, Kansas City, KS 66160.

The immediate complications of SARS-CoV-2 infection are well defined but less is known about post-acute sequelae of COVID-19 (PASC) and now it has become a global concern. More than one third of patients with earlier SARS-CoV-2 infections show neurological illness even after the resolution of acute COVID-19. Here we examined the levels of Spike protein and viral RNA in the plasma from acute COVID-19 patients (n=116) and from patients with (8 weeks or longer after testing positive=33) and without (n=14) PASC. We detected viral RNA in 35 % and Spike protein in 62 % of hospitalized COVID-19 patients as measured using digital droplet PCR and ELISA, respectively. Interestingly, 44 % of Spike-positive patients showed the presence of Spike in the plasma derived extracellular vesicles (EVs). Further, total of 30 % PASC patients were positive for both spike and viral RNA whereas none of the individuals without PASC were positive for both. When we compared the levels during the acute and post-recovery phase of COVID-19 in the same patients (n=12), we observed that in the PASC + ve group, Spike protein and/or viral RNA increased or remained the same as in the acute phase; whereas, in the PASC-ve group, these components either decreased or were totally undetectable. We also found that circulating EVs in PASC-positive patients were more likely to have Spike protein but no presence of viral RNA. In conclusion, Spike and/or viral RNA fragments persist in the recovered COVID-19 patients with PASC up to 1 year or longer after acute SARS-CoV-2 infection and may contribute to PASC pathophysiology. Supported by National Institute of Health (NIH) grants R01 HL129875 awarded to N.K.D.

#### 25. CLOCK-DEPENDENT IL-1B AUTO-REGULATORY LOOP REGULATES INFLAMMATION IN GLIOMA

Sharma, S. ^1^, Gowda, P.^1^, Lathoria, K.^1^, Patrick, S.^1^, Mitra K.M.^2^, Sen, E.^1^

^1^Cellular and Molecular Neuroscience, National Brain Research Centre, Gurugram, 122051

^2^Depatment of Physics, Indian Institute of Technology, Mumbai, 110003.

A dysregulated circadian rhythm affects metabolism and inflammation in tumors. As this interaction is not well understood, we investigated the aberrant metabolism-inflammation-circadian rhythm axis in glioma. An increase in tumor metabolite lactate was concomitant with increase in proinflammatory cytokine IL-1b and circadian factors Clock and Bmal1. Knockdown of Bmal1 and Clock resulted in a decrease in LDHA, IL-1b, lactate release and proinflammatory cytokines. Site-directed mutagenesis, luciferase reporter and sequential chromatin immunoprecipitation assays showed a lactate and IL-1b mediated recruitment of Clock/Bmal1 to E-box sites on LDHA and IL-1b promoters. The lactate-IL-1b-Clock/Bmal1 loop positively affected a number of pathways including cell cycle, DNA damage and cytoskeletal organization in glioma. To further elucidate the implication of this complex interplay, we developed a mathematical model that quantitatively describes this LDHA-IL-1b-Clock/Bmal1 (LIC) circuit. TCGA data analysis showed the exsistence of this loop in other cancers as well. Additionally, the components of the LIC loop were associated with worse prognosis in glioma patients. Pharmacogenomic analysis showed that the genes of the LIC loop are correlated with clinically actionable genes indicating their sensitivity to anti-cancer drugs. Our findings provide evidence of a feed-forward lactate-IL-1b-Clock/Bmal1 loop that drives tumor progression by driving aberrant metabolism and inflammation in glioma. Supported by Department of Biotechnology.

#### 26. BLOOD-BRAIN BARRIER PERICYTES, OCCLUDIN, AND HIV-1 INFECTION

Torices, S., Ph.D.^1^, Mendoza, L., BS^2^, Daniels, D., MS^1^, McKenzie, S., BS^1^, Daire, L., BS^2^, Naranjo, O., Ph.D.^1^, Teglas, T., Ph.D.^1^, Fattakhov, N., Ph.D.^1^, Toborek, M., MD, Ph.D.^1^

^1^University of Miami, Miller School of Medicine, Miami, FL 33136

^2^American Heart Association, Dallas, TX

Occludin (ocln) is a tetraspan redox-sensitive protein associated with tight junctions of the blood brain barrier (BBB). It plays a key role in maintaining the integrity of the BBB and it has been described as a multifunctional protein. By high-throughput RNA sequencing, we identified changes in gene expression-related to ocln modifications in human brain pericytes, one of the main regulatory cells of the BBB integrity. After ocln silencing, we found an alteration in several genes of the antiviral retinoic-acid-inducible gene-1 (RIG-1) signal pathway and the immune system response pathway when compared with non-treated cells. Several studies have demonstrated the capacity of ocln to control HIV-1 infection of human pericytes. Here, we show an ocln antiviral role in HIV infection *in vivo* and *in vitro*. Mechanistically, we provide evidence that cellular ocln level can modulate HIV-1 infection by controlling the expression levels of several INF-stimulated genes such as ISG15, MX2, or IFIT1 through JAK/STAT signaling by influencing interferon regulatory factors (IRF) expression levels and STAT-1 activation. In addition, regulation of HIV infection by ocln level was confirmed in a mouse model. Unexpectedly, we observed for the first time that ocln deficient mice present neurological deficits when compared to heterozygous or wild type mice. Overall, these results are important to a better understanding of the molecular mechanisms for viral infection in the brain and describe previously unrecognized role of the protein ocln as a key factor in the control of innate immune response. Supported by Miami Center for AIDS Research (CFAR) (P30AI073961), AHA (20POST35211181), and NIH (MH098891, MH072567, HL126559).

#### 27. MICROBIOME IMPLICATIONS IN MORPHINE WITHDRAWAL.

Truitt, B.^1^

^1^Surgery, University of Miami, Miami, FL 33130.

The ongoing opioid epidemic has left millions of people to suffer from opioid use disorder due to the over prescription of highly addictive substances. Chronic opioid exposure can lead to dependance where the absence of the drug results in negative symptoms of withdrawal, often driving people to continued drug use, and few therapeutic strategies are available to combat the cycle of addiction and the severity of morphine withdrawal. This study investigates the microbiome as a potential therapeutic target for morphine withdrawal, as the gut dysbiosis caused by morphine use has been proven to contribute to other aspects of addiction, such as tolerance. Results showed that the microbiome during morphine withdrawal beings to recover from the morphine induced dysbiosis, however there continues to be a disruption in the alpha and beta diversity as well as the abundance of Gram-positive bacteria that may still contribute to the severity of morphine withdrawal. This microbial disruption cannot occur in germ-free mice, that lack a microbiome, these mice did not develop somatic withdrawal symptoms, indicating the microbiome is necessary for the development of withdrawal. Additionally, TLR2 but not TLR4 whole body knock-out models displayed less withdrawal severity, suggesting the microbiome modulates morphine withdrawal through a Gram-positive, TLR2 mediated mechanism. Supported by T32.

#### 28. ELUCIDATING THE ANTI-INFLAMMATORY ROLE OF SCFA IN JEV-MEDIATED NEUROINFLAMMATION IN MICE.

Siva Venkatesh, I.P., MS^1^

^1^Cellular and molecular neuroscience, National Brain Research Centre, Gurugram, 122051.

Short chain fatty acids (SCFAs), majorly, acetate, propionate and butyrate are metabolites generated by anaerobic fermentation of complex carbohydrates by the bacteria in the gastrointestinal tract. Japanese encephalitis virus (JEV), a neurotropic flavivirus, causes severe neuro-inflammation and neuronal death leading to various neurological symptoms and eventually death. Emerging evidence suggests anti-inflammatory nature of SCFAs in multiple neurodegenerative disorders. In this study we aim to elucidate the possible role of SCFAs in JEV induced neuro-inflammation. Post-natal day 10 BALB/c mice were randomly assigned to groups. Each group was intraperitoneally injected either with SCFA mixture (Acetate:propionate:butyrate = 35 mM:15 mM:10 mM) or PBS for a period of 7 days, followed by JEV infection. All mice were observed for onset and progression of symptoms. Upon reaching terminal illness, mice were sacrificed by transcardial perfusion and the brain tissue was collected for further analysis. SCFA treated mice showed delayed onset of symptoms upon infection as opposed to the PBS treated animal, thereby increasing the survival by 3 days. Significant downregulation of inflammatory cytokines TNF-α, MCP-1, IL-6, IFN-Υ in SCFA treated group relative to JEV infected control group was observed. Tissue section analysis revealed reduced glial activation in SCFA treated animals with respect to the JEV animals. Through our study, we establish SCFA as a possible supplementary intervention in attenuating JEV-mediated neuro-inflammation. Supported by JCB/2020/000037.

#### 29. MORPHINE-INDUCED INCREASE IN INTESTINAL IGA AND IGG IS A CONSEQUENCE OF A DYSBIOTIC MICROBIOME

Vitari, N. ^1^, Singh, S.^2^, Abu, Y.^1^, Tao, J., Ph.D.^2^, Jalodia, R., Ph.D.^2^, Kolli, U., Ph.D.^2^, Sharma, U., Ph.D.^2^, Roy, S., Ph.D.^2^

^1^Microbiology and Immunology, University of Miami, Miami, FL 33136

^2^Department of Surgery, University of Miami, Miami, FL 33136.

Morphine is commonly prescribed in the U.S. to manage moderate to severe pain. The side effects of morphine include modulation of the immune system, promotion of intestinal microbial dysbiosis, and disruption of the gut epithelial barrier. Describing the mechanisms of how morphine induces these changes can result in potential therapeutics. Microbiome composition is regulated by intestinal antibodies, specifically by secretory IgA and IgG produced through either T cell-dependent and -independent pathways. Morphine-induced microbial dysbiosis is dependent on the immune system, but the mechanism remains elusive. Additionally, morphine-induced intestinal microbial dysbiosis is rapid, occurring within 24 h of morphine exposure. Using ELISA assays for intestinal IgA and IgG, we analyzed the intestinal luminal content of mice following 24 h after morphine or placebo treatment with in tact or depleted microbiome. Here, we report an increase in the concentration of intestinal antibodies (IgA, IgG2b, and IgG3) in the intestinal luminal content following 24 h of morphine exposure. Interestingly, this increased IgA concentration is abrogated if the microbiome is depleted prior to morphine exposure. We conclude this increase in intestinal antibodies is dependent on the microbiome. Further work aims to identify whether this increase is driving the morphine-induced microbial dysbiosis, or if the microbial dysbiosis is the source of the increased antibodies.

